# Blatter Radicals as Bipolar Materials for Symmetrical Redox-Flow Batteries

**DOI:** 10.1021/jacs.1c13543

**Published:** 2022-03-08

**Authors:** Jelte S. Steen, Jules L. Nuismer, Vytautas Eiva, Albert E. T. Wiglema, Nicolas Daub, Johan Hjelm, Edwin Otten

**Affiliations:** † Stratingh Institute for Chemistry, University of Groningen, Nijenborgh 4, 9747 AG Groningen, The Netherlands; ‡ Molecular Materials and Nanosystems & Institute for Complex Molecular Systems, Eindhoven University of Technology, 5600 MB Eindhoven, The Netherlands; § Department of Energy Conversion and Storage (DTU Energy), Technical University of Denmark, Fysikvej, Building 310, 2800 Kgs Lyngby, Denmark

## Abstract

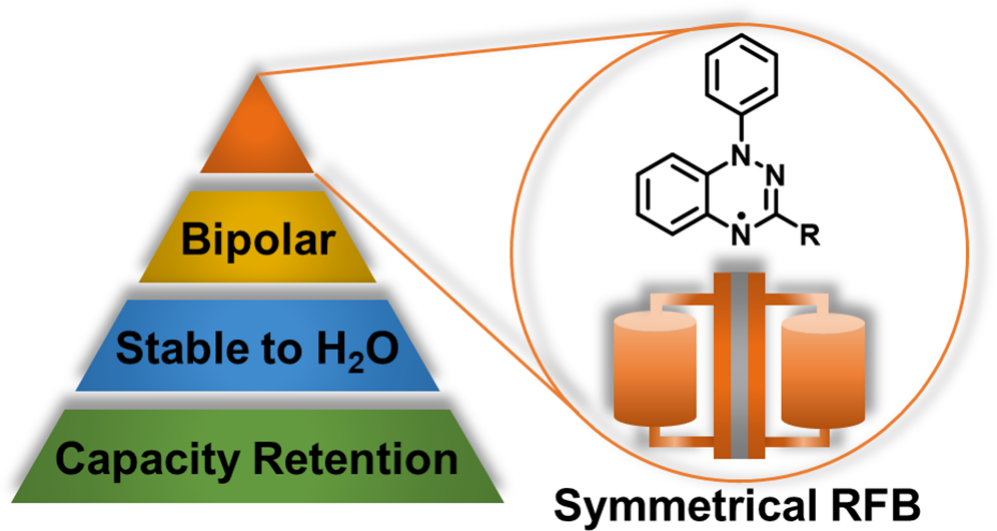

Redox-active organic molecules are promising charge-storage materials for redox-flow batteries (RFBs), but material crossover between the posolyte and negolyte and chemical degradation are limiting factors in the performance of all-organic RFBs. We demonstrate that the bipolar electrochemistry of 1,2,4-benzotriazin-4-yl (Blatter) radicals allows the construction of batteries with symmetrical electrolyte composition. Cyclic voltammetry shows that these radicals also retain reversible bipolar electrochemistry in the presence of water. The redox potentials of derivatives with a C(3)-CF_3_ substituent are the least affected by water, and moreover, these compounds show >90% capacity retention after charge/discharge cycling in a static H-cell for 7 days (ca. 100 cycles). Testing these materials in a flow regime at a 0.1 M concentration of the active material confirmed the high cycling stability under conditions relevant for RFB operation and demonstrated that polarity inversion in a symmetrical flow battery may be used to rebalance the cell. Chemical synthesis provides insight in the nature of the charged species by spectroscopy and (for the oxidized state) X-ray crystallography. The stability of these compounds in all three states of charge highlights their potential for application in symmetrical organic redox-flow batteries.

## Introduction

The increased adoption of renewable energy sources such as wind and solar, which are inherently only available on an intermittent basis, necessitates cheap and scalable storage solutions in order to ensure a stable future energy supply.^1^ Of the various battery technologies currently available, redox-flow batteries (RFBs) are attractive for large-scale stationary applications due to their scalability, decoupling of power and energy, and simplicity of design.^2^ Current RFB technologies that are reaching commercial applications are based on the redox chemistry of transition-metal ions (e.g., vanadium or iron),^3^ but widespread implementation is hampered, among others, by low volumetric charge densities, concerns regarding environmental impact, and high system cost. Electrolytes that are based on less harmful, earth-abundant materials are a prime target for next-generation RFBs. In recent years, there has been a surge in the development of organic charge-storage materials targeting both aqueous and nonaqueous electrolytes, but the long-term stability of organic compounds, during either battery cycling or storage, has proven difficult to achieve and material decomposition is an important factor in the capacity fade.^4^ The vast majority of current organic RFB designs utilize different redox-active compounds on both the positive and negative sides of the battery. Such asymmetric electrolyte compositions impose stringent selectivity requirements on the separator membrane, as crossover of the electrolyte results in irreversible capacity fade.^5^ Several ways to minimize active material crossover have been put forward: for example with improved membrane architectures,^6^ with size exclusion,^7^ or using immiscible electrolytes.^8^ Conceptually, the use of symmetrical cell designs (with identical posolyte/negolyte composition in the discharged state) presents an additional strategy to mitigate the effect of membrane crossover.^9^ Organic RFBs with symmetrical battery chemistries have been prepared by combining two redox-active units via a linker (“combi-molecules”),^10^ using bipolar small-molecule compounds with complementary redox units,^9a,11^ or using helical carbenium ions.^12^ In addition to closed-shell compounds,^13^ several classes of stable organic radicals are known to possess bipolar electrochemistry and are thus of potential interest as battery materials.^14^ Examples of bipolar organic radicals that have been explored to date include nitronyl nitroxides^15^ and verdazyls,^16^ but these systems show relatively rapid degradation^17^ in the charged state and high capacity retention has yet to be achieved. Here we show that 1,2,4-benzotriazin-4-yl radicals (commonly known as Blatter radicals)^18,19^ combine tunable redox potentials and high stability in all relevant redox states necessary for application as redox-active components in RFBs. The cell potentials of derivatives with a CF_3_ substituent on the triazinyl ring are especially insensitive to water (up to 33 vol % of water in acetonitrile), which holds promise for this class of materials in energy storage systems based on earth-abundant elements.

## Results and Discussion

**Scheme 1:**
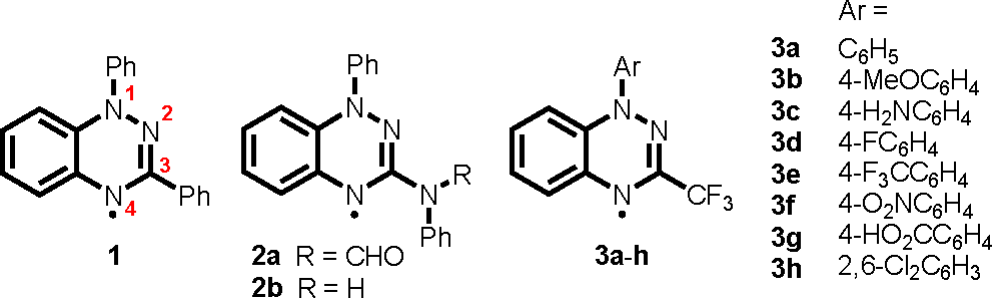
Blatter Radicals Used in This Work

The 1,2,4-benzotriazin-4-yl radicals described here were prepared on the basis of literature procedures. The parent Blatter radical (**1**; Scheme 1) was synthesized via the catalytic oxidation of *N*-phenyl amidrazone.^20^ Derivatives with N-substitution at C(3) (**2a**,**b**) were synthesized by hydrolysis of Nitron,^21^ whereas the Cu-mediated coupling route by Koutentis et al.^22^ provided access to C(3)–CF_3_ radicals that differ in the N(1) aromatic group (**3a**–**h**). Radical **3c** was obtained in quantitative yield by Pd/C-catalyzed hydrogenation of the *p*-NO_2_ group in **3f**. The solid-state structures of **3c**,**h** were determined by single-crystal X-ray diffraction. As expected for Blatter radicals,^23^ all compounds (except **3g**; *vide infra*)^24^ showed cyclic voltammetry in anhydrous acetonitrile that is consistent with bipolar behavior: both oxidation and reduction to form the corresponding closed-shell cation and anion are reversible, indicating that the three different states of charge are chemically stable, at least on the time scale of voltammetric experiments. The redox potentials for the series of compounds in acetonitrile are reported in Table 1. A comparison of the redox potentials of compounds **1**, **2a**,**b** and **3a**, which differ in the C(3) substituent, shows that both couples are sensitive to the electron-donating/-withdrawing properties of the group present at C(3), but not by the same amount. This allows tuning of the (calculated) cell potential, *E*_cell_ = *E*_1/2_(**x**^0/+^) – *E*_1/2_(**x**^0/–^), and leads to the highest value (*E*_cell_ = 1.12 V) for the derivative with an electron-withdrawing CF_3_ group at that position (**3a**). An analysis of diffusion coefficients (*D*) and standard rate constants (*k*^0^) from the CV data at different scan rates (using Randles–Sevcik and Nicholson methods, respectively) gives similar values for both redox couples in this series of bipolar Blatter radicals (*D* ≈ 10^–5^ cm^2^ s^–1^; *k*^0^ ≈ 10^–2^ cm s^–1^; see Table S5). These values are comparable to those of other nonaqueous organic RFB systems.^25^

**Table 1:** Cyclic Voltammetric Data (V) for 1 mM Blatter Radicals in MeCN and a MeCN/H_2_O Mixture (2/1 v/v)^a^

compound	*E*_1/2_(**x**^0/–^)	*E*_1/2_(**x**^0/+^)	calcd *E*_cell_ (MeCN)	calcd *E*_cell_ (MeCN/H_2_O)
**1**	–1.24	–0.20	1.04	0.50
**2a**	–1.07	–0.09	0.98	0.66
**2b**	–1.24	–0.31	0.93	0.44
**3a**	–1.03	0.09	1.12	0.81
**3b**	–1.09	0.05	1.14	0.82
**3c**	–1.15	–0.06	1.09	0.74
**3d**	–1.02	0.12	1.13	0.84
**3e**	–0.87	0.17	1.03	0.82
**3f**	–0.71	0.20	0.91	0.77
**3g**	–1.04^b^	0.24		
**3h**	–0.99	0.32	1.32	1.0

a The redox potentials in 2/1 MeCN/H_2_O that are used to calculate *E*_cell_ in that solvent mixture are shown in Table S3.

b The reduction of **3g** is irreversible, and the peak potential is given.

Within the series of CF_3_ Blatter radicals **3** with varying N(1)–Ar *para* substituents, the presence of electron-donating groups (**3b**, OMe; **3c**, NH_2_) shifts both redox potentials to more negative potentials, as expected, and the opposite is observed for electron-withdrawing groups (**3d**, F; **3e**, CF_3_; **3f**, NO_2_; see Table 1). Moreover, the introduction of 2,6-dichloro substituents on the N(1)–Ar ring provides an additional increase in the calculated cell potential of ca. 0.2 V to give an *E*_cell_ value of 1.32 V for compound **3h**.

**Figure 1:**
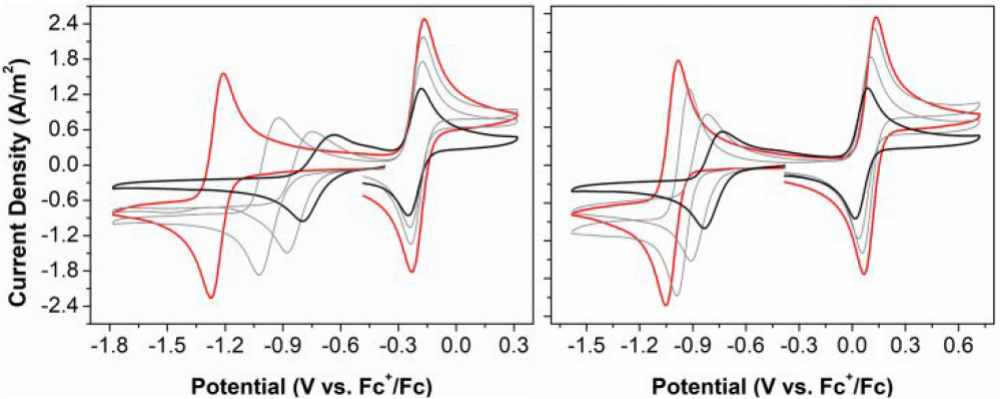
Cyclic voltammograms of 1 mM compound **1** (left) and 1 mM **3a** (right) in acetonitrile (red trace) and in acetonitrile with 18.5 M water (black trace; intermediate amounts of 3.0 and 9.0 M are shown in gray), measured at a GC working electrode with 0.1 M [Bu_4_N][PF_6_] as the supporting electrolyte.

Cyclic voltammetry with stepwise addition of up to 33 vol % (ca. 18.5 M) of H_2_O to the acetonitrile evaluated the effect of water on the redox chemistry of Blatter radicals. A comparison of the voltammetry of **1** and **3a** shows that in both cases the oxidation of the radicals to the cations is only marginally affected by the addition of water (Figure 1), and this extends to the other compounds (see the Supporting Information). The reduction to the anions, *E*_1/2_(**x**^0/–^), on the other hand, shifts to more positive potentials, and the overall result is a decrease of 0.54 V in the maximum calculated cell potential for **1** (*E*_cell_ = 0.51 V in a 2/1 MeCN/H_2_O mixture). For the CF_3_-substituted compounds the redox potentials of the **3**^0/–^ couple, on the other hand, are substantially less sensitive to water and larger cell potentials are retained (up to 1.0 V for **3h**; see the Supporting Information). We attribute the change in *E*_1/2_(**x**^0/–^) to the stabilizing effect of hydrogen-bonding interactions on the reduction products.^26^ The observation that this shift depends on the nature of the C(3) substituent (and is smallest for the CF_3_-substituted radicals **3**) suggests that the Brønsted basicity of the closed-shell anions **3**^**–**^ is an important criterion. Recent work by Wang and co-workers also demonstrated the importance of p*K*_a_ on reversibility in fluorenone-type RFB negolytes,^27^ and it thus appears that molecular engineering can be used to further optimize the cell potential in bipolar Blatter radicals.

**Figure 2:**
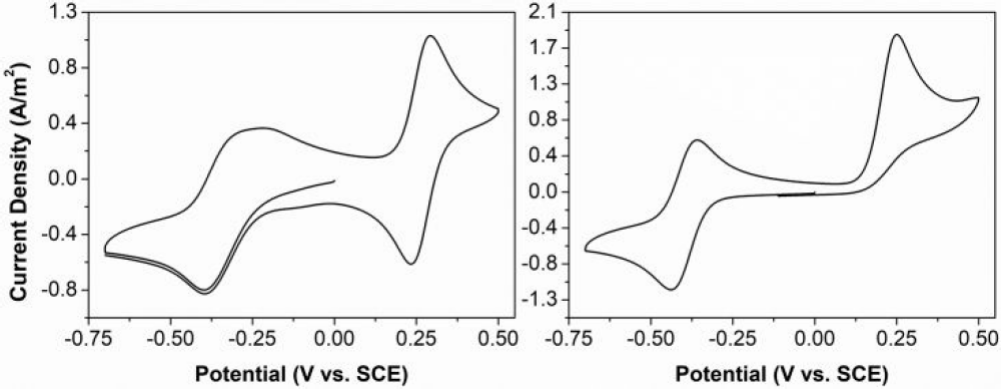
Cyclic voltammograms of compound **3g** in water with 1 M KCl (left) and after addition of Na_2_CO_3_ to pH 10 (right).

Radical **3g**, which has a carboxylic acid substituent, was sufficiently soluble to record its cyclic voltammogram in water alone (∼1 mM **3g**; 1 M KCl as supporting electrolyte). Under those conditions the CV of **3g** shows a somewhat broadened reduction wave, but both redox processes are (quasi-)reversible with *E*_cell_ ≈ 0.60 V (Figure 2). Addition of Na_2_CO_3_ to pH 10 increased the solubility of **3g** (as the deprotonated carboxylate form) and a better defined, cathodically shifted **3g**^0/–^ redox couple was obtained. The oxidation to the cation **3g**^**+**^ was irreversible, indicating that OH^–^ has a detrimental influence on the stability of the oxidized state. This is similar to the instability observed for quinones, for which nucleophilic attack by H_2_O or OH^–^ has been identified as one of the key decomposition pathways.^28^

**Figure 3:**
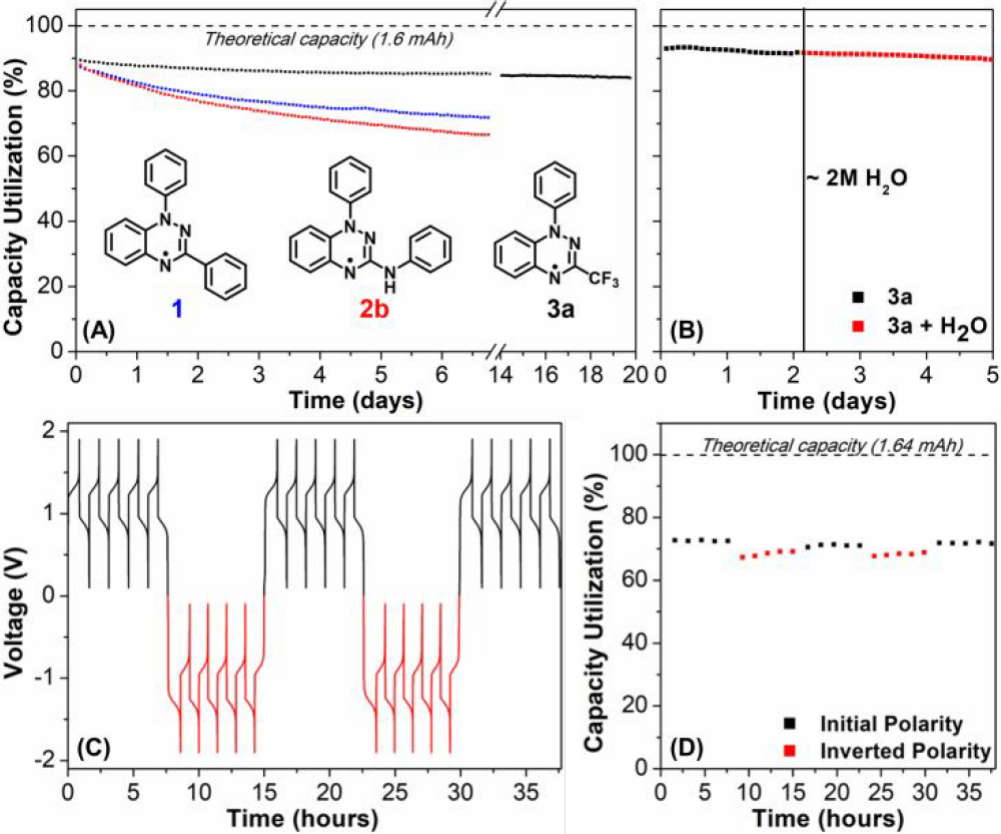
(A) Normalized discharge capacities of symmetric H-cell battery cells with 12 mM solution of Blatter radical **1**, **2b**, and **3a** in anhydrous acetonitrile with 0.3 M [Bu_4_N][PF_6_] (current density 1.0 mA/cm^2^). (B) Addition of water after 23 cycles (indicated by a vertical black line, ca. 2 M) does not affect the stability of a battery consisting of **3a**. (C) Voltage vs time curves of a symmetrical H-cell battery cell with 6 mM **3a** with polarity inversion every 5 cycles. (D) Normalized discharge capacity of a symmetrical H-cell battery cell with 6 mM **3a** with polarity inversion every 5 cycles.

The battery performance of Blatter radicals was evaluated by cycling in a stirred H-cell with a porous glass frit. The volume of electrolyte used on each side was 5 mL, and the solutions were magnetically stirred to ensure efficient mixing (see the Supporting Information for details). A symmetrical (“poleless”) battery was constructed using **1** (12 mM) as the charge-storage material in anhydrous MeCN with 0.3 M [Bu_4_N][PF_6_] as the supporting electrolyte, which corresponds to a theoretical capacity of 1.6 mAh. The battery was charged/discharged (current ±1.6 mA, cutoff voltages of 1.8 and 0.1 V) in cycles of 1 h, and the changes in capacity were monitored over the course of several days (Figure 3A). Although the coulombic efficiency remains high throughout (>99%), a gradual decrease in capacity is observed such that after approximately 10 days (157 cycles) the remaining battery capacity is ca. 80% of the initial value (average discharge capacity fade of 2.0% per day). When a similar battery containing **2a** (9 mM, current ±1.22 mA) as the electroactive component is cycled, the capacity drops rapidly in the first cycles to ca. 60% and then continues to fade at a rate that is still appreciably higher than that for **1** (see Figure S57). Visual inspection of the H-cell after the first charge/discharge cycle shows the posolyte and negolyte to be different, suggesting that the system does not return to a symmetrical composition in the discharged state. UV–vis absorption spectra of both sides (in the discharged state) after cycling for 44 h (27 cycles) shows that the posolyte remains unchanged, but the starting material **2a** in the negolyte solution is fully consumed. The broad absorbance maximum of **2a** (centered around 550 nm) is replaced by a new band at λ_max_ = 596 nm, which is indicative of the formation of **2b** on the basis of a comparison with an authentic sample (see Figure S58). Although the conversion of **2a** to **2b** under strongly alkaline conditions has been reported,^21^ these results suggest that, despite the stability of **2a** on the CV time scale, reduction to the closed-shell anion **2a**^**–**^ results in its decomposition to **2b** and perhaps other, not yet identified, products. Starting with pure compound **2b** (12 mM, current ±1.6 mA, cutoff voltages of 1.7 and 0.1 V) in both electrolyte compartments leads to a discharge capacity fade rate that is gradual but somewhat larger than in **1** (3.6% per day).

The CF_3_-substituted Blatter radical **3a** (12 mM in MeCN) was cycled in an H-cell for a total of 20 days (277 cycles; current ±1.6 mA, cutoff voltages of 1.8 and 0.1 V). This resulted in a high capacity retention of >94%, corresponding to an average fade rate of only 0.3% per day, or 0.02% per charge/discharge cycle (Figure 3A). Similar results were obtained for the other derivatives **3**, highlighting the remarkable stability of this class of Blatter radicals. The only exception is **3h**, which loses almost 40% of the capacity in the first week of cycling and then shows accelerated decomposition to reach 0% after ca. 12 days (see Figure S68). The *p*-NH_2_-substituted derivative **3c** shows even higher stability in comparison to **3a**, and virtually no decay is discernible after 111 charge/discharge cycles (7 days; see Figure S64). The “poleless” nature of a battery using **3a** (6 mM in MeCN) as the active material was demonstrated by carrying out a cycling experiment in which polarity inversion was applied every 5 cycles, showing 98.5% capacity retention after 25 cycles (37.6 h) (Figure 3C,D and Figure S69). Furthermore, the H-cell experiment was repeated at a higher concentration of active material (∼4-fold increase, 50 mM of **3a**), which also resulted in good capacity retention, indicating that higher concentrations of the active material do not result in a significantly faster capacity fade (Figure S70).

A comparison of the cycling data for **3a**–**f** at 12 mM concentration during a 7-day experiment shows that the nature of the N(1)–Ar *para* substituent has no (detrimental) influence on the stability, suggesting that this is an ideal position to engineer desirable properties such as (aqueous) solubility in future studies. The cycling stability of **3a** and the observation that reversible voltammetry is retained in aqueous acetonitrile prompted the addition of H_2_O (ca. 2 M) to both sides of the H-cell (Figure 3B). As anticipated on the basis of the cyclic voltammetry data, the overall cell cycling behavior and the capacity fade are not affected by the presence of water, and molecular decomposition is also slow in all states of charge under aqueous conditions.

**Figure 4:**
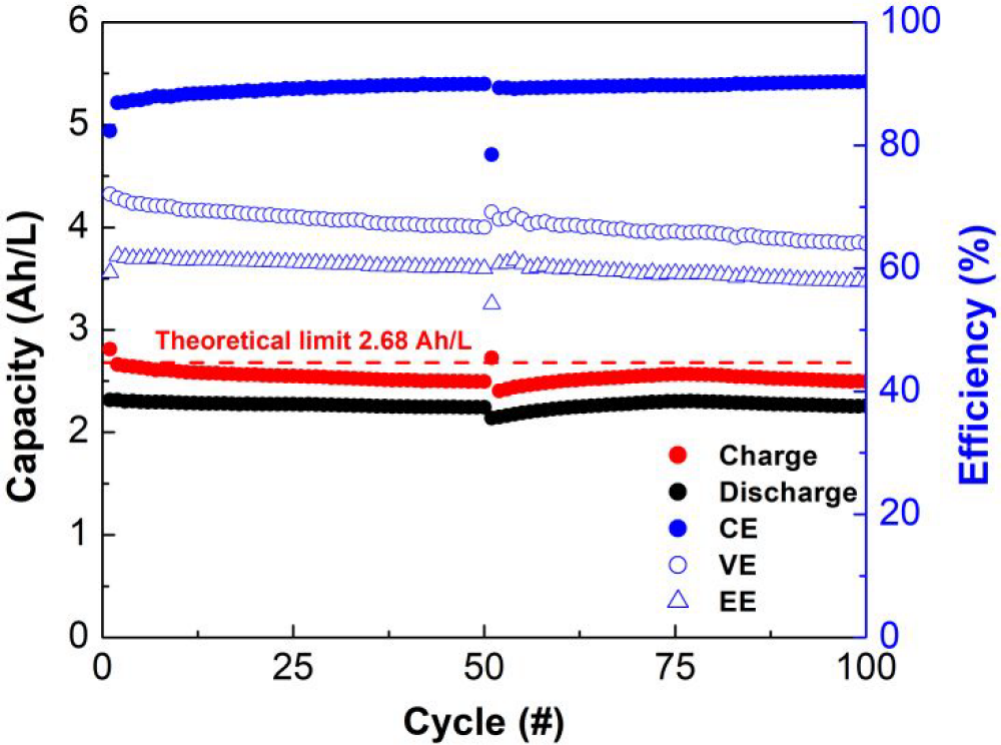
Symmetrical flow cell cycling of 0.1 M of **3a** in 0.3 M [Bu_4_N][PF_6_]/acetonitrile as both posolyte and negolyte. Polarity inversion was performed after 50 charge/discharge cycles. The total duration was 32.3 h for 100 cycles.

On the basis of the promising charge–discharge H-cell results, **3a** was selected for higher-concentration experiments in a flow cell. Symmetrical cell cycling was performed in a zero-gap flow cell^29^ separated by a Daramic 175 membrane (see the Supporting Information for details). Both the posolyte and negolyte reservoirs were filled with 6 mL of 0.1 M of **3a** in 0.3 M [Bu_4_N][PF_6_]/acetonitrile. Constant-current cycling was conducted at 89.25 mA (current density of 35 mA/cm^2^) with voltaic cutoffs at 1.7 and 0 V, resulting in a capacity utilization of 86% (theoretical capacity of 2.68 Ah/L). The cell was cycled 50 times with a capacity retention of 97% (Figure 4). The Daramic membrane is a nonselective porous separator that has low ohmic resistance but allows crossover of both charged and neutral species relatively readily. At the current density used (35 mA/cm^2^), we were able to access charge capacities close to the theoretical value and ca. 90% was recovered upon discharge, in line with the relatively low coulombic efficiencies due to crossover-induced self-discharge when a microporous separator was used.^30^ To evaluate whether the decrease in capacity observed in the first 50 cycles could be the result of volume and/or concentration imbalances between the two electrolytes, we inverted the polarity of the battery at that point and ran another 50 charge/discharge cycles. As shown in Figure 4, this indeed results in capacity recovery (to 99.5% of the initial capacity utilization at cycle 75) and demonstrates that polarity inversion in these bipolar systems can be used to rebalance the cell in a greatly simplified manner in comparison to physical rebalancing methods that require battery disassembly.^5b,30^ It should be noted that, in contrast to “compositionally symmetrical cells” prepared by premixing of (different) posolyte and anolyte materials,^31^ the use of intrinsically bipolar materials such as **3a** allows the utilization of *all* electroactive material in solution. Potentiostatic electrochemical impedance spectroscopy (PEIS) measurements of the flow cell before and after cycling did not indicate a significant increase in resistance. With an overall capacity retention of 98% after 100 cycles (0.02% capacity fade per cycle, 1.5% capacity fade per day) and average coulombic, voltaic, and energy efficiencies of 89%, 67%, and 60%, respectively, the flow cell studies demonstrate the excellent stability of **3a** in all three states of charge. The battery performance is similar to that of other symmetrical RFBs reported in the literature; however, the battery outperforms most RFBs with regard to cycling stability (Table S6).

**Scheme 2:**
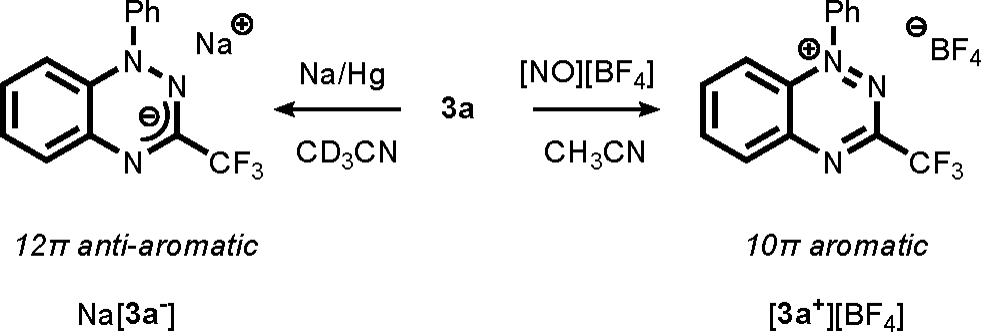
Synthesis of the Charged Species **3a**^–^ and **3a**^+^

**Figure 5:**
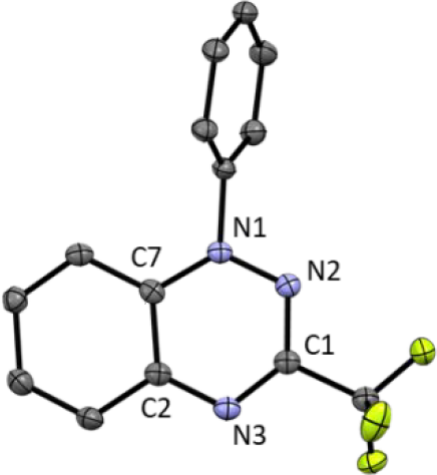
Molecular structure of [**3a**^**+**^][BF_4_] (50% probability ellipsoids, hydrogen atoms and the BF_4_ anion omitted for clarity).

While neutral Blatter radicals have been studied in a variety of scientific fields (e.g., molecular electronics, magnetochemistry, biochemistry),^19^ the chemical stability of these compounds in the oxidized and reduced state, which is of key importance in energy storage applications, has received scant attention in the literature. This aspect was explored by treating radical **3a** on the NMR scale (CD_3_CN solution) with the oxidant [NO][BF_4_] (Scheme 2). The corresponding closed-shell cation **3a**^**+**^ was obtained with characteristic spectral features including downfield-shifted ^1^H NMR signals for the aromatic moieties (δ 7.9–8.8 ppm) and a ^19^F resonance at −69.4 ppm. On a preparative scale, [**3a**^**+**^][BF_4_] was obtained as a yellow-green powder in 97% isolated yield. Recrystallization by vapor diffusion of ether into an acetonitrile solution afforded crystals of [**3a**^**+**^][BF_4_] suitable for X-ray diffraction (Figure 5). A comparison of the metrical data (see Table S3) of radical **3a**(22) and the cation **3a**^**+**^ indicates that, while both have a planar heterocyclic core, oxidation is accompanied by a pronounced decrease in the N1–N2 bond length from 1.370(2) Å in **3** to 1.318(1) Å in **3a**^**+**^, and also the C–N bonds to the annulated ring shorten to 1.359(2) Å (C7–N1) and 1.341(2) Å (C2–N3) in **3a**^**+**^ (in **3**: 1.390(2) and 1.387(3) Å, respectively). Together with the long/short bond alternation in the annulated C_6_ ring, these metrical parameters validate that **3a**^**+**^ has 10π aromatic character analogous to that of naphthalene and its derivatives.^32^

In a similar manner, the anion **3a**^**–**^ (formally a 12π antiaromatic compound) was obtained in quantitative yield on the NMR scale by reduction with Na/Hg in CD_3_CN (Scheme 2). As expected for a more electron rich compound, the ^1^H NMR resonances were shifted upfield by ca. 1.5 ppm to δ 6.1–7.3 ppm (^19^F NMR: −72.3 ppm). When they are kept in a Teflon-sealed NMR tube under an N_2_ atmosphere, both of these charged species are stable and show no sign of decomposition over the course of 7 days. In agreement with the battery cycling data, the NMR spectra also remain unchanged upon addition of H_2_O to either **3a**^**+**^ or **3a**^**–**^. Compound **3a**^**+**^ is also stable under ambient conditions, whereas **3**^**–**^ is quickly oxidized in air. UV–vis absorption spectra of the series **3a**^**–/0/+**^ show that the broad long-wavelength bands in **3a** that give rise to the characteristic red color (λ_max_ = 479 nm, shoulder extending to ca. 600 nm) are blue-shifted upon oxidation/reduction to λ_max_ = 420 and 402 nm for **3a**^**+**^ and **3a**^**–**^, respectively. The changes in the electronic absorption spectra make this system suitable for online battery monitoring, which is useful going forward to obtain molecular insight into performance characteristics in more extensive flow studies.

## Conclusions

In conclusion, we have shown that 1,2,4-benzotriazin-4-yl radicals combine bipolar electrochemistry with high chemical stability in all states of charge, which renders these compounds promising energy-storage materials for symmetric redox-flow batteries. In particular, the derivatives with a C(3)–CF_3_ substituent (compounds **3**) show minimal capacity fade during battery cycling; the stability of the charged states (**3a**^**+**^ and **3a**^**–**^) was confirmed through an independent chemical synthesis. Cyclic voltammetry and cell cycling studies in aqueous MeCN solution indicate that the electrochemical reversibility and chemical stability is not affected by the presence of water, but the reduction potential is anodically shifted to decrease the (theoretical) cell potential. The data presented here indicate that this is due to hydrogen-bond stabilization of the anionic reduction products by H_2_O. The *E*_cell_ drop can be (partially) mitigated by decreasing the H-bond acceptor character of the anions, providing acceptable (∼1 V) cell potentials also in aqueous electrolytes. In this regard, it is noted that a high cycling stability is retained regardless of the *para* substituent on the N(1)–Ar ring, suggesting that this position is a prime target to further engineer desirable properties, including charged groups for water solubility. Testing a representative radical (**3a**) in a flow regime at 0.1 M concentration confirmed the high cycling stability and demonstrated that polarity inversion in a symmetrical flow battery may be used to rebalance the cell. While further research is needed to investigate the long-term stability, our work provides a proof of concept for the use of Blatter radicals in electrochemical energy-storage applications and a stepping stone toward innovative all-organic, symmetrical battery chemistries.

## Experimental Section

### Cyclic Voltammetry

Cyclic voltammetry (CV) was performed using a three-electrode configuration comprising of a Pt-wire counter electrode, a Ag/Ag^+^ (0.01 M AgPF_6_ in 0.1 M [Bu_4_N][PF_6_]/acetonitrile) junction reference electrode, and a GC-disk working electrode (CHI104, CH Instruments, diameter 3 mm). The GC working electrode was polished before the experiment using an alumina slurry (0.03 μm), rinsed with distilled water, and subjected to brief ultrasonication to remove any adhered alumina microparticles. The CV data were referenced to ferrocene in acetonitrile. For measurements in H_2_O, an SCE reference electrode was used.

### H-Cell Cycling

Charge–discharge tests of the Blatter radicals were performed in a custom H-cell with high surface area to volume ratio. The cell consisted of two electrolyte chambers separated by a glass frit (porosity 5, ∼1.6 cm^2^) to minimize crossover. Reticulated vitreous carbon (Duocel, 45 ppi) was used as electrodes with an interelectrode distance of about 20 mm. Generally, for the battery tests, the electrolyte chambers were loaded with 5 mL of 12 mM active species in 0.3 M [Bu_4_N][PF_6_]/MeCN and stirred continuously at 1400 rpm. The current was set such that theoretical charging and discharging times were 1 h each (1*C* rate). Current densities were estimated using the membrane area size. An ohmic resistance of about 250 Ω was measured in all tests.

### Flow-Cell Cycling

Measurements under flow conditions were carried out using a zero-gap flow cell.^29^ The oven-dried battery was assembled outside the glovebox. A combination of a graphite charge-collecting plate and two layers of a nonwoven carbon paper electrode with an area of 2.55 cm^2^ (Sigracet 29AA) was put on either side of the flow cell. A ±10% compression of the felt was achieved by the use of Gore-tex ePTFE gaskets. The two half-cells were separated by a Daramic 175 porous membrane. The gasket window provided for an exposed area of the membrane which was used as the active area of the flow cell. The cell was connected to a peristaltic pump (Cole-Parmer) by Masterflex C-flex Ultra pump tubing using a flow rate of 20 mL/min. The catholyte and anolyte reservoirs were filled with 6 mL of a solution of redox materials and electrolyte salt in the reported concentrations. Before the measurement was started, the cell was pretreated by flowing the solution through the cell for 30 min. Once the membrane was fully wetted as evidenced by impedance measurements, the cycling was started. Galvanostatic charge/discharge cycling was performed using currents of ±89.25 mA (±35 mA/cm^2^) for the batteries shown in Figure 4 and Figure S71 with potential cutoffs at +1.7 and 0 V. Potentiostatic electrochemical impedance spectroscopy (PEIS) measurements were performed at various stages of charge from 500 kHz to 50 Hz using a 10 mV sine perturbation. A polarization measurement was collected at full SOC and ranging from −12.75 to −255 mA. Energy efficiencies (EEs) were determined by the ratio of the time-integrated output and input power density during discharging and charging over each cycle. The voltage efficiency (VE) was then determined from the EE by dividing by CE.

### Synthesis of 3-(Trifluoromethyl)-1-phenylbenzo[*e*][1,2,4]triazin-4-yl (**3a**)

In accordance with a literature procedure reported by Koutentis et al.^22^ a stirred mixture of 2,2,2-trifluoro-*N*′-phenylacetohydrazide (0.408 g, 2.00 mmol), 2-iodoaniline (0.366, 1.67 mmol), CuI (0.032 g, 0.167 mmol), and K_2_CO_3_ (0.461 g, 3.33 mmol) in degassed DMSO under a N_2_ atmosphere was heated to 90 °C for 20 h. Afterward the solution was cooled to room temperature, added to EtOAc (100 mL), and filtered to remove insoluble material. Subsequently, the organic layer was washed with H_2_O (3 × 100 mL) to remove DMSO. The aqueous layer was further back-extracted with EtOAc (2 × 50 mL), and the combined organic layers were washed with brine (200 mL), dried over MgSO_4_, filtered, and concentrated *in vacuo* to yield a dark oil. The oil was dissolved in AcOH (10 mL) and heated to 140 °C for 10 min. The mixture was allowed to cooled to room temperature and diluted with DCM (10 mL). The organic layer was washed twice with aqueous NaOH (2 M, 10 mL). The organic layer was then poured in a flask containing aqueous NaOH (2 M, 10 mL), and the biphasic mixture was stirred overnight at room temperature. The organic phase was separated again, dried over MgSO_4_, and filtered, and the volatiles were removed *in vacuo*. Chromatography of the residue on silica (DCM) yielded the 3-(trifluoromethyl)-1-phenylbenzo[*e*][1,2,4]triazin-4-yl radical **3a** as a dark red solid (0.328 g, 1.19 mmol, 71%). Anal. Calcd for C_14_H_9_F_3_N_3_: C, 60.87; H, 3.28; N, 15.21. Found: C, 60.95; H, 3.36; N, 15.22.

### Synthesis of 1-Phenyl-3-(trifluoromethyl)benzo[*e*][1,2,4]triazin-1-ium Tetrafluoroborate (**[3a^+^][BF_4_]**)

[NO][BF_4_] (48 mg, 0.41 mmol) was slowly added to a solution of **3a** (0.10 g, 0.36 mmol) in 3 mL MeCN and the mixture stirred for 2 h under inert atmosphere. The reaction mixture was filtered, and the volatiles were removed under vacuum, giving a yellowish solid. The solid was washed extensively with Et_2_O and air-dried, yielding **[3a**^**+**^**][BF**_**4**_**]** as a solid (128 mg, 0.35 mmol, 97%). ^1^H NMR (600 MHz, 25 °C, CD_3_CN): δ 8.81–8.78 (m, 2H), 8.66–8.63 (m, 1H), 8.43–8.41 (dt, 1H), 8.03–7.98 (m, 1H), 7.92–7.89 (d, 4H). ^19^F NMR (565 MHz, 25 °C, CD_3_CN): δ −75.09 (s, 3F, CF_3_), −152.0 (4F, BF_4_). ^13^C NMR (151 MHz, 25 °C, CD_3_CN): δ 154.8 (ipso-C), 153.8 (ipso-C), 145.2 (CH), 144.5 (CH), 141.8 (ipso-C), 139.5 (ipso-C), 135.4 (CH), 132.0 (CH), 131.9 (CH), 127.4 (CH), 122.5 (CH), 119.70 (d, CF_3,_^1^*J*(^19^F,^13^C) = 276 Hz).

### Synthesis of Sodium 1-Phenyl-3-(trifluoromethyl)benzo[*e*][1,2,4]triazin-4-ide (**[3a^–^][Na]**)

An excess of sodium amalgam was added to a dark red solution of **3a** in CD_3_CN in an NMR tube with J. Young valve and the tube shaken for 2 h under an inert atmosphere, during which the mixture turned orange. ^1^H NMR (600 MHz, 25 °C, CD_3_CN): δ 7.27–7.25 (m, 2H), 7.18–7.13 (mz, 2H), 6.74–6.71 (tt, 1H), 6.49–6.46 (td, 1H), 6.36–6.31 (m, 2H), 6.12–6.10 (dd, 1H); ^19^F NMR (565 MHz, 25 °C, CD_3_CN): δ −72.27. ^13^C NMR (151 MHz, 25 °C, CD_3_CN): δ 159.4 (q, ipso-C, C–CF_3_, ^2^J(^19^F, ^13^C) = 29 Hz), 150.8 (ipso-C), 148.2 (ipso-C), 136.4 (ipso-C), 129.3 (CH), 122.1 (q, CF_3_, ^1^*J*(^19^F,^13^C) = 275 Hz), 124.7 (CH), 120.5 (CH), 119.8 (CH), 118.6 (CH), 117.3 (CH), 115.0 (CH).

## Supporting Information Available

Additional experimental details and characterization data (PDF)

## Accession Codes

CCDC 2098287–2098289 contain the supplementary crystallographic data for this paper. These data can be obtained free of charge via www.ccdc.cam.ac.uk/data_request/cif, or by emailing data_request@ccdc.cam.ac.uk, or by contacting The Cambridge Crystallographic Data Centre, 12 Union Road, Cambridge CB2 1EZ, UK; fax: +44 1223 336033.
