# Supplementary material for: Blatter Radicals as Bipolar Materials for Symmetrical Redox-Flow Batteries
Source: J Am Chem Soc. 2022 Mar 8;144(11):5051–8. doi: 10.1021/jacs.1c13543 (PMC8949756; doi:10.1021/jacs.1c13543)
Supplement: Supplementary file 1 — ja1c13543_si_001.pdf [file ja1c13543_si_001.pdf]

**Supporting Information for**

# Blatter Radicals as Bipolar Materials for Symmetrical Redox-Flow Batteries


Jelte S. Steen,[a] Jules L. Nuismer,[a] Vytautas Eiva,[a] Albert E. T. Wiglema,[a] Nicolas Daub,[b] Johan Hjelm[c] and Edwin Otten*[a]

[a] Stratingh Institute for Chemistry, University of Groningen, Nijenborgh 4, 9747 AG Groningen, The Netherlands

[b] Molecular Materials and Nanosystems & Institute for Complex Molecular Systems, Eindhoven University of Technology, 5600 MB Eindhoven, The Netherlands.

[c] Department of Energy Conversion and Storage (DTU Energy), Technical University of Denmark, Fysikvej, Building 310, 2800 Kgs Lyngby, Denmark

*Corresponding author: Prof. Dr. Edwin Otten (edwin.otten@rug.nl)


## Table of Contents


General Considerations ....2

Experimentals ....4

- General Procedures ....4
- Synthesis of arylacetohydrazides **4a-4h** ....4
- Synthesis of 1,2,4-benzotriazin-4-yl radicals **3a-3h** ....6
- Synthesis of [**3a**$^+$][$BF_4$] and [**3a**$^-$][Na] ....8

NMR Spectroscopy ....9

UV-VIS Spectroscopy ....24

EPR Spectroscopy ....31

X-ray Crystallography ....34

Cyclic Voltammetry ....36

H-Cell Battery Tests ....40

Redox Flow Battery Testing ....47

Maximum Solubility Determination by UV-VIS Spectroscopy ....48

Determination of Diffusion Coefficients and Standard Rate Constants by CV ....49

References ....52

## General Considerations

All manipulations, except for arylacetohydrazide synthesis, were carried out under nitrogen atmosphere using glovebox, Schlenk and vacuum-line techniques. Glassware was dried before use at 150 °C. Radicals **1**[1], **2a**[2] and **2b**[2] were synthesized via literature procedures. The reagents used for the synthesis of arylacetohydrazide **4a-4h**, radicals **1**, **2a**, **2b**, **3a-3h** and [**3a**$^+$][$BF_4$] and [**3a**$^-$][Na] were used as received; phenylhydrazine (Sigma-Aldrich, 97%), 4-methoxyphenylhydrazine hydrochloride (TCI, >98%), 4-fluorophenylhydrazine hydrochloride (TCI, 97%), 4-trifluoromethylphenyl hydrazine (Sigma-Aldrich, 96%), 4-nitrophenylhydrazine hydrochloride (Fluorochem, 95%), 4-hydrazinylbenzoic acid hydrochloride (Acros Organics, 97%), 2,4-dichlorophenylhydrazine hydrochloride (Fluorochem, 98%), methyl trifluoroacetate (Sigma-Aldrich, 99%), 2-iodoaniline (TCI, >98%), acetic acid (Acros Organics , 99.5%), aniline (Sigma-Aldrich, 99%), benzoyl chloride (Sigma-Aldrich, 99%), Nitron (TCI, >98%), Carbon tetrachloride (Acros Organics, 99%), triphenylphosphine (Sigma-Aldrich, 99%), Palladium on Carbon 10% (Acros Organics), triethylamine (Sigma-Aldrich, 99%), 1,8-diazabicyclo[5.4.0]undec-7-ene (Sigma-Aldrich, 98%), Copper iodide (Sigma-Aldrich, 98%), potassium carbonate (Sigma-Aldrich, 99%), [NO][$BF_4$] (Alfa Aesar, 98%), Sodium mercury amalgam (Acros Organics, ca 5% sodium). Acetonitrile (Sigma-Aldrich, anhydrous, %) and acetonitrile-$d_3$ (Eurisotop) were dried over molecular sieves (Sigma-Aldrich, 3 Å), degassed by freeze-pump-thaw cycling and stored under nitrogen atmosphere. Reactions were monitored using thin layer chromatography and visualized by UV-VIS or phosphomolybdic acid stain. TLC plates (Merck) contained a silica gel matrix supported on aluminum with fluorescent indicator (254 nm). Column chromatography was performed using technical grade silica gel (Merck, pore size 60 Å and 40-63 µm particle size). Daramic® porous separators were obtained from Daramic LLC (Owensboro KY).

NMR spectra were recorded on Varian Mercury Plus 400 or Bruker Avance Neo 600. The $^1$H and $^{13}$C NMR spectra were referenced internally using the residual solvent resonances and reported in ppm relative to TMS (0 ppm). All electrochemical measurements were performed at ambient temperatures under an inert nitrogen atmosphere in acetonitrile, containing 0.1 M [$Bu_4N$][$PF_6$] as the supporting electrolyte. Electrochemical measurements were performed using a CH Instruments Electrochemical Analyzer potentiostat CHI600C and data was recorded with CHI600c software, applying *i*R compensation. Cyclic voltammetry (CV) was performed using a three-electrode configuration comprising of a Pt wire counter electrode, a $Ag/Ag^+$ (0.01 M $AgPF_6$ in 0.1 M [$Bu_4N$][$PF_6$]) single junction reference electrode and a GC disk working electrode (CHI104, CH Instruments, diameter = 3 mm). The GC working electrode was polished before the experiment using an alumina slurry (0.03 µm), rinsed with distilled water and subjected to brief ultrasonication to remove any adhered alumina microparticles. The CV data was referenced to ferrocene in acetonitrile. For measurements in pure $H_2O$, a SCE reference electrode was used. The redox-potentials reported in the main text are the half-wave potentials from cyclic voltammetry, $E_{1/2} = (E_{pa} + E_{pc})/2$ with $E_{pa}$ and $E_{pc}$ the anodic and cathodic peak potentials, respectively.

The determination of the diffusion coefficients was accomplished by varying the scan rate of the cyclic voltammetry measurements between 10 and 500 mV/s. By plotting the cathodic and anodic peak height currents vs. the square root of the scan rate showed a linear relationship which indicates a transport limited redox process (Figure S73). The slope of this linear relation was used in the Randles-Ševčik equation (S1) to determine the diffusion coefficients.

$$i_p = 0.4463 \cdot nFAC\sqrt{\frac{nFvD}{RT}} \qquad \text{(S1)}$$

With $i_p$ the peak current in A, $n$ the number of electrons transferred, $F$ the Faraday constant in C/mol, $A$ the area of the electrode in $cm^2$, $C$ the concentration of redox active species in $mol/cm^3$, $D$ the diffusion coefficient in $cm^2/s$, $v$ the scan rate in V/s, R the gas constant in J/(mol·K), and T the absolute temperature in K.

Heterogeneous electron transfer rates were determined following the Nicholson method.[3] Briefly, the peak separations ($\Delta E_p$) between the cathodic and anodic peaks depend on the scan rate. Thus, they were determined at various scan rates and fitted to a working curve to obtain the dimensionless parameter $\Psi$. Plotting the resulting values of $\Psi$ vs. the inverse of the scan rate (Figure S73) gave a linear relationship of which the slope is used to determine $k^0$ according to:

$$\Psi = \frac{\gamma k^0}{\sqrt{\pi n F v D / RT}} \qquad \text{(S2)}$$

where $\gamma$ = D/D' the ratio of the diffusion constants for the reduction and re-oxidation (**x**$^{0/-}$) or the ratio of the diffusion constants for the oxidation and re-reduction (**x**$^{0/+}$).

UV/Vis spectra were recorded in acetonitrile solution using a Agilent Technologies Cary 8454 spectrophotometer for the radicals **3a-3h** and an Avantes AvaSpec-2048 UV/Vis spectrophotometer (located in a glovebox under nitrogen atmosphere) for post-cycling analysis and [**3a**$^+$][$BF_4$] and [**3a**$^-$][Na]. High resolution mass spectra (HRMS) were acquired on a Thermo Scientific LTQ Orbitrap XL mass spectrometer, using Electron Spray Ionization (ESI). Battery cycling experiments were performed in a custom H-cell controlled by the VSP-300 potentiostat (Bio-Logic) and data was recorded with EC-LAB software (V11.31). Battery cycling experiment with polarity inversion were performed in a custom H-cell controlled by the BTS4000 (CT-4008T-5V6A-S1-F, Neware) and data was recorded with Neware BTS software (8.0.0). Electron paramagnetic resonance (EPR) measurements were carried out on a Bruker EMX Nano X-band (9.5 GHz) and performed at ambient conditions with a sample concentration of 1 mM in dichloromethane, which was degassed with nitrogen. EPR fitting was performed with the Easyspin toolbox for Matlab.[4]

Measurements under flow conditions were carried out using a zero-gap flow cell.[5] The oven-dried battery was assembled outside the glovebox. A combination of a graphite charge-collecting plate and two layers of a non-woven carbon paper electrode with an area of 2.55 $cm^2$ (Sigracet 29AA) was put on either side of the flow cell. A ±10% compression of the felt was achieved by the use of Gore-tex ePTFE gaskets. The two half-cells were separated by a Daramic 175 porous membrane. The gasket window provided for an exposed area of the membrane which was used as the active area of the flow cell. The cell was connected to a peristaltic pump (Cole-Parmer) by Masterflex C-flex ultra-pump tubing using a flow rate of 20 mL/min. The catholyte and anolyte reservoirs were filled with 6 mL of a solution of redox materials and electrolyte salt in the reported concentrations. Before starting the measurement, the cell was pretreated by flowing the solution through the cell for 30 minutes. Once the membrane was fully wetted as evidenced by impedance measurements, the cycling was started. Galvanostatic charge/discharge cycling was performed using currents of ±89.25 mA (±35 $mA/cm^2$) for the batteries shown in Figure 4 and Figure S71 with potential cutoffs at +1.7 V and 0 V. Potentiostatic electrochemical impedance spectroscopy (PEIS) measurements were performed at various stages of charge from 500 kHz to 50 Hz using a 10 mV sine perturbation. A polarization measurement was collected at full SOC and ranging from -12.75 to -255 mA. Energy efficiencies (EE) were determined by the ratio of the time-integrated output and input power density during discharging and charging over each cycle. The voltage efficiency (VE) is then determined from EE by dividing by CE.

## Experimentals

### General Procedures

**GP1: synthesis for substituted 2,2,2-trifluoromethyl-N'-arylacetohydrazides:**

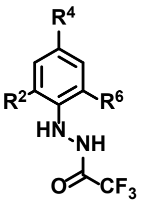


In accordance to a literature procedure by Lopyrev *et al.*[6] with slight modifications, methyl trifluoroacetate (1 eq.) was added to a 20 ml vial containing a stirring bar, substituted phenylhydrazine (1 eq.), sodium acetate (1.1 eq) and methanol. The solution was stirred overnight at room temperature. Afterwards the solution was poured in a separatory funnel containing EtOAc and $H_2O$. The aqueous layer was back extracted with EtOAc and the combined organic layers were washed with brine, dried over $MgSO_4$, filtered and the solvent was evaporated *in vacuo* to yield the substituted 2,2,2-trifluoro-N'-arylacetohydrazide as a solid.

**GP2: synthesis for substituted 3-trifluoromethyl-1-phenylbenzo[e][1,2,4]triazin-4-yl radical radicals:**

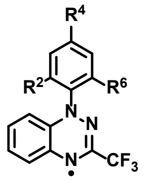


In accordance to a literature procedure reported by Koutentis *et al.*[7] a stirred mixture of substituted 2,2,2-trifluoro-N'-arylacetohydrazide (1.2 eq.), 2-iodoaniline (1 eq.), CuI (10 mol%) and $K_2CO_3$ (2 eq.) in degassed DMSO under a $N_2$ atmosphere was heated to 90 °C for 20 hours. Afterwards the solution was cooled to room temperature, added to EtOAc (100 ml) and filtered to remove insoluble material. Subsequently, the organic layer was washed with $H_2O$ (3x 100 ml) to remove DMSO. The aqueous layer was further back-extracted with EtOAc (2 x 50 ml) and the combined organic layers were washed with brine (200 ml), dried over $MgSO_4$, filtered and concentrated in vacuo to yield a dark oil. The oil was dissolved in AcOH (10 ml) and heated to 140 °C for 10 minutes. The mixture was allowed to cool to room temperature and diluted with DCM (10 ml). The organic layer was washed twice with aq. NaOH (2 M, 10 ml). The organic layer was then poured in a flask containing aq. NaOH (2M, 10 ml) and the biphasic mixture was stirred overnight at room temperature. The organic phase was separated again, dried over $MgSO_4$, filtered and the volatiles were removed *in vacuo*. Chromatography of the residue on silica (DCM) yielded the substituted 3-trifluoromethyl-1-phenylbenzo[e][1,2,4]triazin-4-yl radicals as a dark red solids.

### Synthesis of arylacetohydrazides **4a-4h**

**2,2,2-trifluoro-N'-phenylacetohydrazide (4a)**

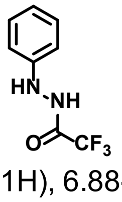


In accordance to **GP1** with methyl trifluoroacetate (0.627 g, 4.90 mmol), phenylhydrazine (0.546 g, 4.90 mmol) and sodium acetate (0.451 g, 5.50 mmol) in methanol (8 mL) afforded **4a** (0.920 g, 4.51 mmol, 92%) as a brown powder.

**$^1$H NMR** (400 MHz, 25 °C, $CDCl_3$): δ 8.11 (s, 1H), 7.30-7.28 (t, 2H), 7.02-6.98 (t, 1H), 6.88-6.84 (d, 2H), 6.05 (s, 1H); **$^{19}$F NMR** (376 MHz, 25 °C, $CDCl_3$) δ -75.09; **$^{13}$C NMR** (151 MHz, 25 °C, $CDCl_3$): δ 157.4 (q, C=O, $^2J(^{19}F, ^{13}C)$ = 38 Hz), 146.0 (*ipso*-C), 129.6 (2 x *m*-CH), 122.6 (*p*-CH), 115.9 (q, $CF_3$, $^1J(^{19}F, ^{13}C)$ = 288 Hz), 114.0 (2 x *o*-CH). The obtained spectroscopic data are in accord with those reported in the literature.[8]

**2,2,2-trifluoro-N'-(4-methoxyphenyl)acetohydrazide (4b)**

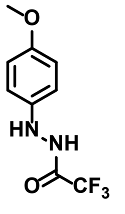

In accordance to **GP1** with methyl trifluoroacetate (0.640 g, 5.00 mmol), 4-methoxyphenylhydrazine hydrochloride (0.873 g, 5.00 mmol) and sodium acetate (0.451 g, 5.50 mmol) in methanol (8 mL) afforded **4b** (1.079 g, 4.61 mmol, 92%) as an orange brown powder.

**$^{1}$H NMR** (400 MHz, 25 °C, $CDCl_3$): δ 8.15 (s, br, 1H), 6.84 (s, 4H), 5.97 (s, br, 1H), 3.77 (s, 3H); **$^{19}$F NMR** (376 MHz, 25 °C, $CDCl_3$) δ -75.12; **$^{13}$C NMR** (151 MHz, 25 °C, $CDCl_3$): δ 157.5 (q, C=O, $^{2}J(^{19}F, ^{13}C)$ = 38 Hz), 155.7 (*ipso*-C), 115.9 (q, $CF_3$, $^{1}J(^{19}F, ^{13}C)$ = 290 Hz) 116.5 (2 x CH), 114.9 (2 x CH), 55.7 ($OCH_3$).

**2,2,2-trifluoro-N'-(4-fluorophenyl)acetohydrazide (4d)**

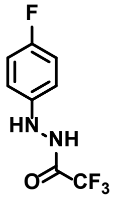

In accordance to **GP1** with methyl trifluoroacetate (0.555 g, 4.34 mmol), 4-fluorophenylhydrazine hydrochloride (0.705 g, 4.34 mmol) and sodium acetate (0.451 g, 5.50 mmol) in methanol (8 mL) afforded **4d** (0.797 g, 3.56 mmol, 82%) as a brown powder.

**$^{1}$H NMR** (400 MHz, 25 °C, $CDCl_3$): δ 8.16 (s, 1H), 7.02-6.94 (m, 2H), 6.85-6.78 (m, 2H), 6.04 (s, 1H); **$^{19}$F NMR** (376 MHz, 25 °C, $CDCl_3$) δ -75.09 (s, 3F), -121.30 (s, 1F); **$^{13}$C NMR** (151 MHz, 25 °C, $CDCl_3$): δ 158.0 (d, *p*-CF, $^{1}J(^{19}F, ^{13}C)$ = 240 Hz), 157.f8 (q, C=O, $^{2}J(^{19}F, ^{13}C)$ 7= 38 Hz), 141.9 (*ipso*-C, $^{4}J(^{19}F, ^{13}C)$ = 3 Hz), 116.2 (d, 2 x *m*-CH, $^{2}J(^{19}F, ^{13}C)$ = 24 Hz), 115.8 (q, $CF_3$, $^{1}J(^{19}F, ^{13}C)$ = 288 Hz), 115.8 (d, 2 x *o*-CH, $^{3}J(^{19}F, ^{13}C)$ = 8 Hz).

**2,2,2-trifluoro-N'-(4-trifluoromethylphenyl)acetohydrazide (4e)**

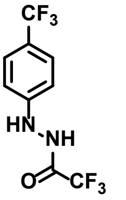

Methyl trifluoroacetate (0.384 g, 3.00 mmol) was added to a solution containing 4-trifluorophenylhydrazine (0.528 g, 3.00 mmol), triethylamine (0.404 g, 4.0 mmol) in methanol (8 mL). The solution was stirred (airtight) overnight at room temperature. Afterwards the solution was poured in a separatory funnel containing EtOAc, $H_2O$ and sat. $NH_4Cl$. The aqueous layer was back extracted with EtOAc and the combined organic layers were washed with brine, dried over $MgSO_4$, filtered and the solvent was evaporated *in vacuo* affording **4e** (0.719 g, 2.64 mmol, 88%) as an orange powder.

**$^{1}$H NMR** (400 MHz, 25 °C, $CDCl_3$): δ 8.17 (s, 1H), 7.54-7.53 (d, 2H), 6.90-6.89 (d, 2H), 6.23 (s, 1H); **$^{19}$F NMR** (376 MHz, 25 °C, $CDCl_3$) δ -59.50 (s, 3F), -73.40 (s, 3F); **$^{13}$C NMR** (151 MHz, 25 °C, $CDCl_3$): δ 157.6 (d, C=O, $^{2}J(^{19}F, ^{13}C)$ = 37 Hz), 148.9 (*ipso*-C), 127.1 (q, 2 x *m*-CH, $^{3}J(^{19}F, ^{13}C)$ = 3 Hz), 124.5 (d, *ipso*-C, $^{2}J(^{19}F, ^{13}C)$ = 33 Hz), 124.3 (d, $CF_3$, $^{1}J(^{19}F, ^{13}C)$ = 287 Hz), 115.7 (d, $CF_3$, $^{1}J(^{19}F, ^{13}C)$ = 288 Hz), 113.3 (2 x *o*-CH).

**2,2,2-trifluoro-N'-(4-nitrophenyl)acetohydrazide (4f)**

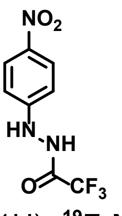

In accordance to **GP1** with methyl trifluoroacetate (0.640 g, 5.00 mmol), 4-nitrophenylhydrazine hydrochloride (0.947 g, 5.00 mmol) and sodium acetate (0.451 g, 5.50 mmol) in methanol (8 mL). Recrystallization from chloroform afforded **4f** (0.440 g, 1.77 mmol, 35%) as yellow needle-like crystals.

**$^{1}$H NMR** (400 MHz, 25 °C, $CDCl_3$): δ 8.21-8.18 (m, 3H), 6.89-6.86 (d, 2H), 6.39 (s, 1H); **$^{19}$F NMR** (376 MHz, 25 °C, $CDCl_3$) δ -74.84; **$^{13}$C NMR** (151 MHz, 25 °C, DMSO-$d_6$): δ 156.5 (q, C=O, $^{2}J(^{19}F, ^{13}C)$ = 36 Hz), 153.2 (*ipso*-C), 139.1 (*ipso*-C), 126.0 (2 x CH), 115.8 (q, $CF_3$, $^{1}J(^{19}F, ^{13}C)$ = 290 Hz), 111.0 (2 x CH).

**4-(2-(2,2,2-trifluoroacetyl)hydrazinyl)benzoic acid (4g)**

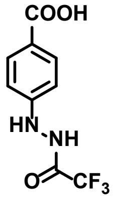


Methyl trifluoroacetate (0.640 g, 5.00 mmol) was added to a solution containing 4-hydrazinylbenzoic acid hydrochloride (0.943 g, 5.00 mmol), triethylamine (1.11 g, 11.0 mmol) in methanol (8 mL). The solution was stirred (airtight) overnight at room temperature. Azfterwards the solution was poured in a separatory funnel containing EtOAc, $H_2O$ and trifluoroacetic acid (3 mL). The aqueous layer was back extracted with EtOAc and the combined organic layers were washed with brine, dried over $MgSO_4$, filtered and the solvent was evaporated *in vacuo* affording **4g** (0.649 g, 2.6 mmol, 52%) as a yellow powder.

**$^1$H NMR** (400 MHz, 25 °C, DMSO-$d_6$): δ 11.52 (s, 1H), 8.84 (s, 1H), 7.81-7.79 (d, 2H), 6.67-6.73 (d, 2H); **$^{19}$F NMR** (376 MHz, 25 °C, DMSO-$d_6$) δ -73.70; **$^{13}$C NMR** (151 MHz, 25 °C, DMSO-$d_6$): δ 167.17 (COOH), 156.6 (q, C=O, $^2J(^{19}F, ^{13}C)$ = 35 Hz), 151.4 (*ipso*-CH), 131.1 (2 x CH), 121.3 (*ipso*-C), 116.0 (q, $CF_3$, $^1J(^{19}F, ^{13}C)$ = 288 Hz), 111.2 (2 x CH).

**N'-(2,6-dichlorophenyl)-2,2,2-trifluoroacetohydrazide (4h)**

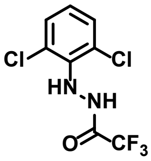


In accordance to **GP1** with methyl trifluoroacetate (0.640 g, 5.00 mmol), 2,4-dichlorophenylhydrazine hydrochloride (1.067 g, 5.00 mmol) and sodium acetate (0.451 g, 5.50 mmol) in methanol (8 mL) afforded **4h** (1.123 g, 4.1 mmol, 82%) as yellow powder.

**$^1$H NMR** (400 MHz, 25 °C, $CDCl_3$): δ 8.46 (s, 1H), 7.32-7.29 (t, 2H), 7.04-7.01 (t, 1H); 6.98 (s, 1H); **$^{19}$F NMR** (376 MHz, 25 °C, $CDCl_3$) δ -74.94; **$^{13}$C NMR** (151 MHz, 25 °C, $CDCl_3$): δ 156.3 (m, C=O, $^2J(^{19}F, ^{13}C)$ = 38 Hz), 139.3 (*ipso*-C), 129.2 (*ipso*-C), 129.2 (2 x *m*-CH), 126.6 (*p*-CH), 125.5 (2 x *o*-CCl), 115.6 (d, $CF_3$, $^1J(^{19}F, ^{13}C)$ = 299 Hz).

## Synthesis of 1,2,4-benzotriazin-4-yl radicals **3a-3h**

**1-phenyl-3-trifluoromethylbenzo[e][1,2,4]triazin-4-yl radical (3a)**

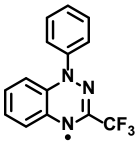


In accordance to **GP2** with **4a** (0.408 g, 2.00 mmol), 2-iodoaniline (0.366, 1.67 mmol), CuI (0.032 g, 0.167 mmol) and $K_2CO_3$ (0.461 g, 3.33 mmol) in degassed DMSO (4 mL) under a $N_2$ atmosphere at 90 °C for 20 hours. Chromatography (Silica, DCM) afforded **3a** (0.328 g, 1.19 mmol, 71%) as a dark red solid.

**HRMS** (ESI, positive mode): m/z = 276.07431 calculated for $C_{14}H_9F_3N_3$• $[M]^+$, found: 276.07430. Anal. Calcd for $C_{14}H_9F_3N_3$: C 60.87, H 3.28, N 15.21; found: C 60.95, H 3.36, N 15.22.

**1-(4-methoxyphenyl)-3-trifluoromethylbenzo[e][1,2,4]triazin-4-yl radical (3b)**

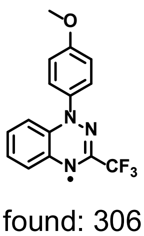


In accordance to **GP2** with **4b** (0.468 g, 2.00 mmol), 2-iodoaniline (0.366 g, 1.67 mmol), CuI (0.032 g, 0.167 mmol) and $K_2CO_3$ (0.416 g, 3.33 mmol) in degassed DMSO (4 mL) under a $N_2$ atmosphere at 90 °C for 20 hours. Chromatography (Silica, DCM) afforded **3b** (0.258 g, 0.79 mmol, 47%) as a dark red solid.

**HRMS** (ESI, positive mode): m/z = 306.08487 calculated for $C_{15}H_{11}F_3N_3O$• $[M]^+$, found: 306.08488.

**1-(4-aminophenyl)-3-trifluoromethylbenzo[e][1,2,4]triazin-4-yl radical (3c)**

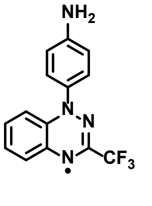

A mixture of **3f** (0.070 g, 0.218 mmol) and Pd/C (0.010 g, 0.009 mmol) in 10 mL EtOAc:EtOH (1:1) under $H_2$ atmosphere (balloon) was stirred at room temperature for 1.5 h. An immediate color change from red to yellow was observed. The reaction mixture was filtered over celite and the volatiles were removed *in vacuo* to yield a red solid. The crude product was dissolved in DCM (10 mL) and aq. NaOH (2M, 10 mL) and the biphasic mixture was stirred overnight at room temperature. The organic phase was separated, dried over $MgSO_4$, filtered and the volatiles were removed *in vacuo*. Chromatography (Silica, 3% methanol in DCM) afforded **3c** (0.063 g, 0.216 mmol, 99%) as a dark red solid.

**HRMS** (ESI, positive mode): m/z = 291.08521 calculated for $C_{14}H_{10}F_3N_4$• $[M]^+$, found: 291.08524.

**1-(4-fluorophenyl)-3-trifluoromethylbenzo[e][1,2,4]triazin-4-yl radical (3d)**

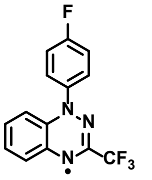

In accordance to **GP2** with **4d** (0.444 g, 2.00 mmol), 2-iodoaniline (0.366 g, 1.67 mmol), CuI (0.032 g, 0.167 mmol) and $K_2CO_3$ (0.416 g, 3.33 mmol) in degassed DMSO (4 mL) under a $N_2$ atmosphere at 90 °C for 20 hours. Chromatography (Silica, DCM) afforded **3d** (0.383 g, 1.30 mmol, 78%) as a dark red solid.

**HRMS** (ESI, positive mode): m/z = 294.06489 calculated for $C_{15}H_{11}F_3N_3O$• $[M]^+$, found: 294.06494.

**1-(4-trifluoromethylphenyl)-3-trifluoromethylbenzo[e][1,2,4]triazin-4-yl radical (3e)**

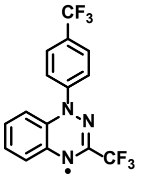

In accordance to **GP2** with **4e** (0.544 g, 2.00 mmol), 2-iodoaniline (0.366 g, 1.67 mmol), CuI (0.032 g, 0.167 mmol) and $K_2CO_3$ (0.416 g, 3.33 mmol) in degassed DMSO (4 mL) under a $N_2$ atmosphere at 90 °C for 20 hours. Chromatography (Silica, DCM) afforded **3e** (0.333 g, 0.97 mmol, 58%) as a dark red solid.

**HRMS** (ESI, positive mode): m/z = 344.06169 calculated for $C_{15}H_8F_6N_3$• $[M]^+$, found: 344.06115.

**1-(4-nitrophenyl)-3-trifluoromethylbenzo[e][1,2,4]triazin-4-yl radical (3f)**

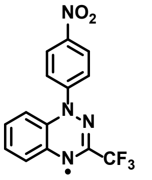

In accordance to **GP2** with **4f** (0.374 g, 1.50 mmol), 2-iodoaniline (0.274 g, 1.25 mmol), CuI (0.024 g, 0.125 mmol) and $K_2CO_3$ (0.346 g, 2.50 mmol) in degassed DMSO (4 mL) under a $N_2$ atmosphere at 90 °C for 20 hours. Chromatography (Silica, DCM) afforded **3f** (0.203 g, 0.64 mmol, 51%) as a dark red solid.

**HRMS** (ESI, positive mode): m/z = 321.0939 calculated for $C_{14}H_8F_3N_4O_2$• $[M]^+$, found: 321.05976.

**1-(4-carboxyphenyl)-3-trifluoromethylbenzo[e][1,2,4]triazin-4-yl radical (3g)**

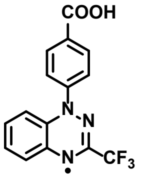

In accordance to **GP2** with **4g** (0.620 g, 2.50 mmol), 2-iodoaniline (0.458 g, 2.09 mmol), CuI (0.040 g, 0.209 mmol) and $K_2CO_3$ (0.520 g, 4.16 mmol) in degassed DMSO (4 mL) under a $N_2$ atmosphere at 90 °C for 20 hours. Afterwards, the solution was cooled to room temperature and added to EtOAc (100 ml), $H_2O$ (100 ml) and trifluoroacetic acid (4 ml) in a separatory funnel. The aqueous layer was further back-extracted with EtOAc (2 x 50 ml) and the combined organic layers were washed with brine (200 ml), filtered and the volatiles were evaporated *in vacuo* to yield a dark red oil. The oil was dissolved in AcOH (10 ml) and heated to 140 °C for 10 minutes. The mixture was allowed to cool to room temperature and diluted with DCM (15 ml). To this solution aq. NaOH

(2M) was added until the pH > 10. The biphasic mixture was stirred overnight at room temperature. To this mixture trifluoroacetic acid (5 ml) was added until the pH < 7. The solution was washed twice with $H_2O$ (100 ml), dried over $MgSO_4$, filtered and the volatiles were removed *in vacuo*. Chromatography (Silica, 40% EtOAc in pentane -> 4% MeOH in DCM) afforded **3g** (0.128 g, 18% yield) as a dark red solid.

**HRMS** (ESI, positive mode): m/z = 321.07196 calculated for $C_{15}H_{10}F_3N_3O_2$• $[M+H]^+$, found: 321.07297.

#### 1-(2,4-dichlorophenyl)-3-trifluoromethylbenzo[e][1,2,4]triazin-4-yl radical (3h)

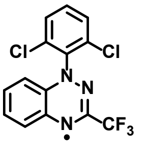


In accordance to **GP2** with **4h** (0.546 g, 1.50 mmol), 2-iodoaniline (0.366 g, 1.25 mmol), CuI (0.032 g, 0.125 mmol) and $K_2CO_3$ (0.346 g, 2.50 mmol) in degassed DMSO (4 mL) under a $N_2$ atmosphere at 90 °C for 20 hours. Chromatography (Silica, EtOAc:Pentane (15:85) -> EtOAc:Pentane (25:75)) and recrystallization from a saturated solution of DCM in hexane afforded **3h** (0.060 g, 0.17 mmol, 10%) as dark red crystals.

**HRMS** (ESI, positive mode): m/z = 345.00419 calculated for $C_{14}H_8F_3N_3Cl_2$• $[M+H]^+$, found: 345.00430.

## Synthesis of [**3a**$^+$][$BF_4$] and [**3a**$^-$][Na]

#### 1-phenyl-3-(trifluoromethyl)benzo[e][1,2,4]triazin-1-ium tetrafluoroborate ([3a$^+$][$BF_4$])

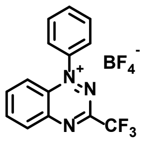


[NO][$BF_4$] (48 mg, 0.41 mmol) was slowly added to a solution of **3a** (0.10 g, 0.36 mmol) in 3 mL $CH_3CN$ and stirred for 2 hours under inert atmosphere. The reaction mixture was filtered and the volatiles were removed under vacuum obtaining a yellowish solid. The solid was washed extensively with $Et_2O$ and air-dried yielding [**3a**$^+$][$BF_4$] as a solid (128 mg, 0.35 mmol, 97%).

$^1$**H NMR** (600 MHz, 25 °C, $CD_3CN$): δ 8.81-8.78 (m, 2H), 8.66-8.63 (m, 1H), 8.43-8.41 (dt, 1H), 8.03-7.98 (m, 1H), 7.92-7.89 (d, 4H); $^{19}$**F NMR** (565 MHz, 25 °C, $CD_3CN$) δ -75.09 (s, 3F, $CF_3$), -152.0 (4F, $BF_4$); $^{13}$**C NMR** (151 MHz, 25 °C, $CD_3CN$): δ 154.8 (*ipso*-C), 153.8 (*ipso*-C), 145.2 (CH), 144.5 (CH), 141.8 (*ipso*-C), 139.5 (*ipso*-C), 135.4 (CH), 132.0 (CH), 131.9 (CH), 127.4 (CH), 122.5 (CH), 119.7 (d, $CF_3$, $^1J(^{19}F, ^{13}C)$ = 273 Hz).

#### Sodium 1-phenyl-3-(trifluoromethyl)-benzo[e][1,2,4]triazin-4-ide ([3a$^-$][Na])

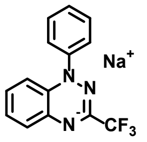


An excess of sodium amalgam was added to a dark red solution of **3a** in $CD_3CN$ in a NMR tube with J Young valve and shaken for 2 hours under inert atmosphere during which the colour turned orange.

$^1$**H NMR** (600 MHz, 25 °C, $CD_3CN$): δ 7.27-7.25 (m, 2H), 7.18-7.13 (mz, 2H), 6.74-6.71 (tt, 1H), 6.49-6.46 (td, 1H), 6.36-6.31 (m, 2H), 6.12-6.10 (dd, 1H); $^{19}$**F NMR** (565 MHz, 25 °C, $CD_3CN$) δ -72.27; $^{13}$**C NMR** (151 MHz, 25 °C, $CD_3CN$): δ 159.4 (q, *ipso*-C, *C*-$CF_3$, $^2J(^{19}F, ^{13}C)$ = 29 Hz), 150.8 (*ipso*-C), 148.2 (*ipso*-C), 136.4 (*ipso*-C), 129.3 (CH), 122.1 (q, $CF_3$, $^1J(^{19}F, ^{13}C)$ = 275 Hz), 124.7 (CH), 120.5 (CH), 119.8 (CH), 118.6 (CH), 117.3 (CH), 115.0 (CH).

# NMR Spectroscopy

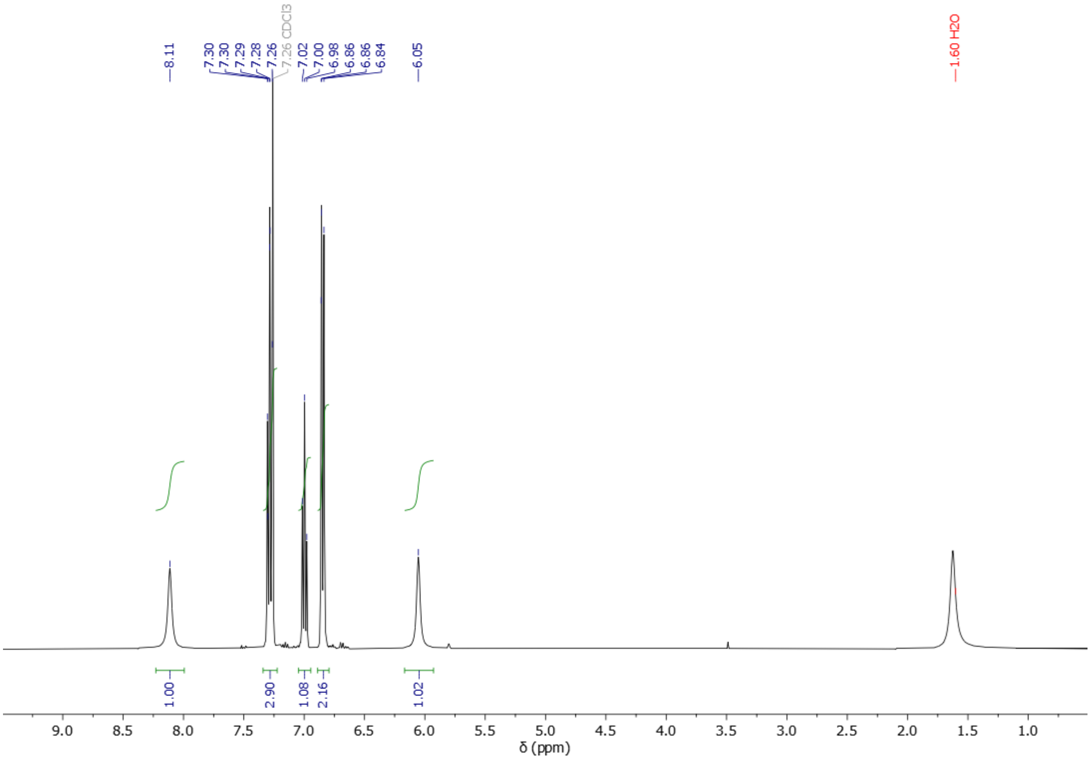


**Figure S1:** $^{1}H$ NMR spectrum of **4a** ($CDCl_3$, 25 °C, 400 MHz).

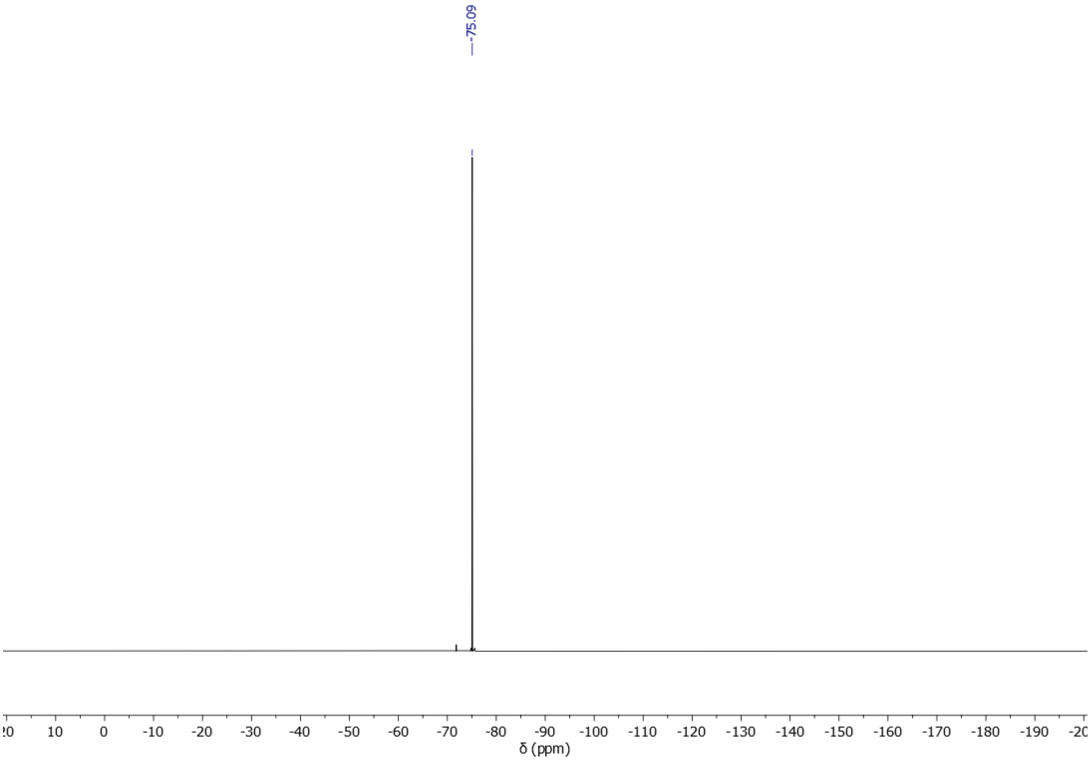


**Figure S2:** $^{19}F$ NMR spectrum of **4a** ($CDCl_3$, 25 °C, 376 MHz).

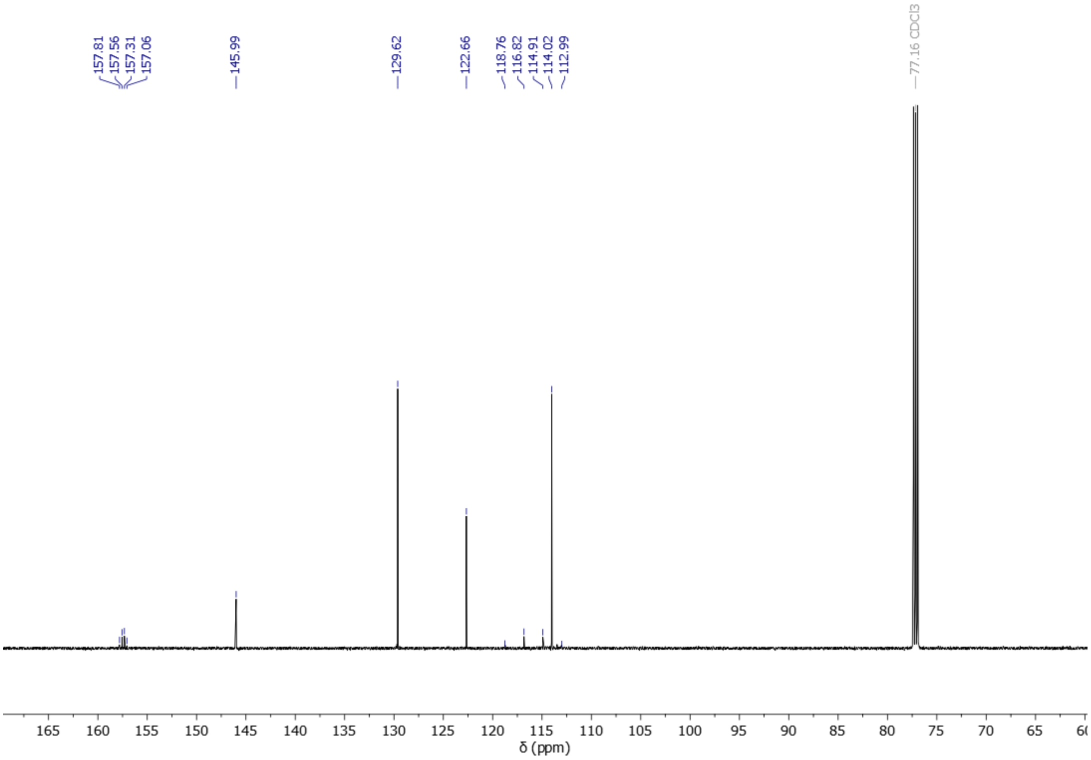


**Figure S3:** $^{13}C$ NMR spectrum of **4a** ($CDCl_3$, 25 °C, 151 MHz).

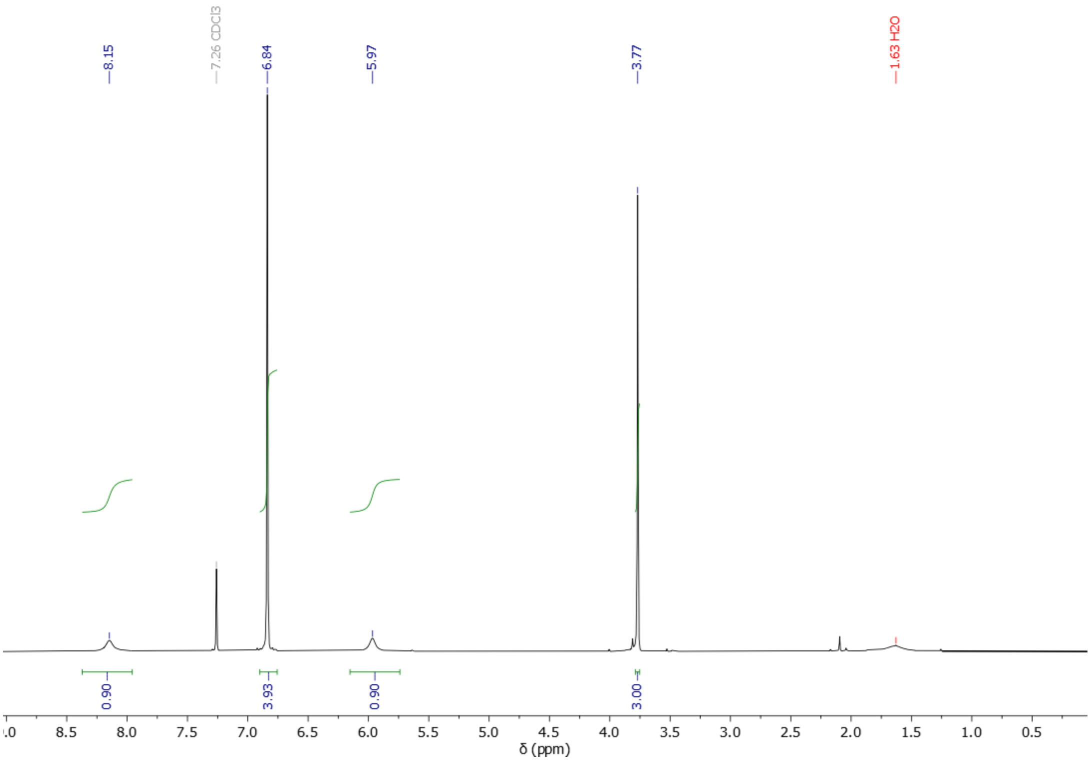


**Figure S4:** $^{1}H$ NMR spectrum of **4b** ($CDCl_3$, 25 °C, 400 MHz).

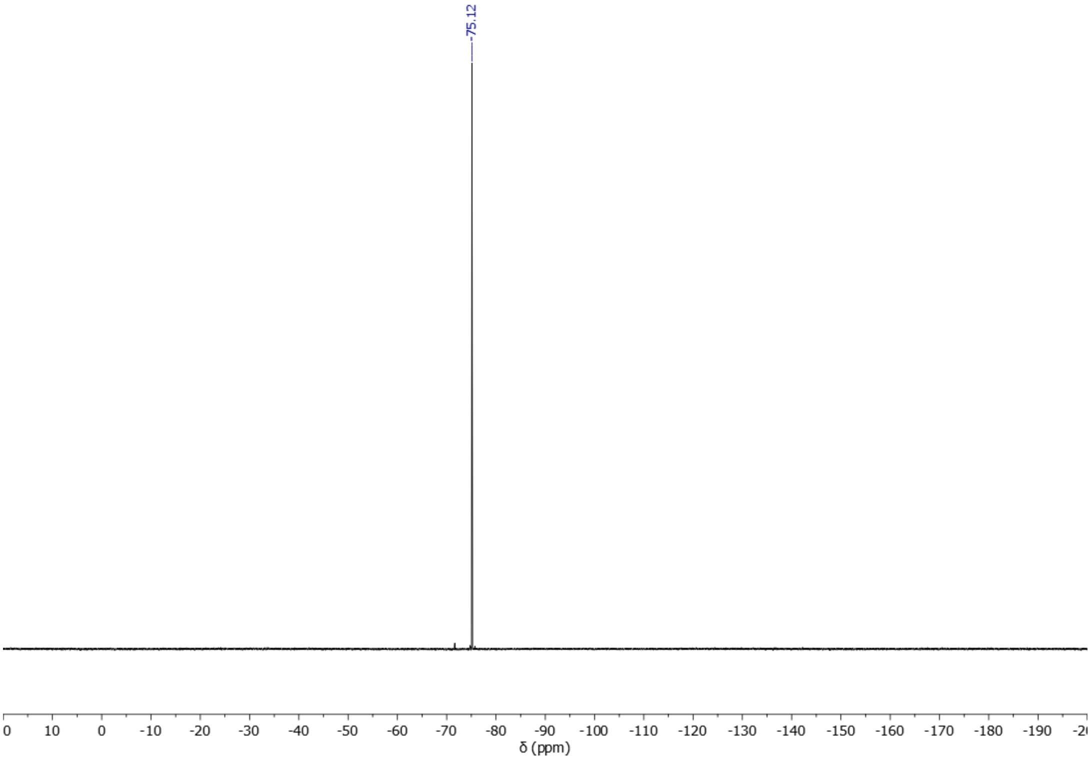


**Figure S5:** $^{19}$F NMR spectrum of **4b** ($CDCl_3$, 25 °C, 376 MHz).

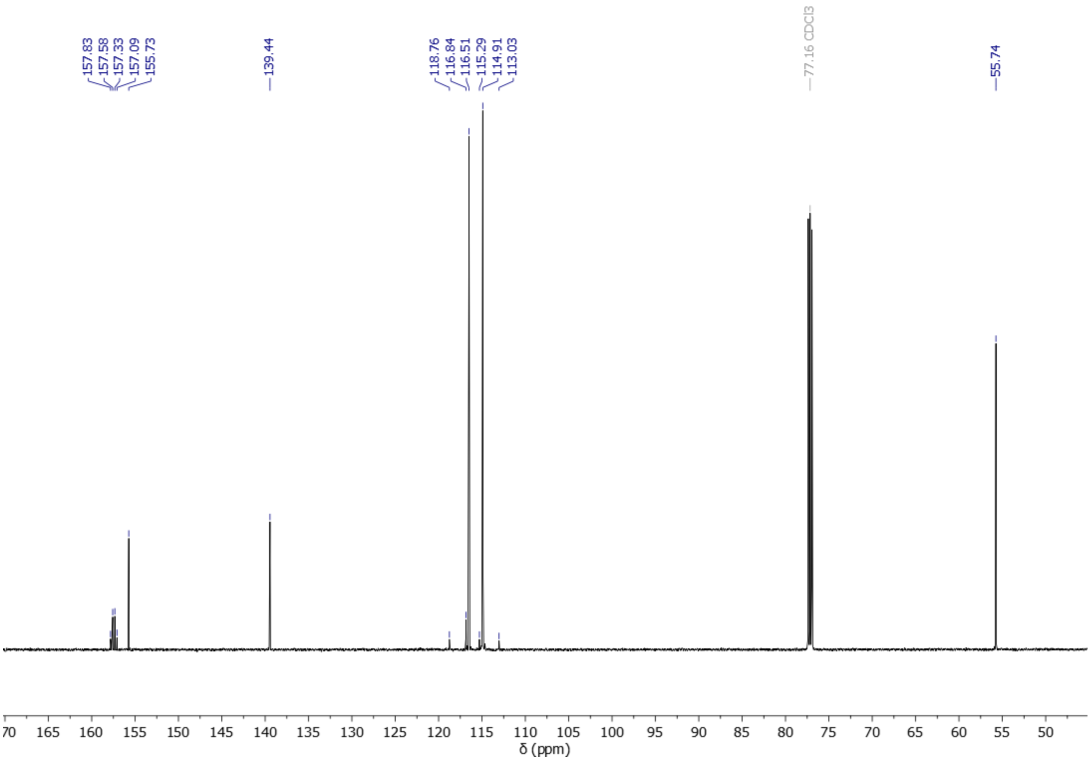


**Figure S6:** $^{13}$C NMR spectrum of **4b** ($CDCl_3$, 25 °C, 151 MHz).

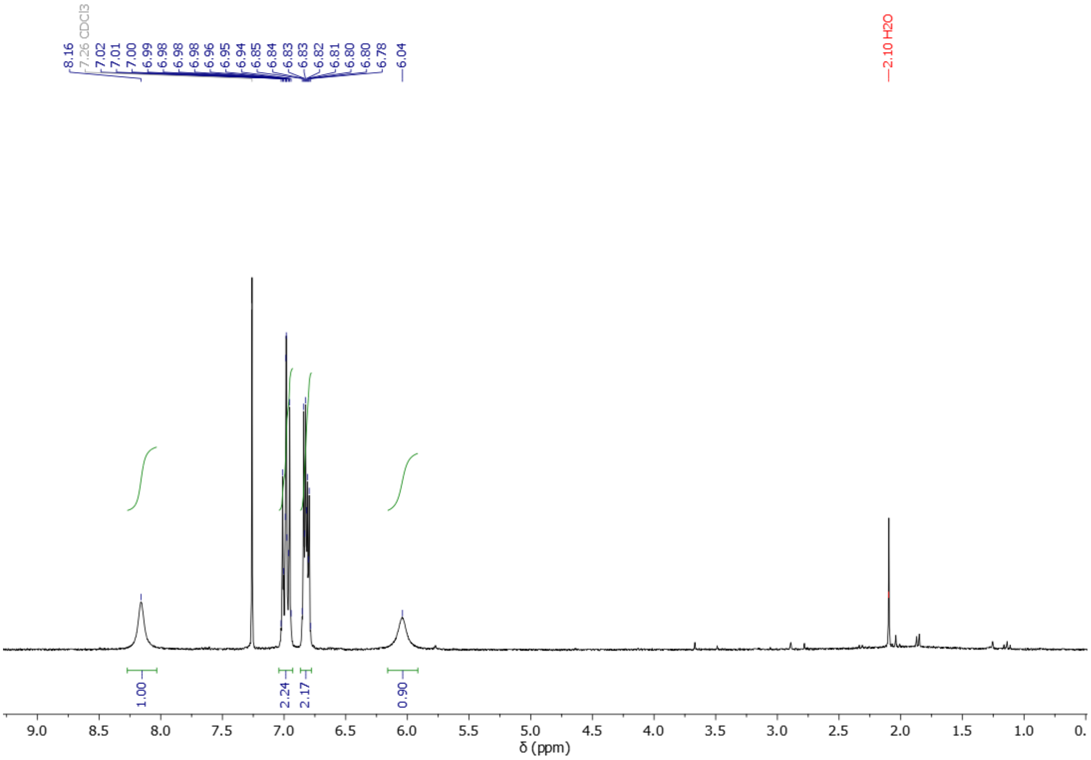


**Figure S7:** $^{1}H$ NMR spectrum of **4d** ($CDCl_3$, 25 °C, 400 MHz).

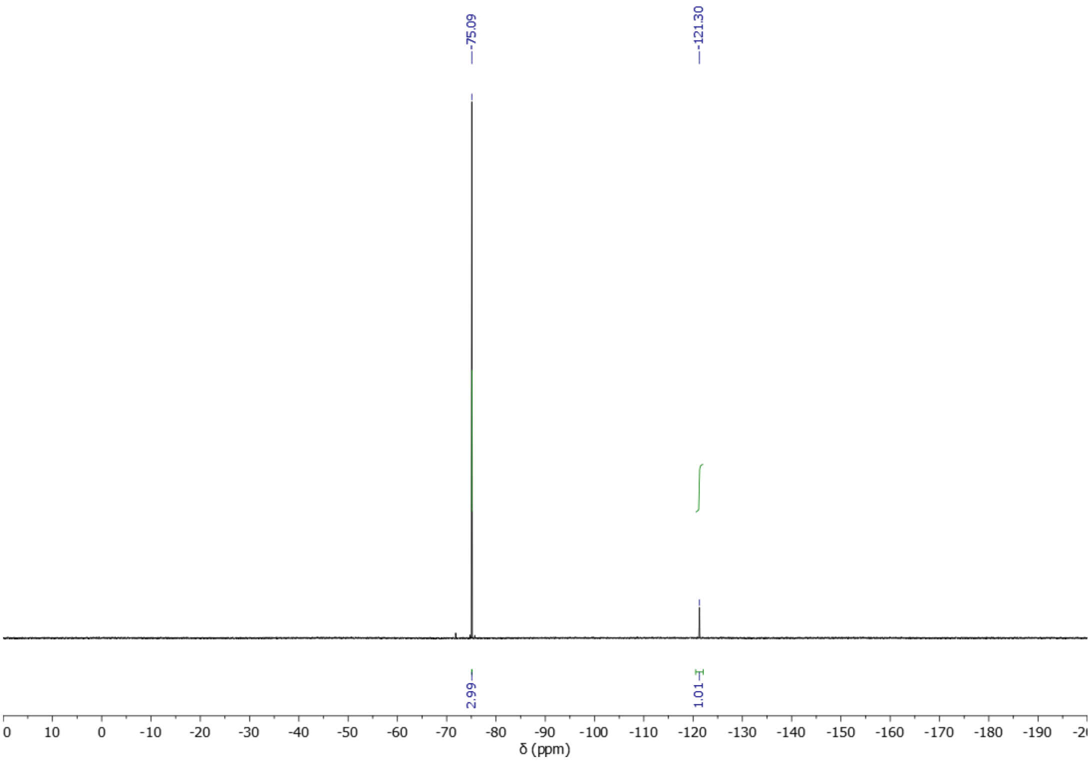


**Figure S8:** $^{19}F$ NMR spectrum of **4d** ($CDCl_3$, 25 °C, 376 MHz).

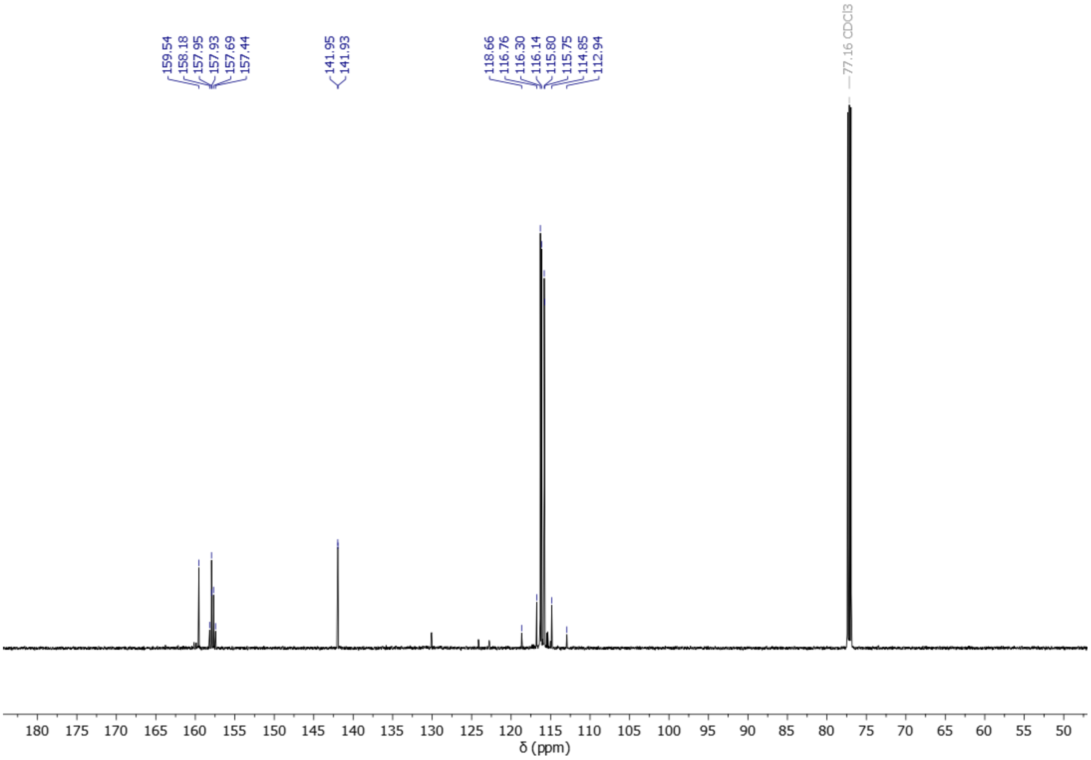


**Figure S9:** $^{13}$C NMR spectrum of **4d** ($CDCl_3$, 25 °C, 151 MHz).

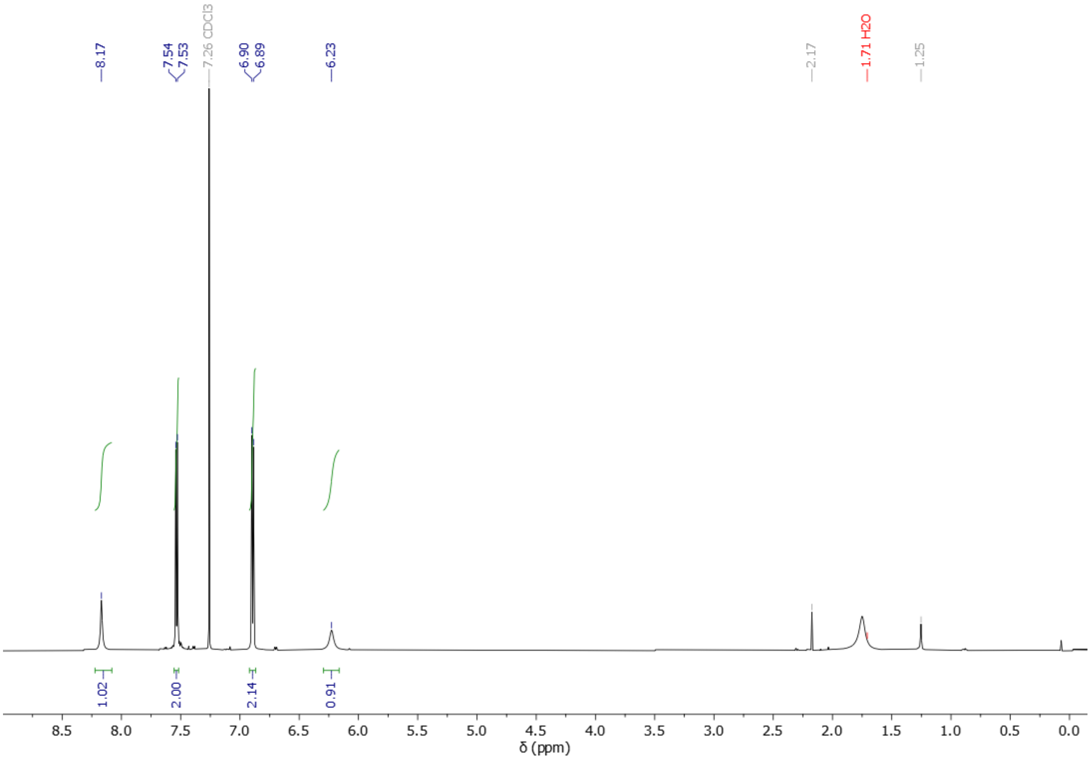


**Figure S10:** $^{1}$H NMR spectrum of **4e** ($CDCl_3$, 25 °C, 400 MHz).

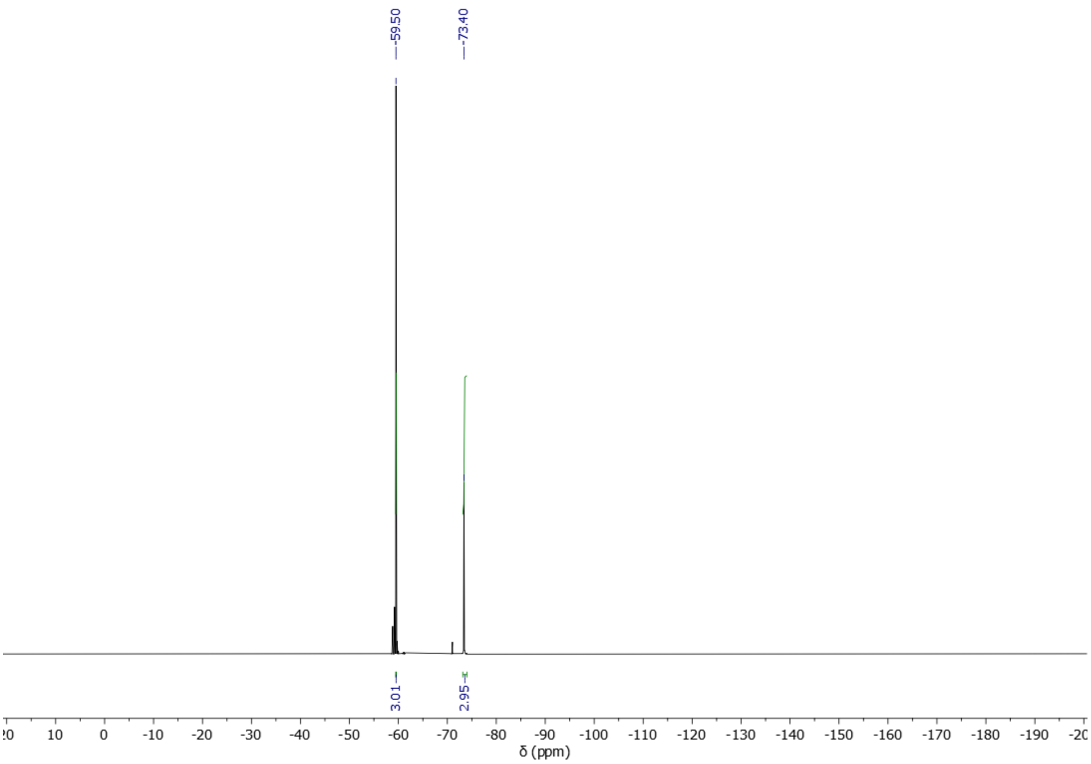


**Figure S11:** $^{19}$F NMR spectrum of **4e** ($CDCl_3$, 25 °C, 376 MHz).

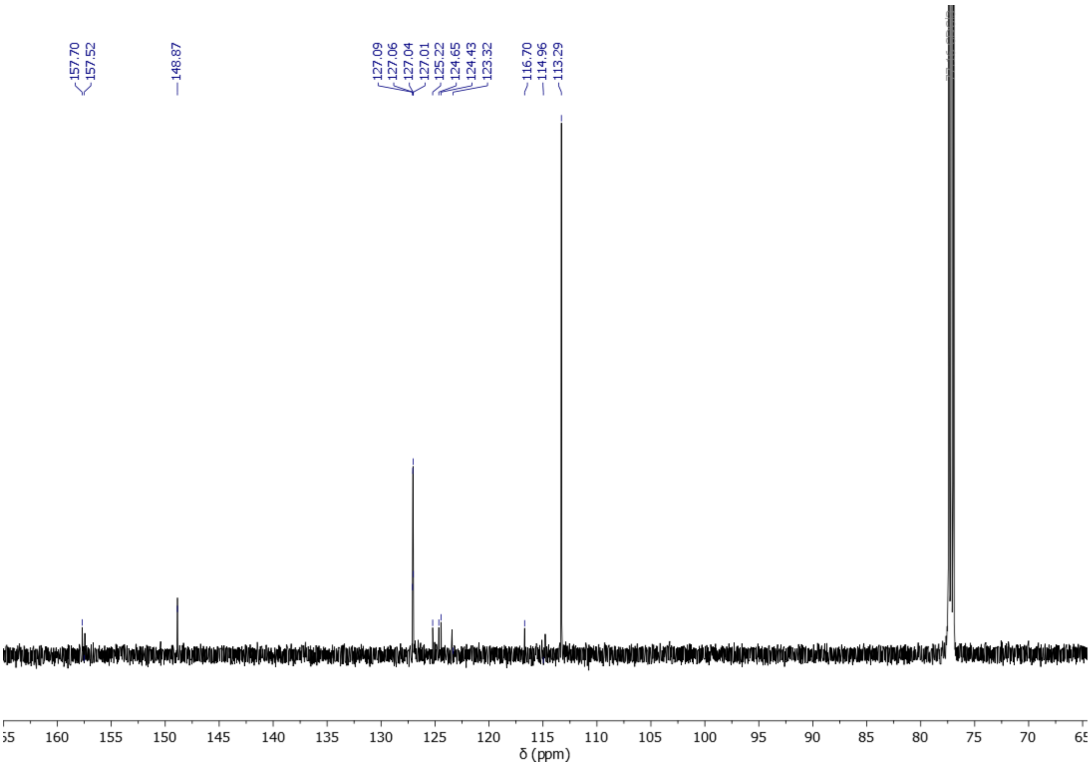


**Figure S12:** $^{13}$C NMR spectrum of **4e** ($CDCl_3$, 25 °C, 151 MHz).

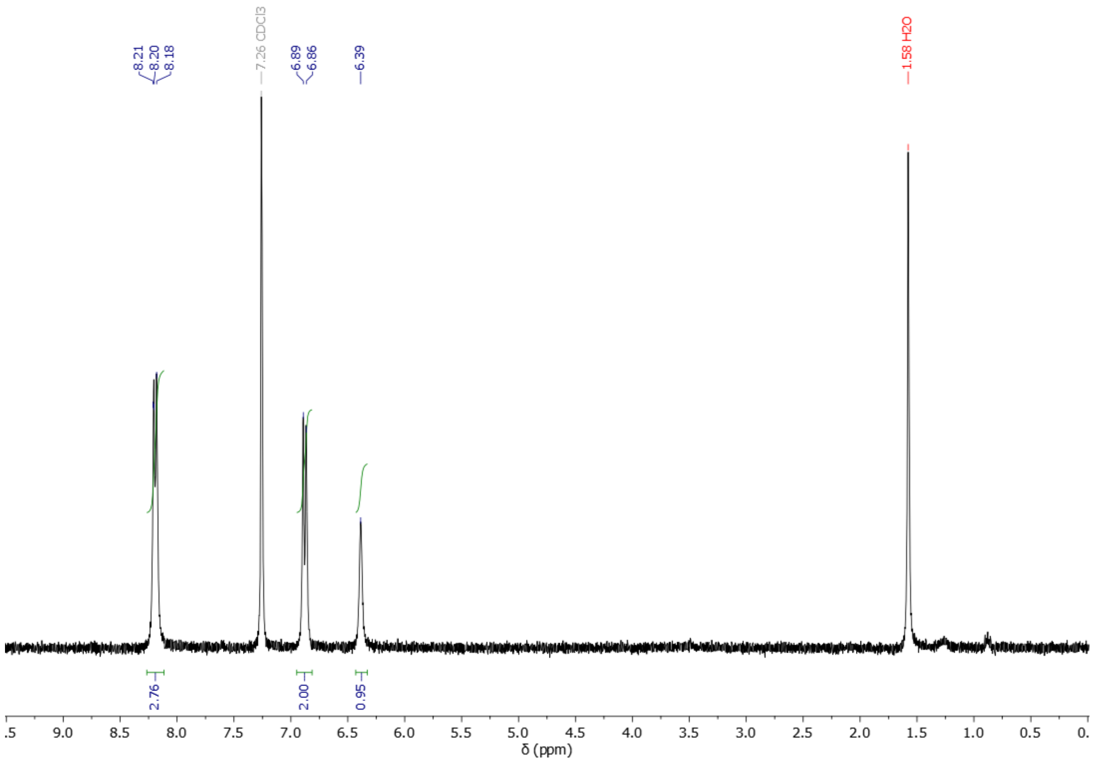


**Figure S13:** $^{1}$H NMR spectrum of **4f** ($CDCl_3$, 25 °C, 400 MHz).

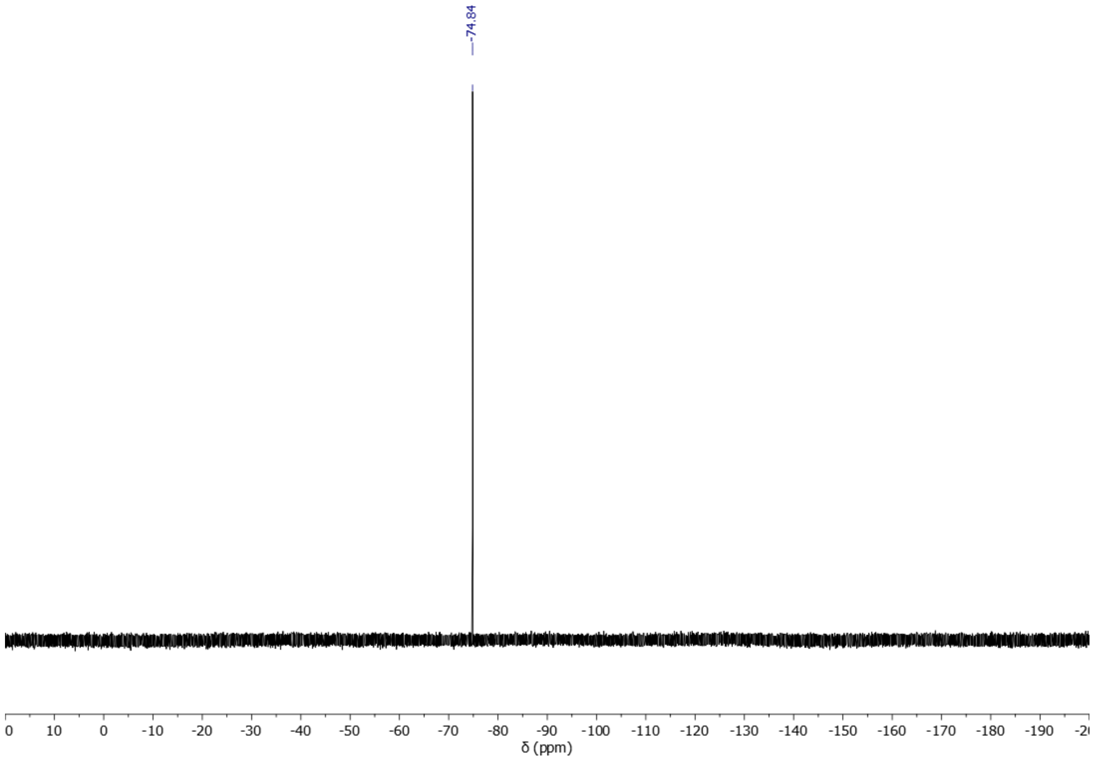


**Figure S14:** $^{19}$F NMR spectrum of **4f** ($CDCl_3$, 25 °C, 376 MHz).

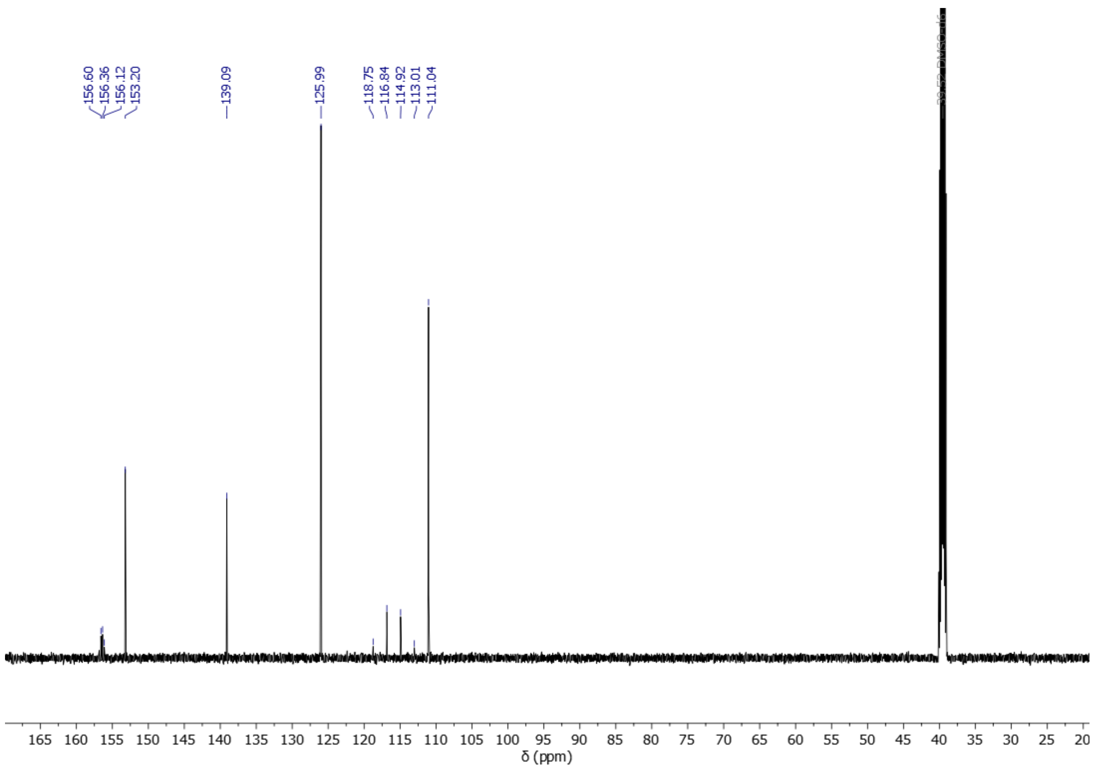


**Figure S15:** $^{13}C$ NMR spectrum of **4f** (DMSO-d$_6$, 25 °C, 151 MHz).

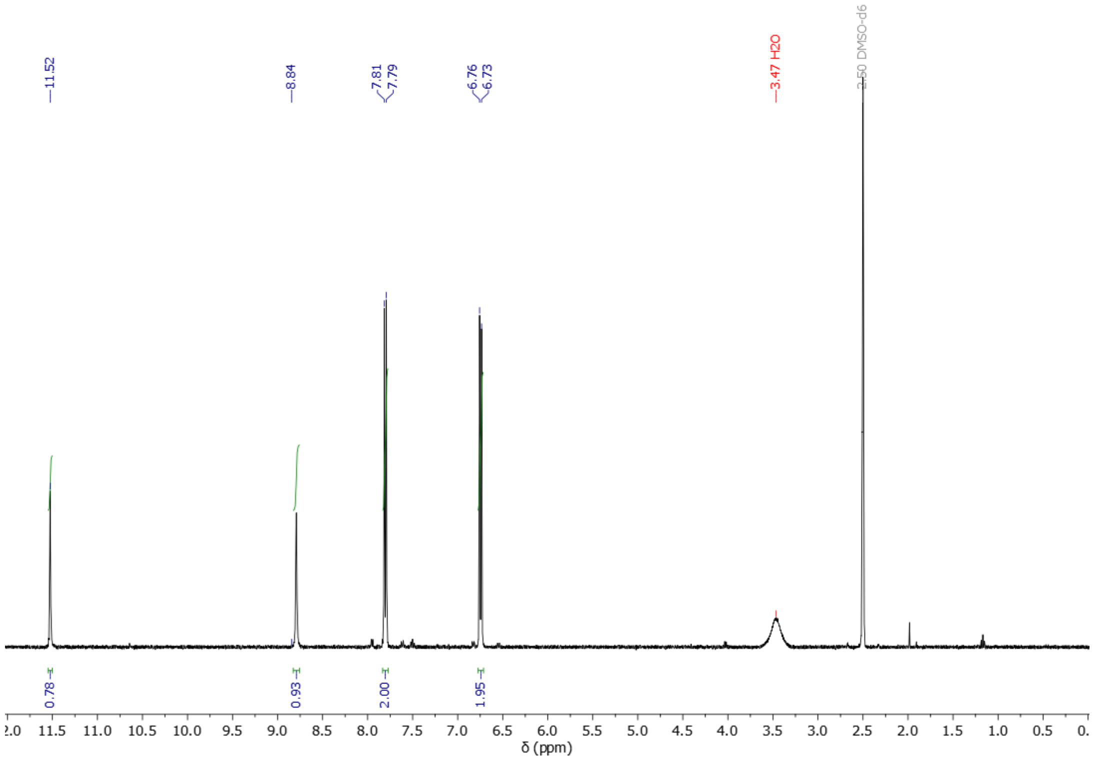


**Figure S16:** $^{1}H$ NMR spectrum of **4g** (DMSO-d$_6$, 25 °C, 400 MHz).

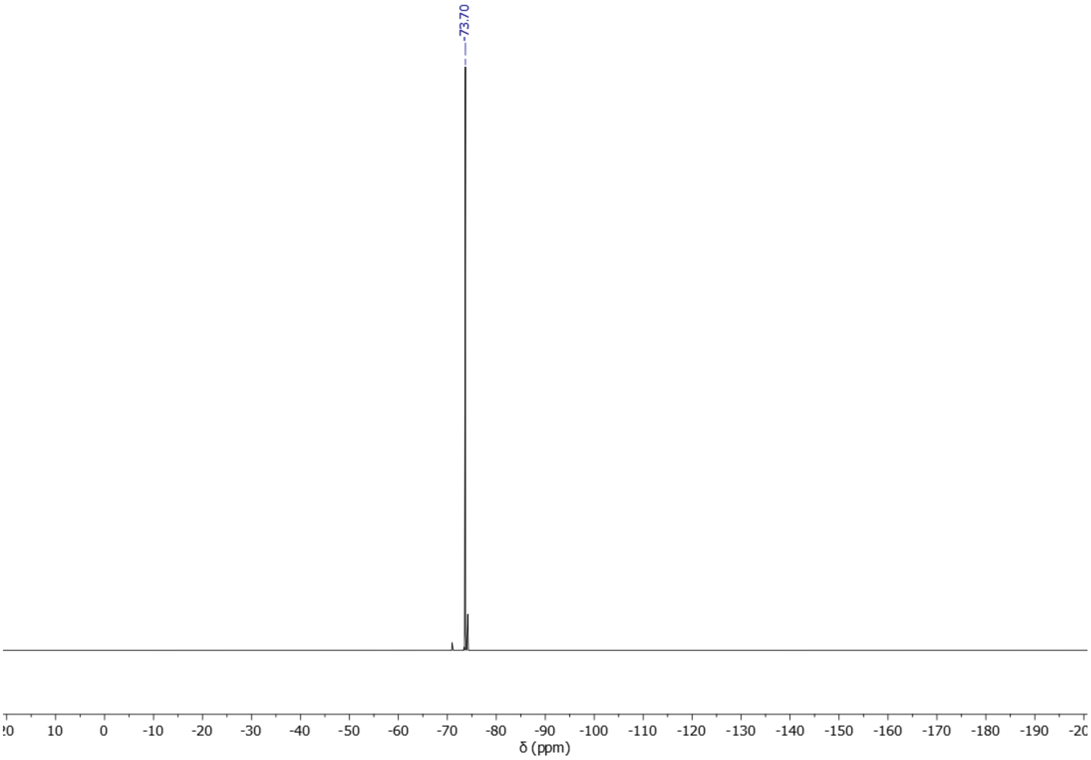


**Figure S17:** $^{19}$F NMR spectrum of **4g** (DMSO-$d_6$, 25 °C, 376 MHz).

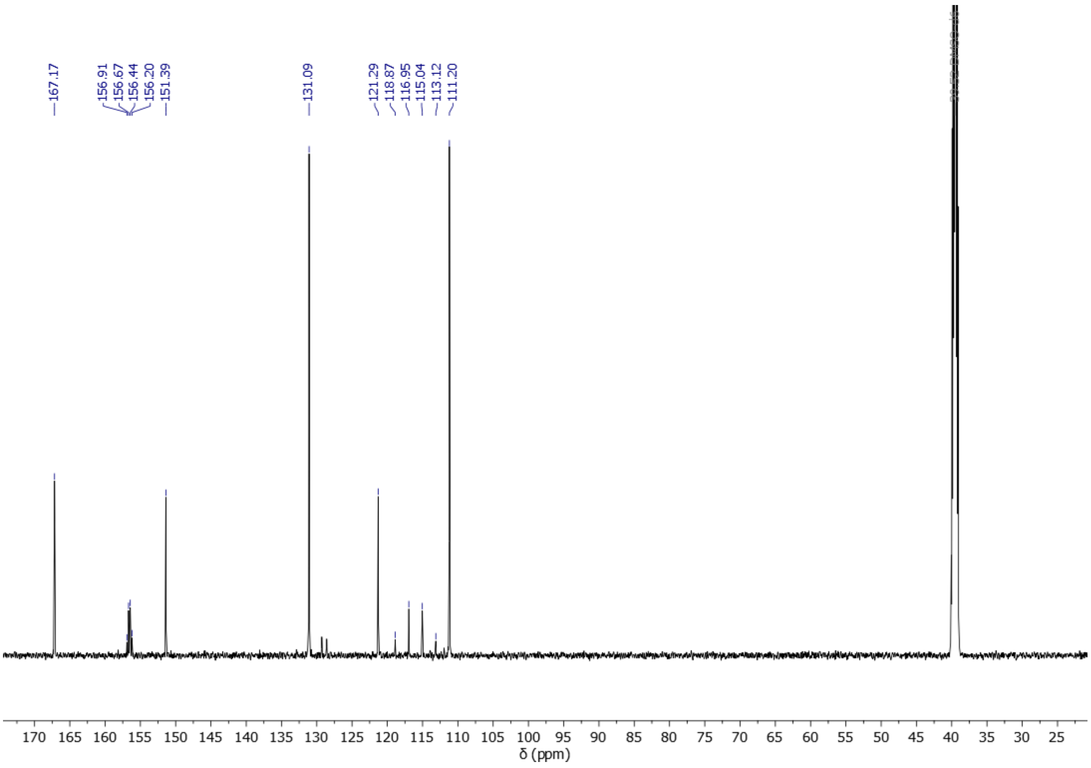


**Figure S18:** $^{13}$C NMR spectrum of **4g** (DMSO-$d_6$, 25 °C, 151 MHz).

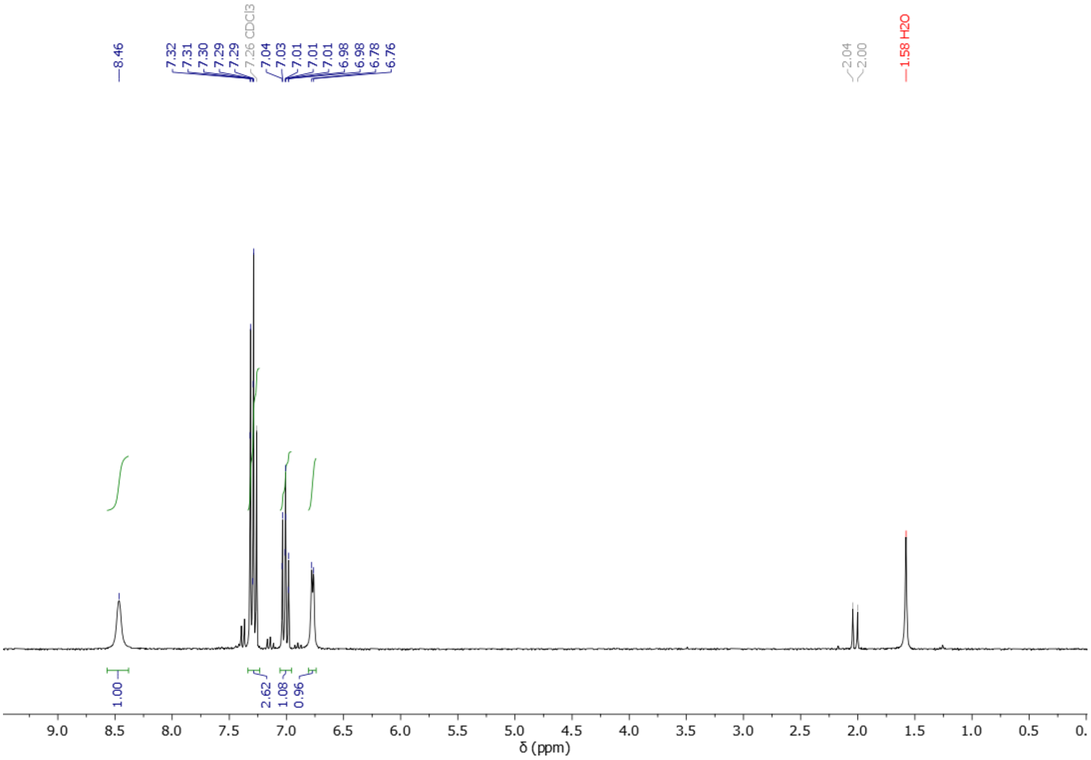


**Figure S19:** $^{1}$H NMR spectrum of **4h** ($CDCl_3$, 25 °C, 400 MHz).

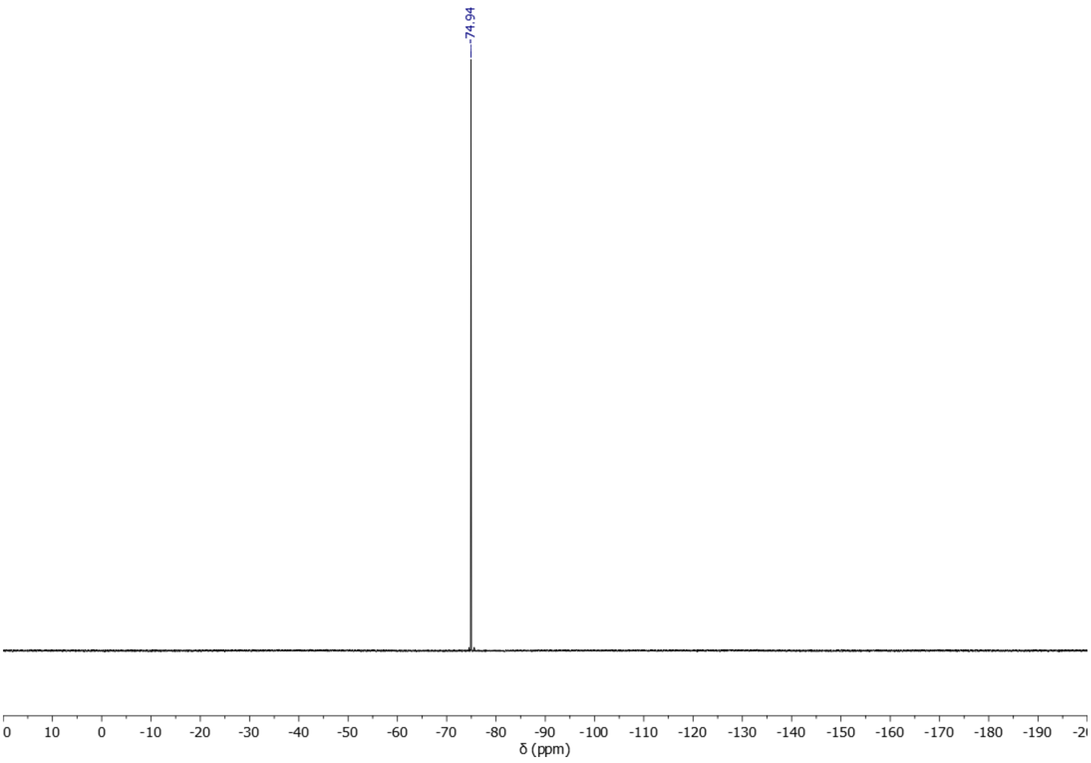


**Figure S20:** $^{19}$F NMR spectrum of **4h** ($CDCl_3$, 25 °C, 376 MHz).

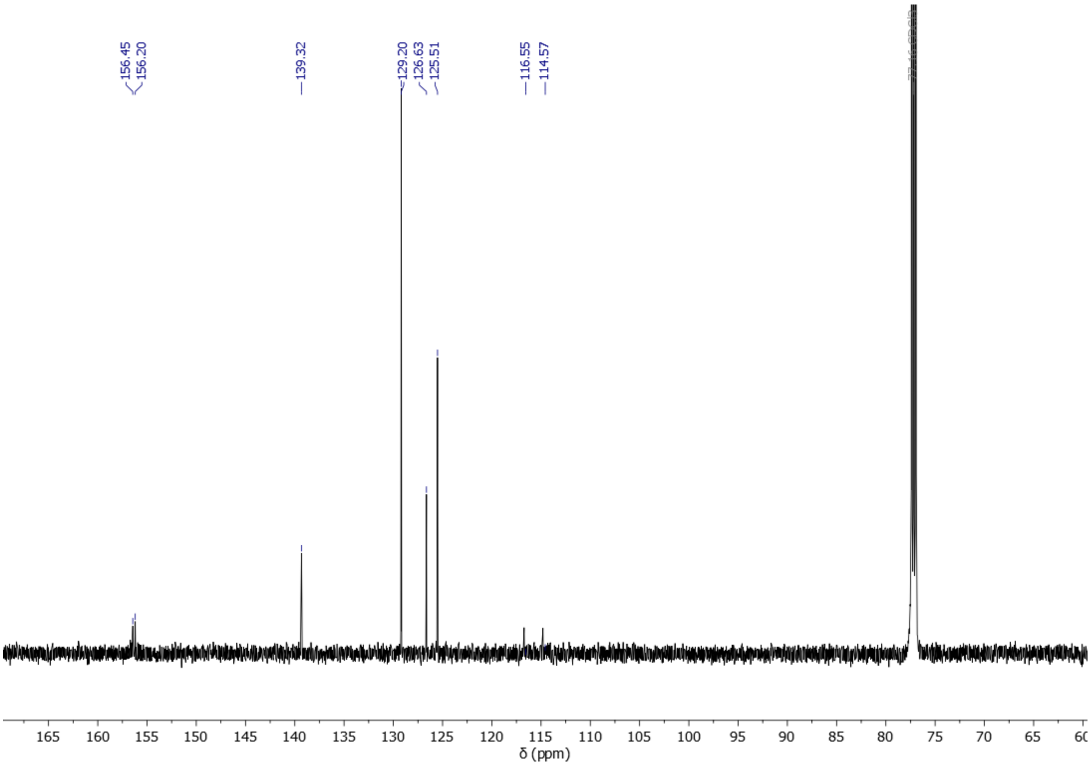


**Figure S21:** $^{13}$C NMR spectrum of **4h** ($CDCl_3$, 25 °C, 151 MHz).

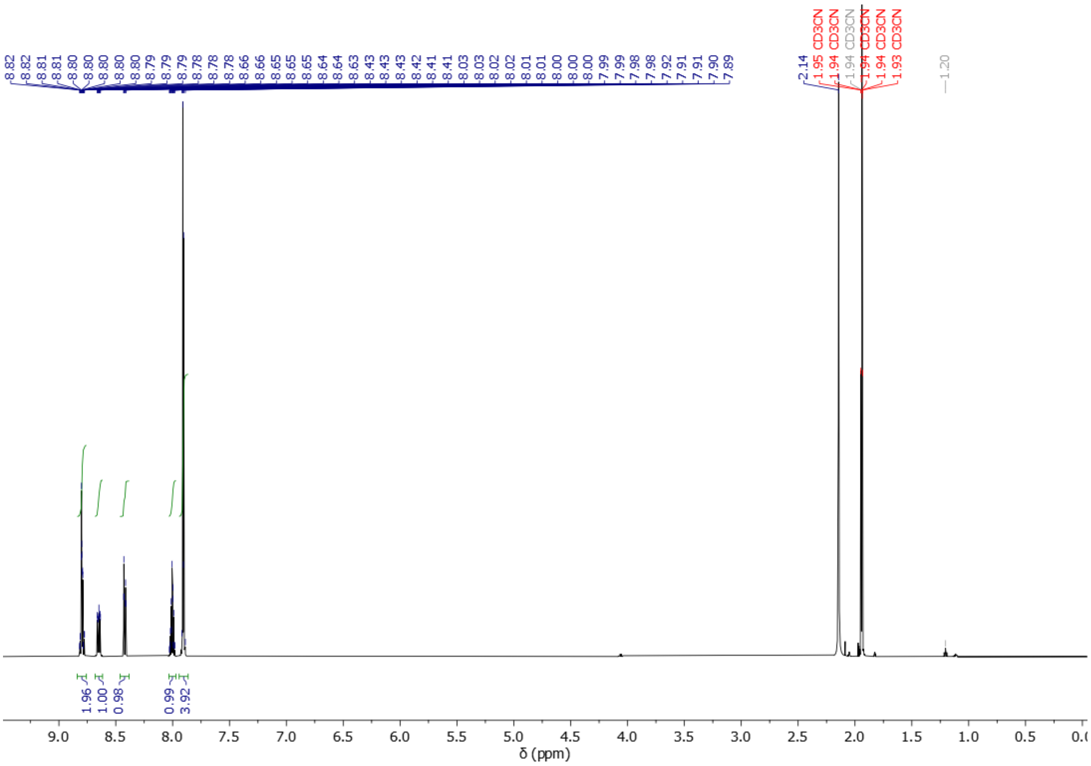


**Figure S22:** $^{1}$H NMR spectrum of [**3a**$^{+}$][$BF_4^{-}$] ($CD_3CN$, 25 °C, 600 MHz).

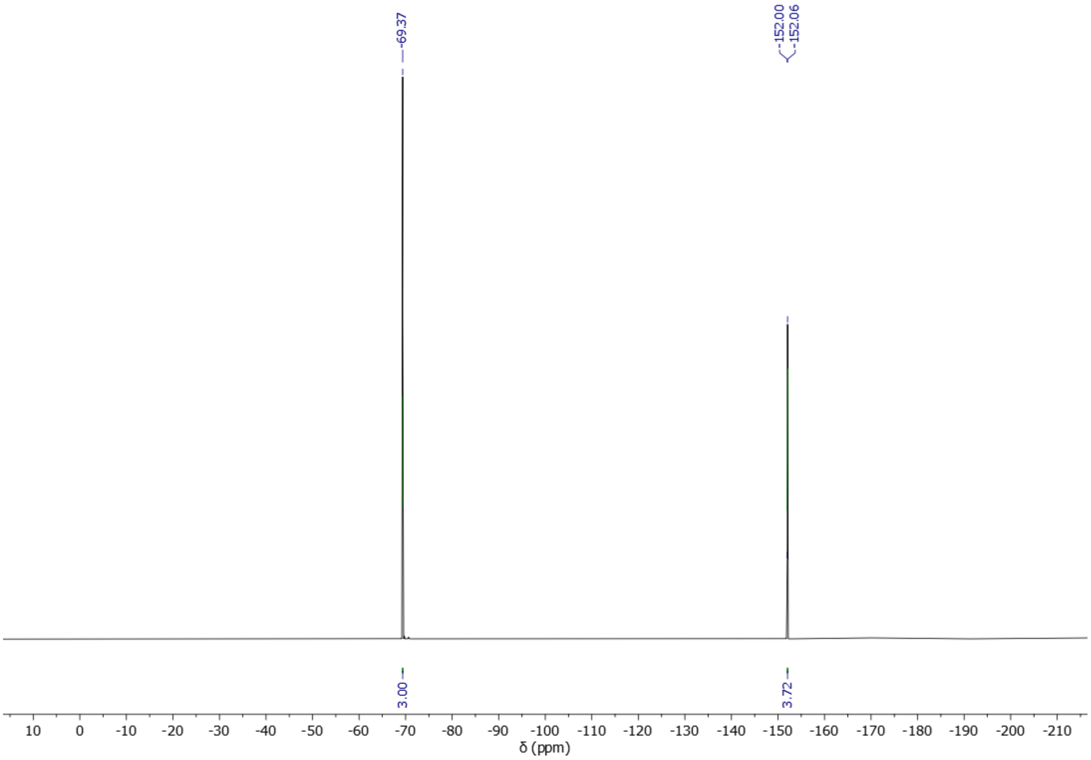


**Figure S23:** $^{19}$F NMR spectrum of [**3a**$^{+}$][$BF_4^{-}$] ($CD_3CN$, 25 °C, 564 MHz).

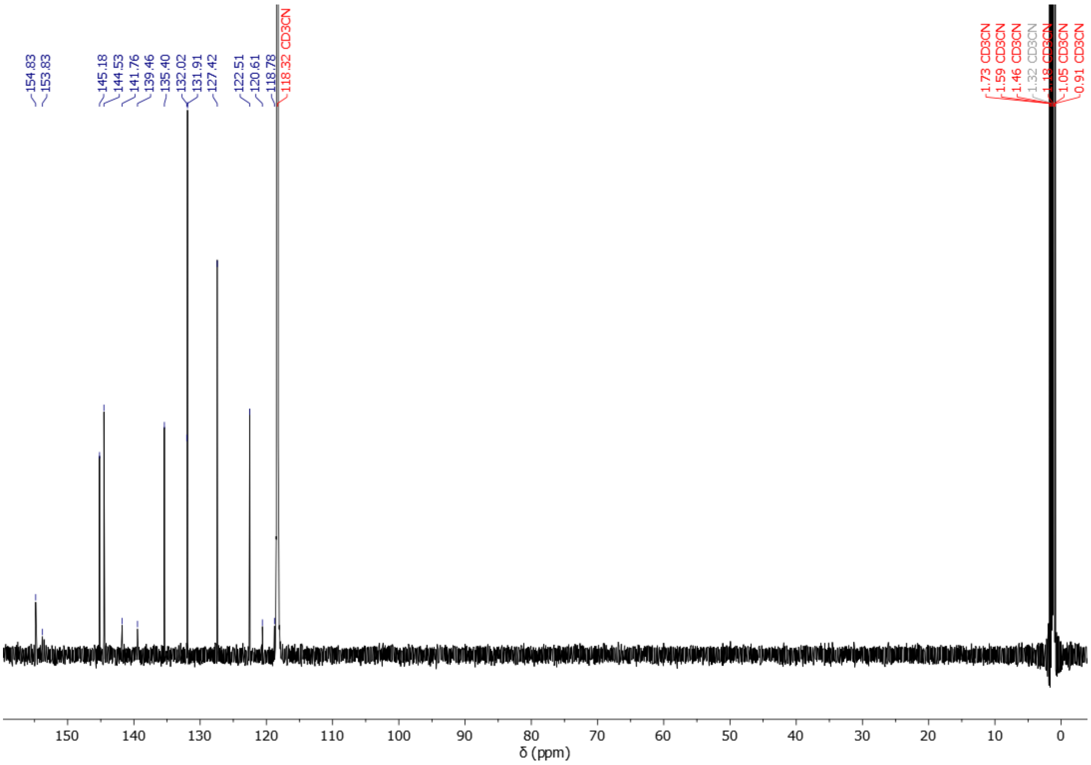


**Figure S24:** $^{13}$C NMR spectrum of [**3a**$^{+}$][$BF_4^{-}$] ($CD_3CN$, 25 °C, 151 MHz).

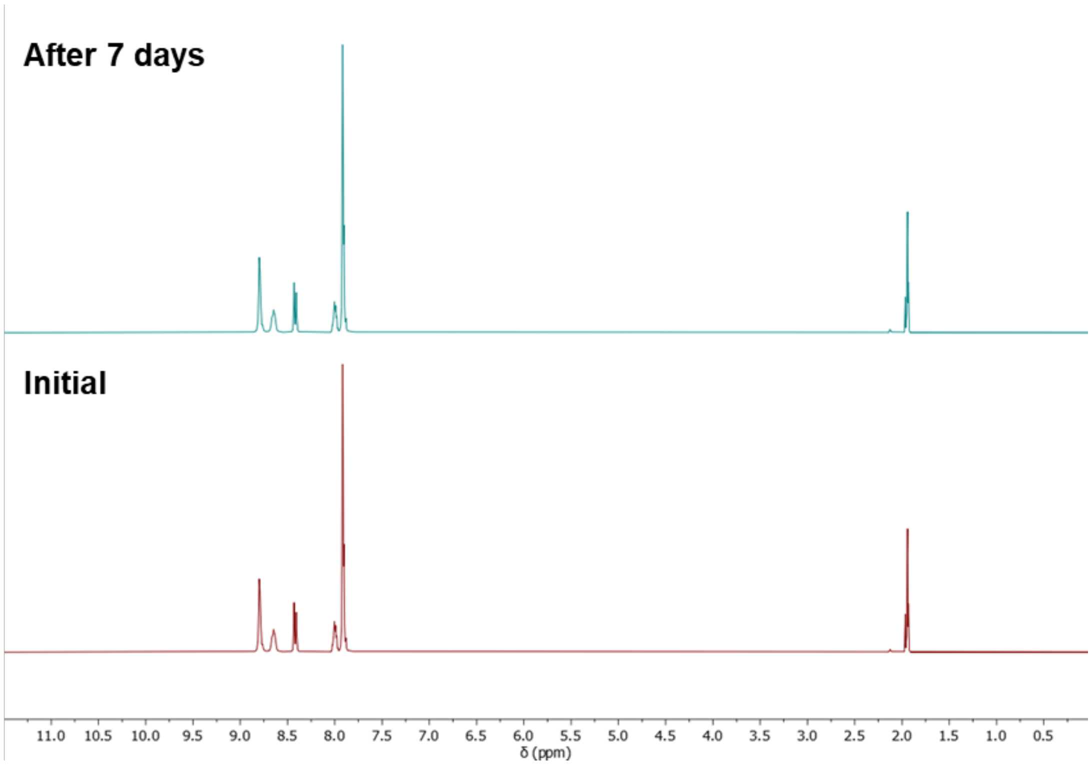


**Figure S25:** Comparison of $^{1}H$ NMR spectra of [**3a**$^{+}$][$BF_4^{-}$] showing no sign of decomposition after seven days in a Teflon-sealed NMR tube under $N_2$ atmosphere ($CD_3CN$, 25 °C, 400 MHz).

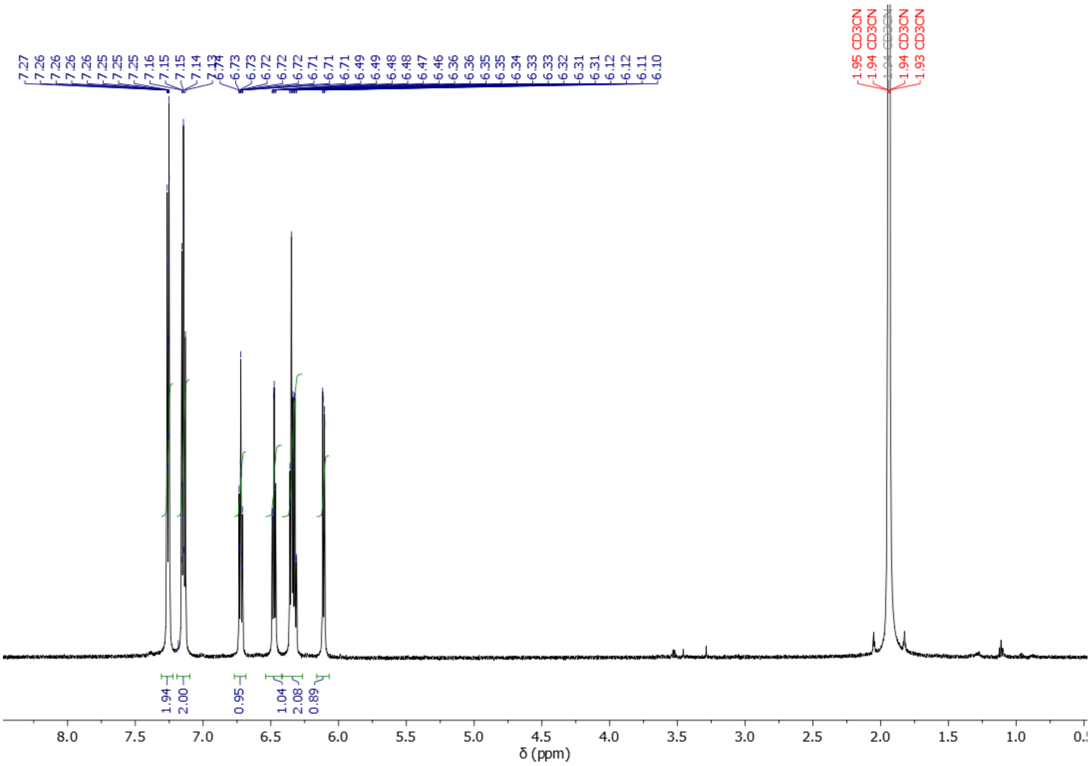


**Figure S26:** $^{1}H$ NMR spectrum of [**3a**$^{-}$][$Na^{+}$] ($CD_3CN$, 25 °C, 600 MHz).

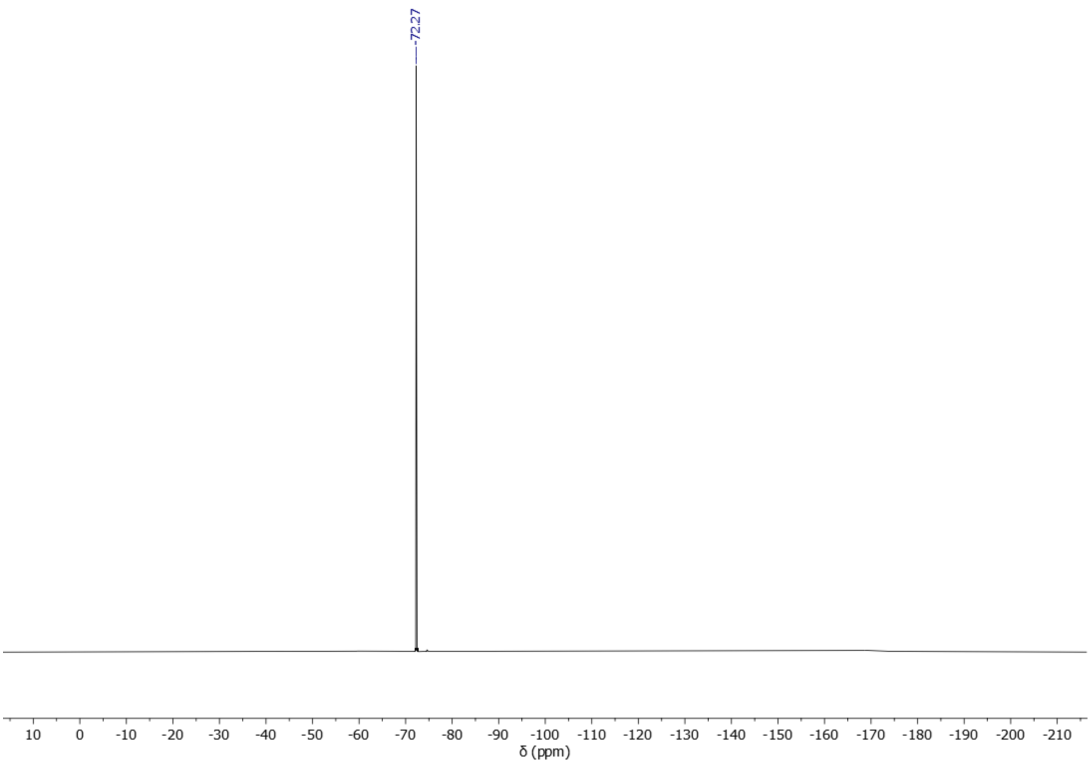


**Figure S27:** $^{19}$F NMR spectrum of [**3a**$^-$][Na$^+$] ($CD_3CN$, 25 °C, 564 MHz).

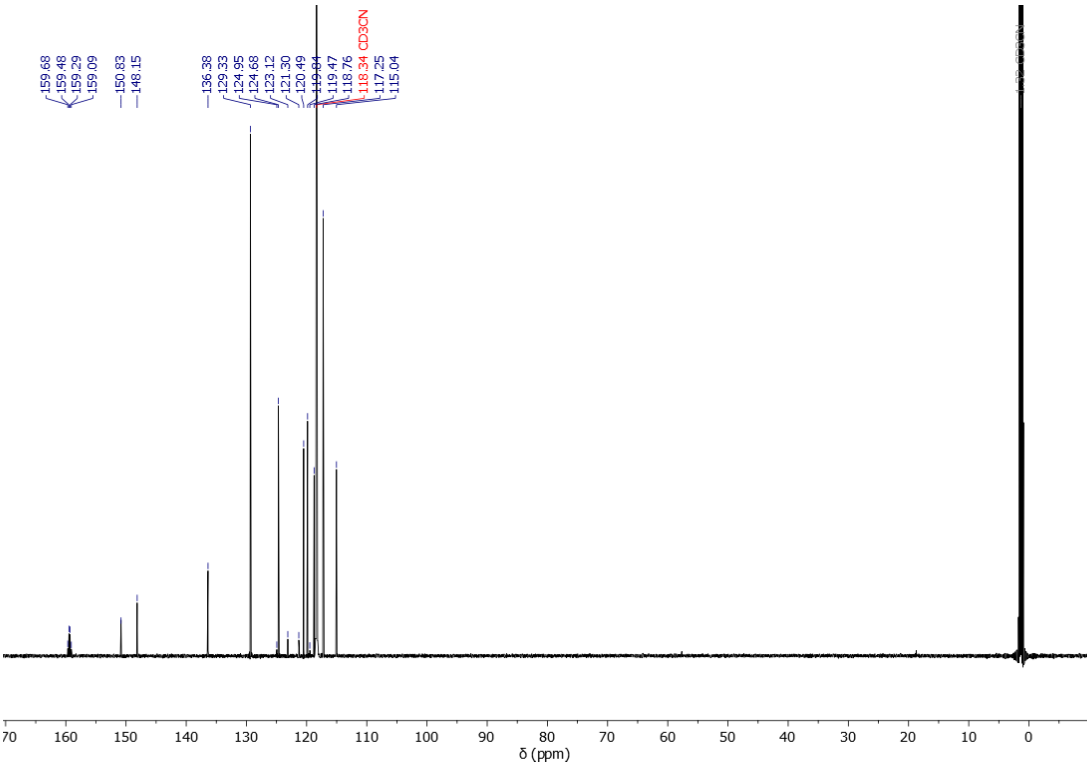


**Figure S28:** $^{13}$C NMR spectrum of [**3a**$^-$][Na$^+$] ($CD_3CN$, 25 °C, 151 MHz).

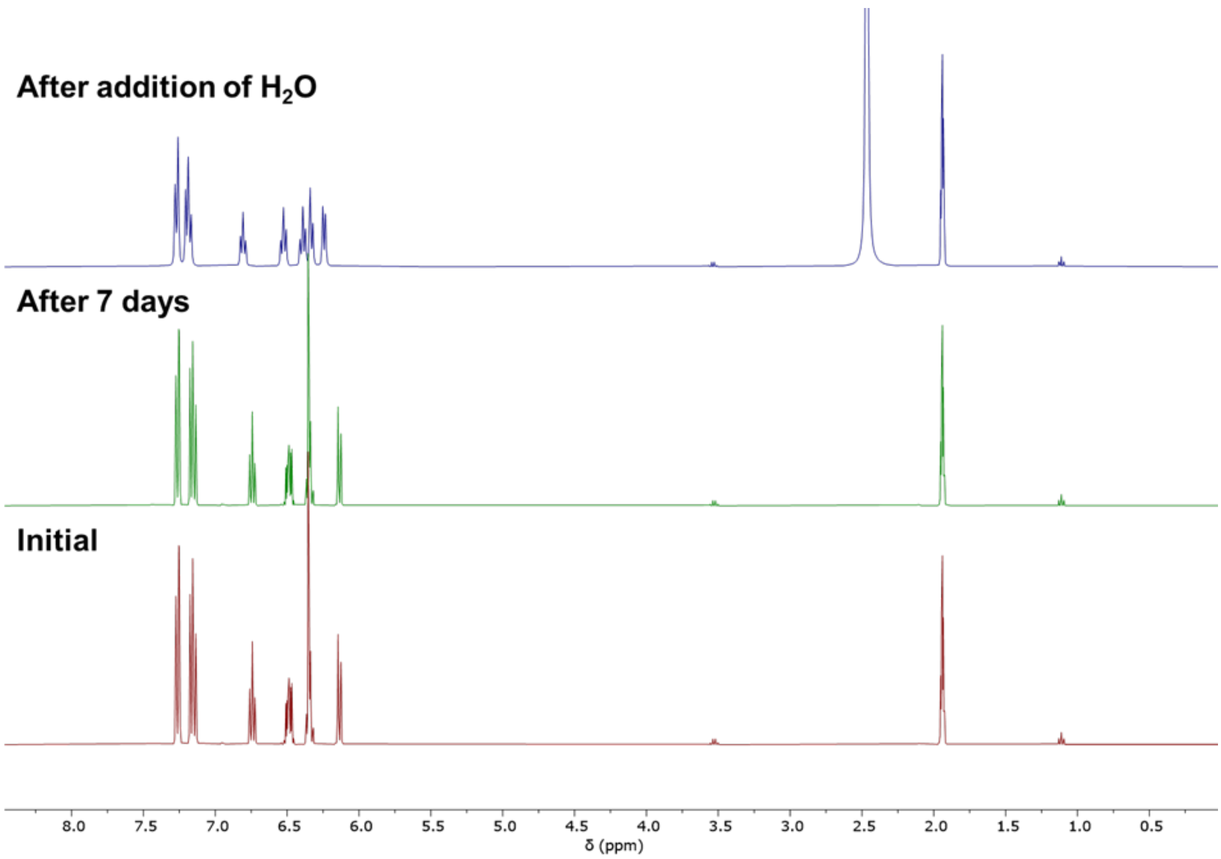


**Figure S29:** Comparison of $^{1}H$ NMR spectra of [**3a**$^{-}$][$Na^{+}$] showing no sign of decomposition after seven days in a Teflon-sealed NMR tube under $N_2$ atmosphere and after addition of an excess of $H_2O$ ($CD_3CN$, 25 °C, 400 MHz).

## UV-VIS Spectroscopy

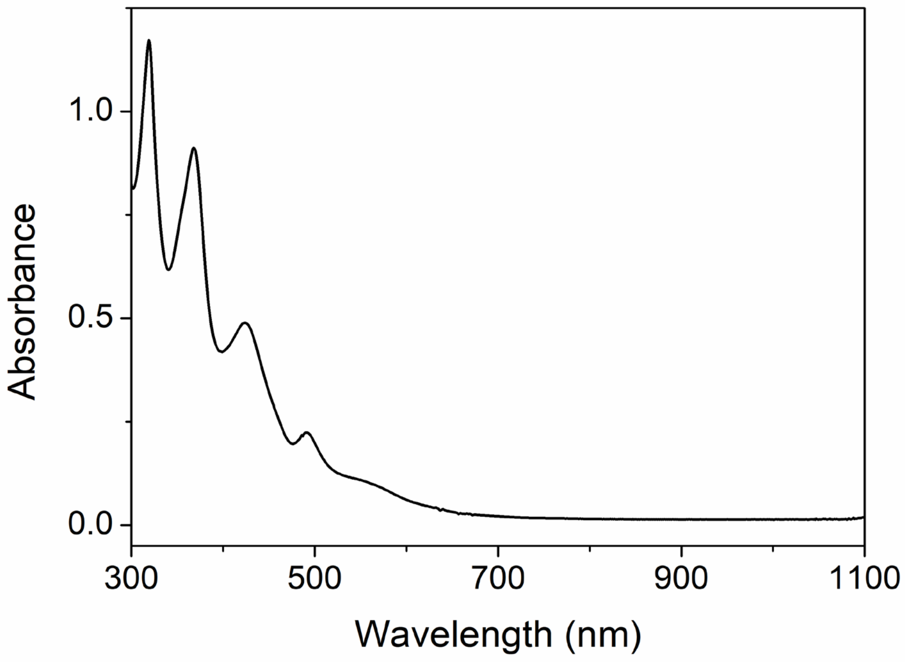


**Figure S30:** Absorption spectra for **1** (100 µM) in $CH_3CN$.

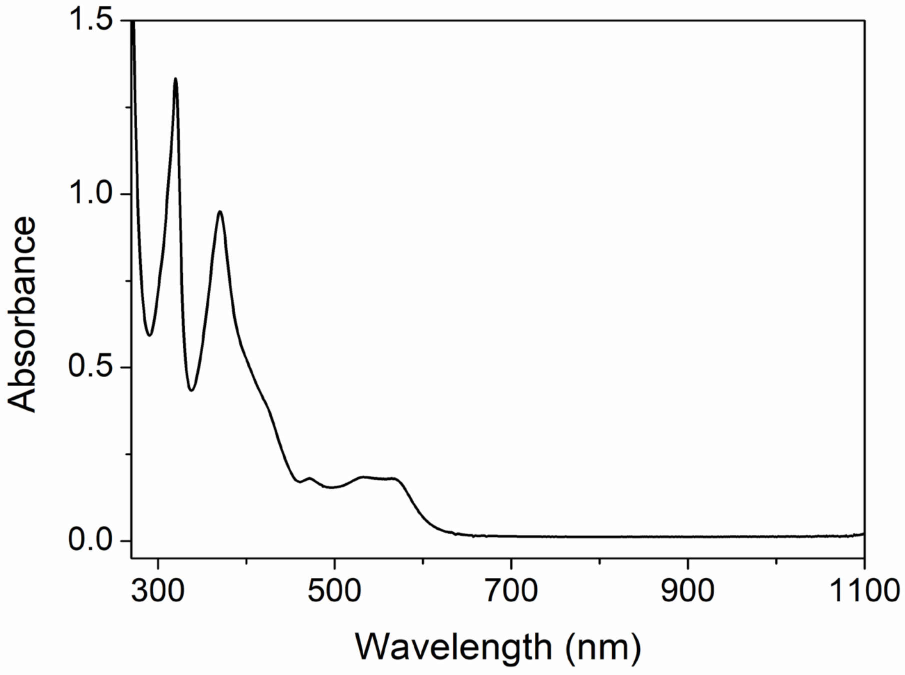


**Figure S31:** Absorption spectra for **2a** (100 µM) in $CH_3CN$.

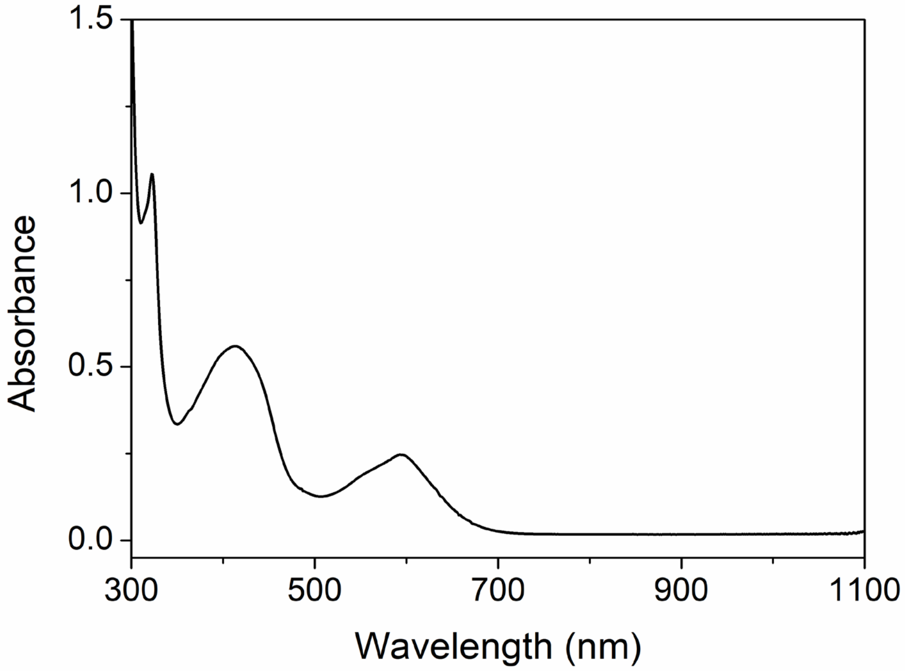


**Figure S32:** Absorption spectra for **2b** (100 μM) in $CH_3CN$.

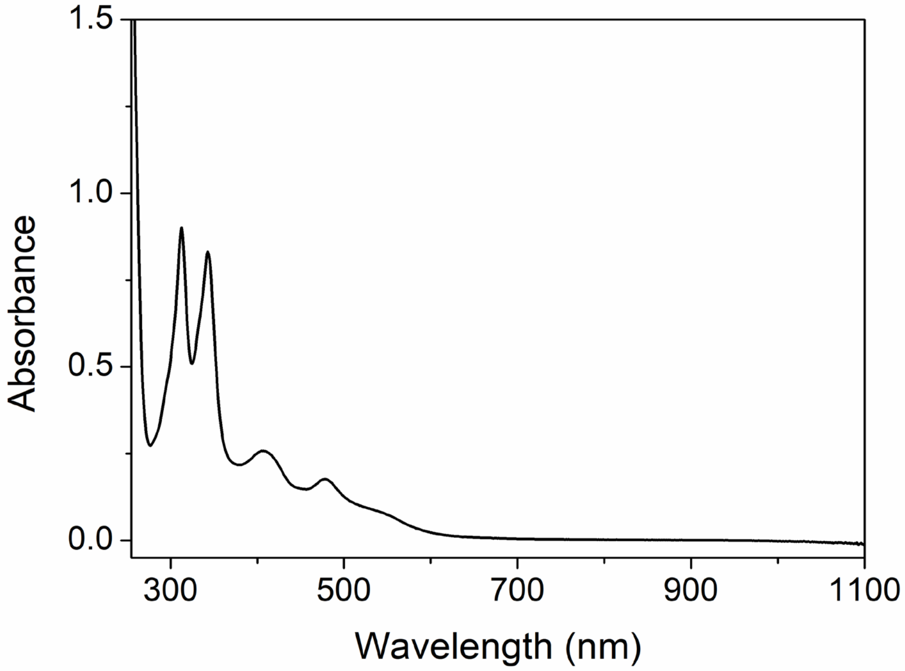


**Figure S33:** Absorption spectra for **3a** (100 μM) in $CH_3CN$.

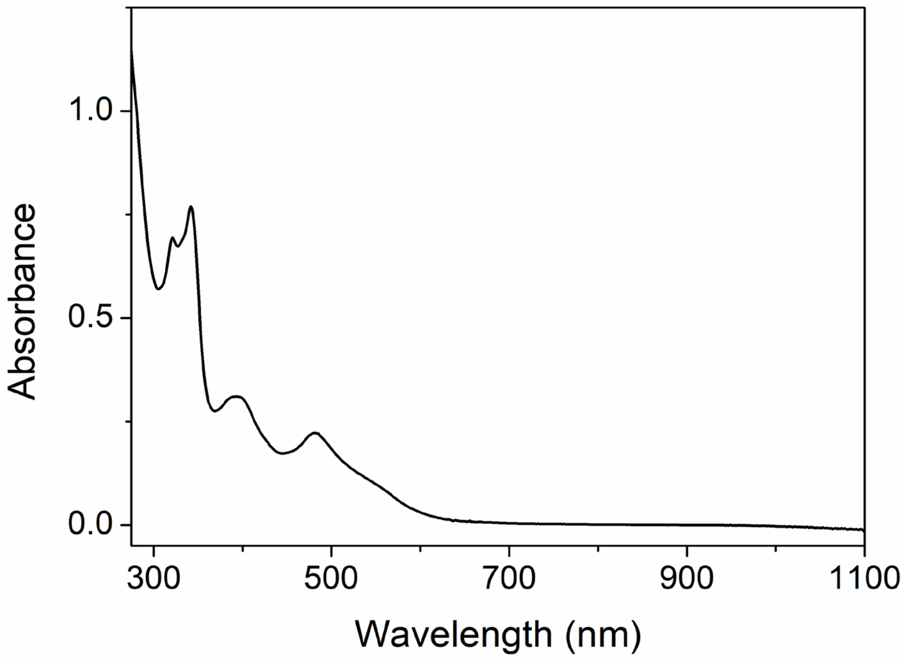


**Figure S34:** Absorption spectra for **3b** (100 μM) in $CH_3CN$.

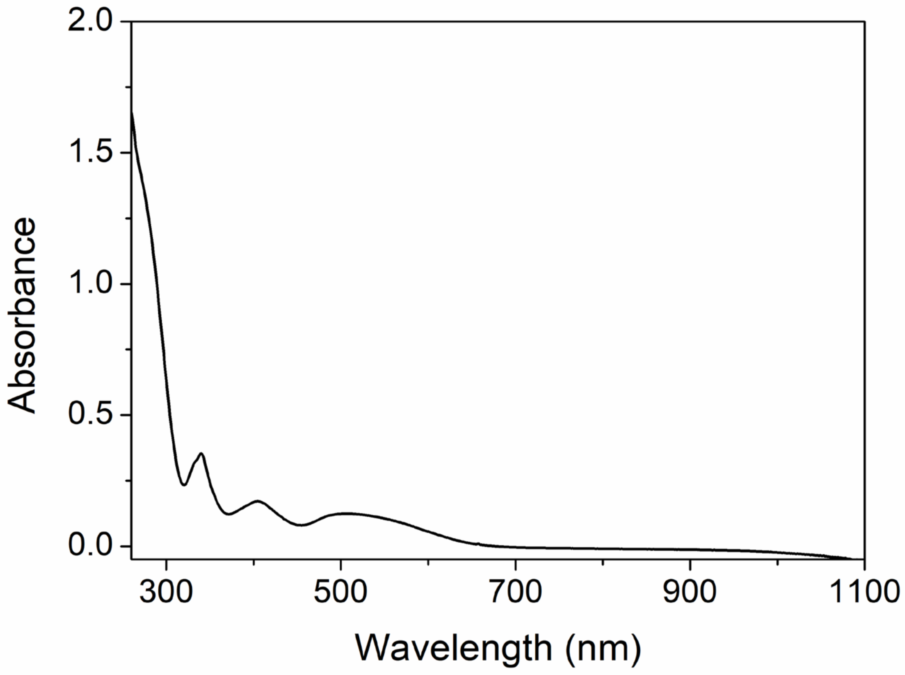


**Figure S35:** Absorption spectra for **3c** (100 μM) in $CH_3CN$.

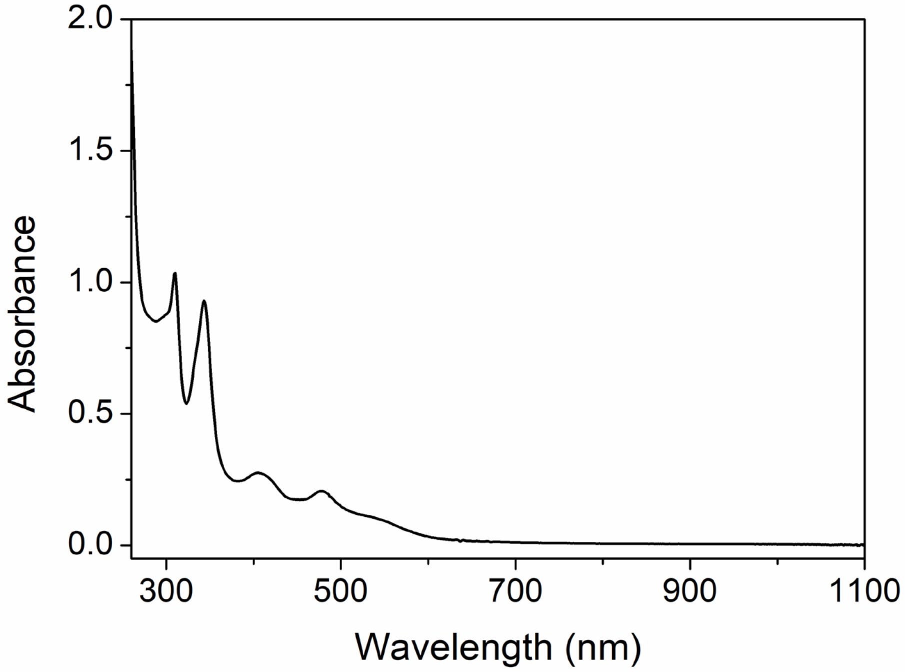


**Figure S36:** Absorption spectra for **3d** (100 µM) in $CH_3CN$.

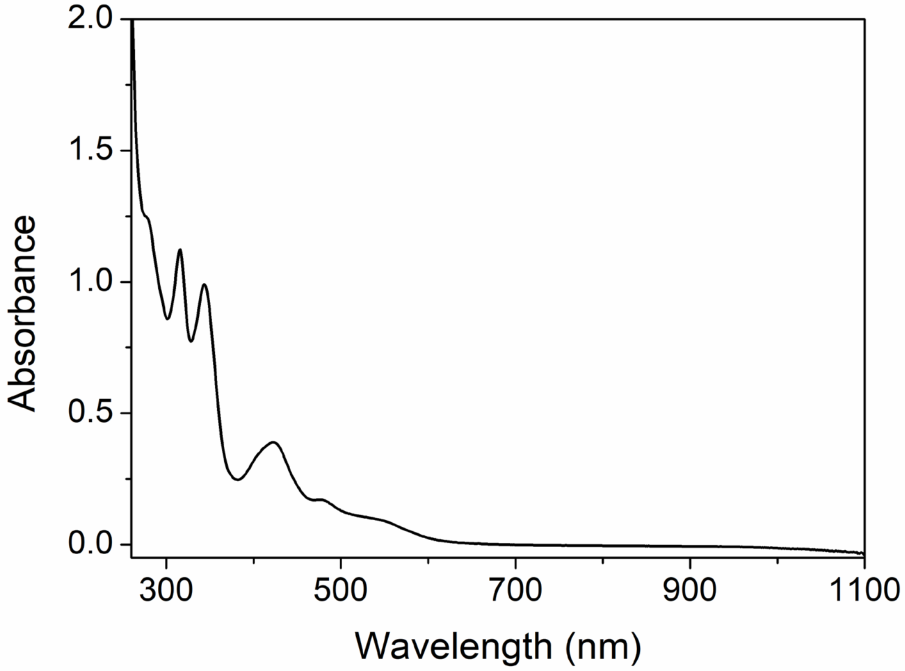


**Figure S37:** Absorption spectra for **3e** (100 µM) in $CH_3CN$.

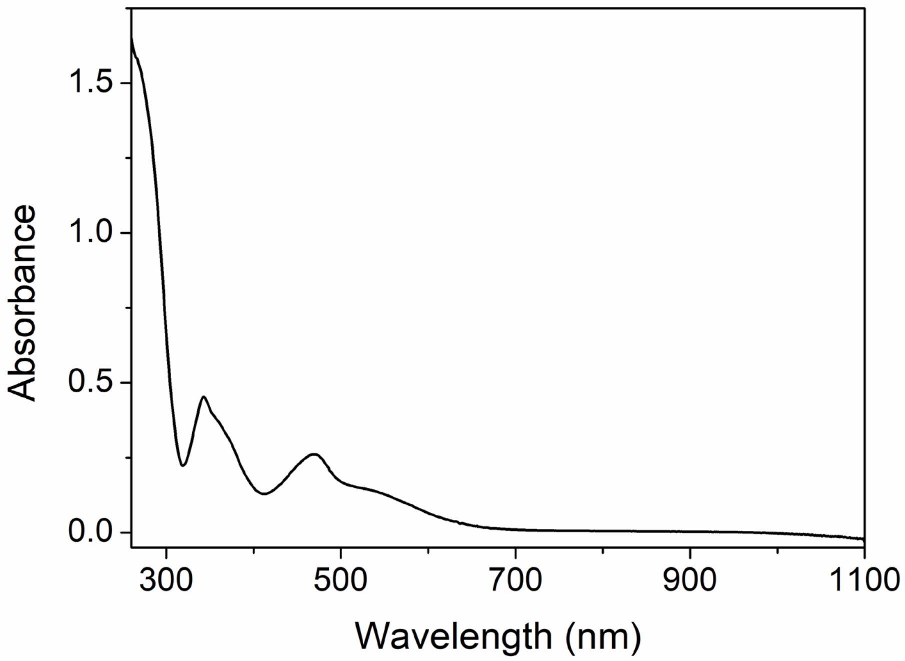


**Figure S38:** Absorption spectra for **3f** (100 μM) in $CH_3CN$.

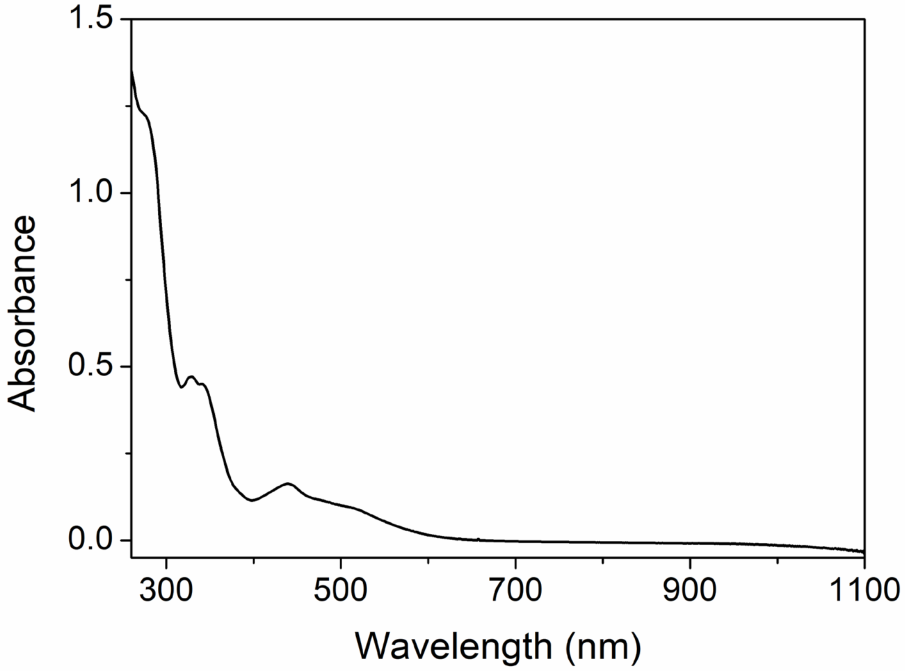


**Figure S39:** Absorption spectra for **3g** (100 μM) in $CH_3CN$.

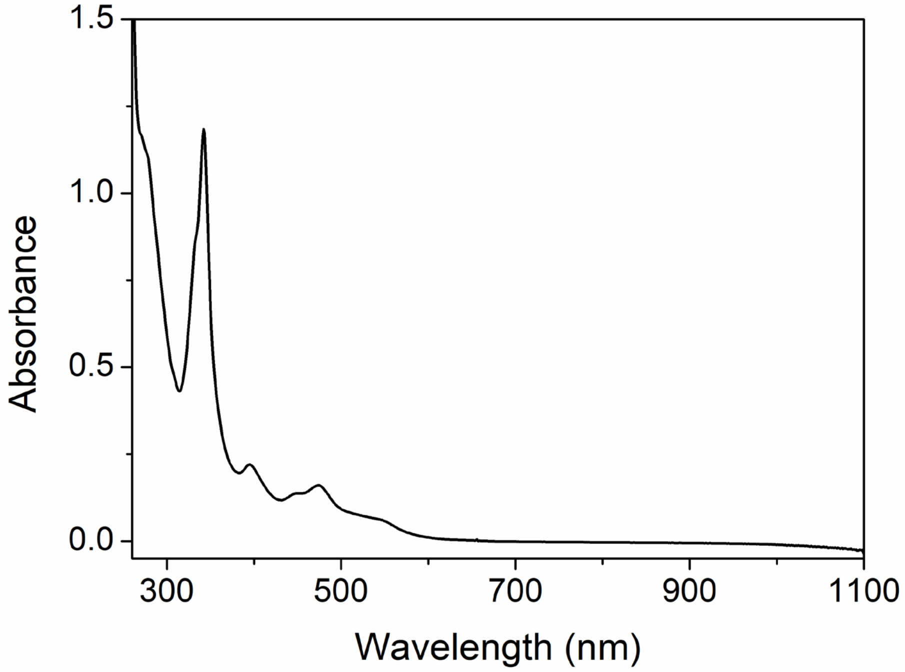


**Figure S40:** Absorption spectra for **3h** (100 µM) in $CH_3CN$.

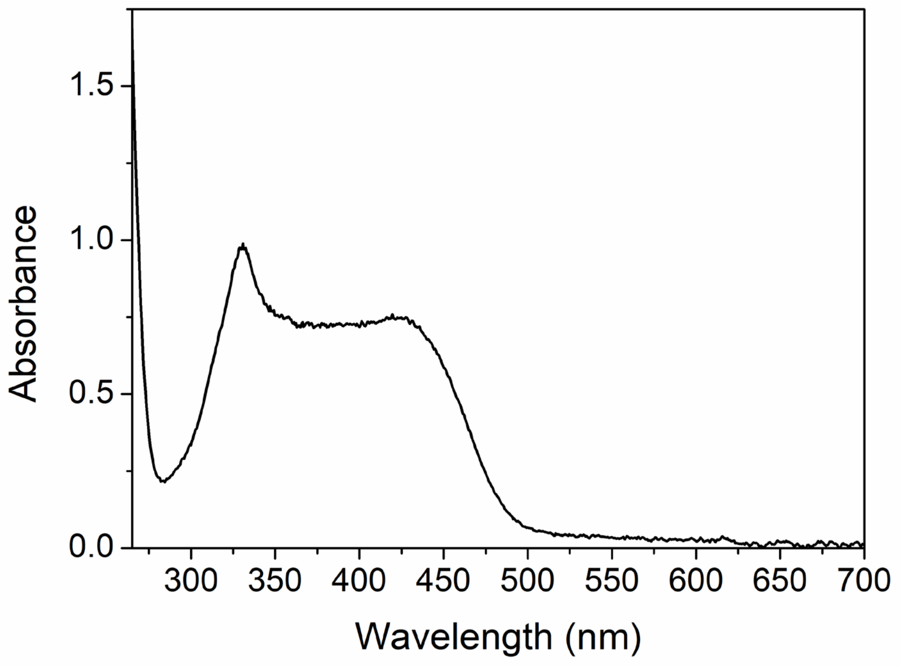


**Figure S41:** Absorption spectra for [**3a**$^+$][$BF_4$] in $CH_3CN$.

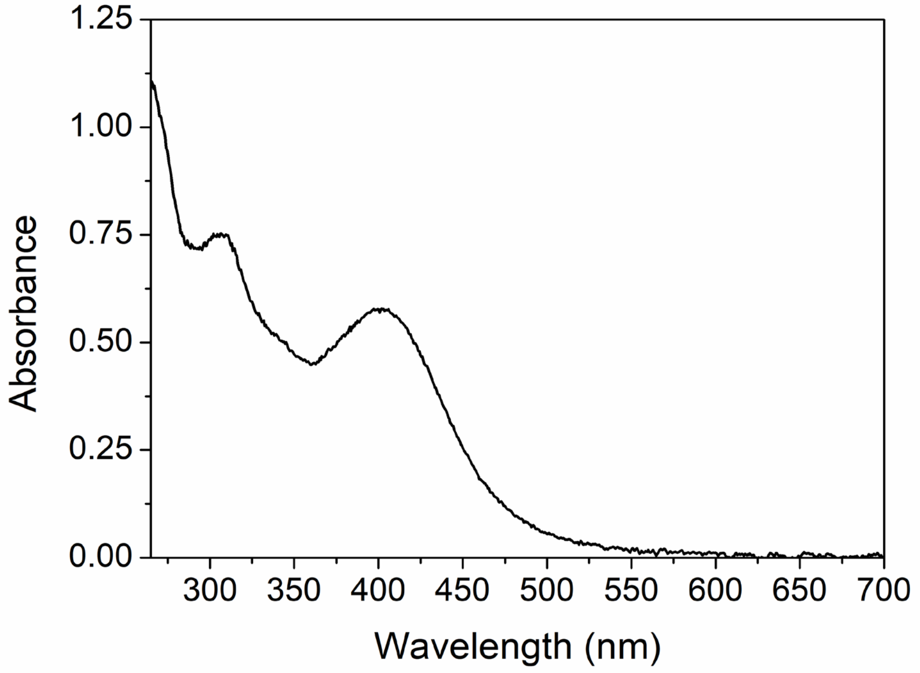


**Figure S42:** Absorption spectra for [**3a**$^-$][Na] in $CH_3CN$.

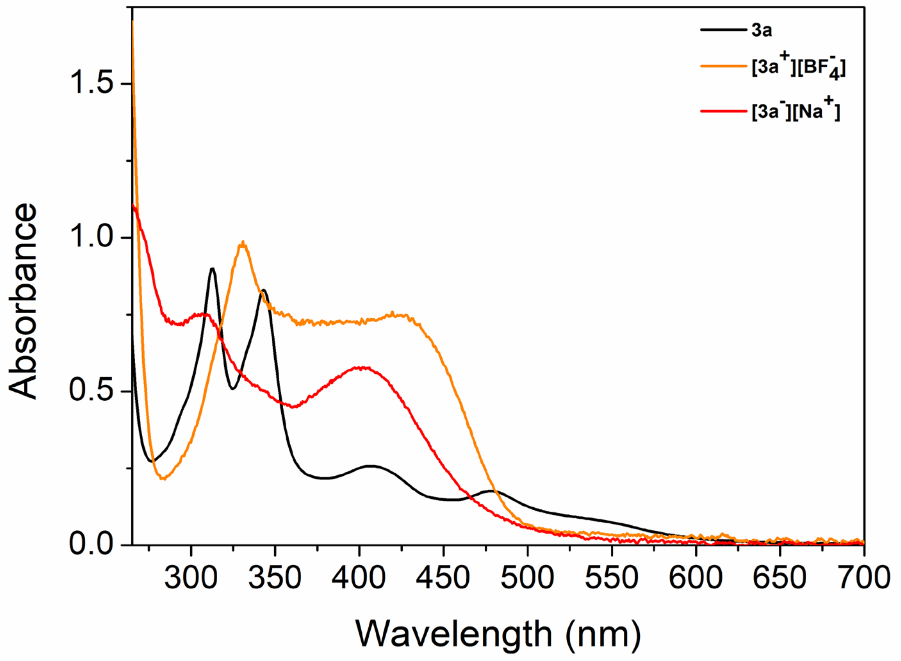


**Figure S43:** Absorption spectra for **3a**, [**3a**$^+$][$BF_4$] and [**3a**$^-$][Na] in $CH_3CN$.

## EPR Spectroscopy

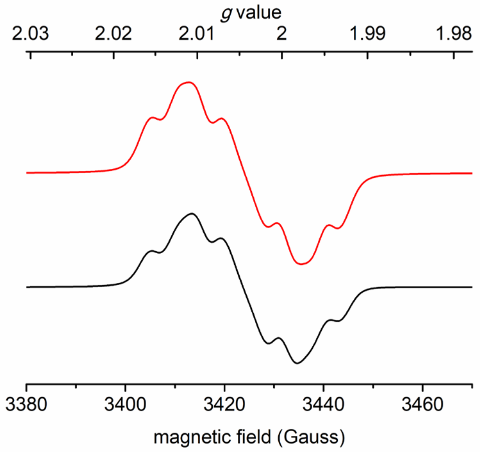


**EPR 3a** (1mM, 25 °C, DCM): $g_{soln}$ = 2.0043, $a_{N(1)}$ = 7.86, $a_{N(2)}$ = 4.19, $a_{N(4)}$ = 5.17, $a_F$ = 0.90 G and $\Delta H_{pp}$ = 2.80 G.

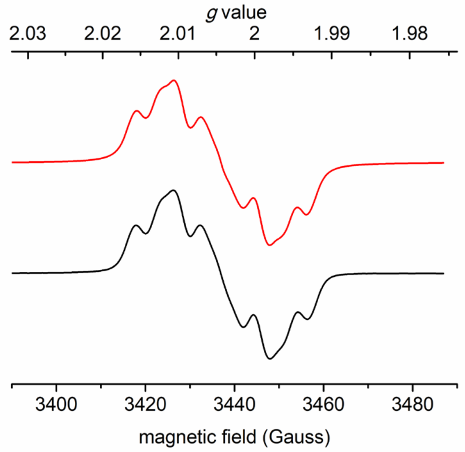


**EPR 3b** (1mM, 25 °C, DCM): $g_{soln}$ = 2.0043, $a_{N(1)}$ = 8.12, $a_{N(2)}$ = 4.13, $a_{N(4)}$ = 5.17, $a_F$ = 1.58 G and $\Delta H_{pp}$ = 1.41 G.

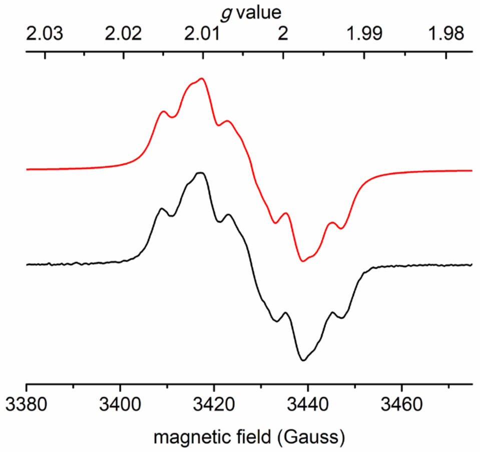


**EPR 3c** (1mM, 25 °C, DCM): $g_{soln}$ = 2.0038, $a_{N(1)}$ = 8.08, $a_{N(2)}$ = 4.06, $a_{N(4)}$ = 5.15, $a_F$ = 1.58 G and $\Delta H_{pp}$ = 1.34 G.

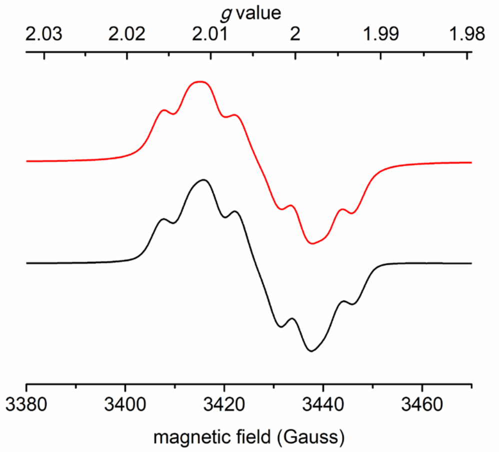


**EPR 3d** (1mM, 25 °C, DCM): $g_{soln}$ = 2.0044, $a_{N(1)}$ = 7.94, $a_{N(2)}$ = 5.21, $a_{N(4)}$ = 4.24, $a_F$ = 1.63 G and $\Delta H_{pp}$ = 1.28 G.

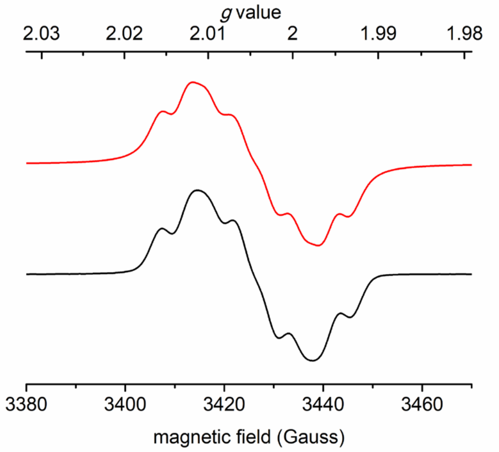


**EPR 3e** (1mM, 25 °C, DCM): $g_{soln}$ = 2.0044, $a_{N(1)}$ = 7.80, $a_{N(2)}$ = 5.33, $a_{N(4)}$ = 4.18, $a_F$ = 1.35 G and $\Delta H_{pp}$ = 1.27 G.

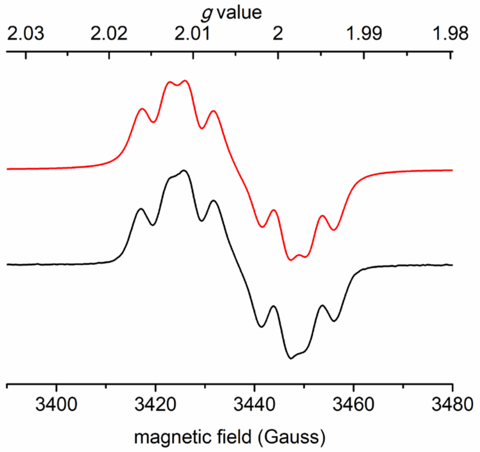


**EPR 3f** (1mM, 25 °C, DCM): $g_{soln}$ = 2.0047, $a_{N(1)}$ = 8.14, $a_{N(2)}$ = 4.21, $a_{N(4)}$ = 5.37, $a_F$ = 1.30 G and $\Delta H_{pp}$ = 1.74 G.

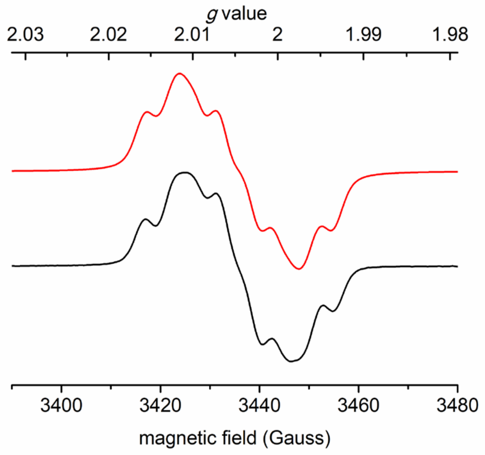


**EPR 3g** (1mM, 25 °C, DCM): $g_{soln}$ = 2.0045, $a_{N(1)}$ = 7.66, $a_{N(2)}$ = 4.00, $a_{N(4)}$ = 5.22, $a_F$ = 1.67 G and $\Delta H_{pp}$ = 1.23 G.

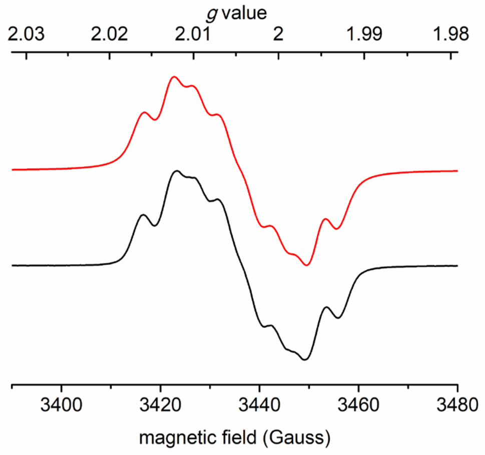


**EPR 3h** (1mM, 25 °C, DCM): $g_{soln}$ = 2.0044, $a_{N(1)}$ = 7.73, $a_{N(2)}$ = 4.47, $a_{N(4)}$ = 5.48, $a_F$ = 1.55 G and $\Delta H_{pp}$ = 1.56 G.

## X-ray Crystallography

Suitable crystals of **3c**, **3h** and [**3a**$^+$][$BF_4$] were mounted on a cryo-loop and transferred into the cold nitrogen stream of a Bruker D8 Venture diffractometer. Data collection and reduction was done using the Bruker software suite APEX3.[9] The final unit cell was obtained from the xyz centroids of 7575 (**3c**), 9886 (**3h**) and 9402 ([**3a**$^+$][$BF_4$]) reflections after integration. A multi-scan absorption correction was applied, based on the intensities of symmetry-related reflections measured at different angular settings (SADABS).[9] The structures were solved by dual space methods using the program SHELXT.[10] Structure refinement was performed with the program package SHELXL.[11] The hydrogen atoms were generated by geometrical considerations and constrained to idealized geometries and allowed to ride on their carrier atoms with an isotropic displacement parameter related to the equivalent displacement parameter of their carrier atoms. Crystal data and details on data collection and refinement are presented in Table S1.

**Table S1**: Crystallographic data for **3c**, **3h** and [**3a**$^+$][$BF_4$].

| | | | |
|---|---|---|---|
| chem formula | $C_{14}H_{10}F_3N_4$ | $C_{14}H_7N_3F_3Cl_2$ | $C_{14}H_9BF_7N_3$ |
| $M_r$ | 291.26 | 345.13 | 363.05 |
| cryst syst | monoclinic | triclinic | triclinic |
| color, habit | dark red, rhombic plate | red, block | Yellow/orange, plate |
| size (mm) | 0.099 x 0.128 x 0.199 | 0.213 x 0.131 x 0.096 | 0.343 x 0.296 x 0.032 |
| space group | P21/n | P-1 | P-1 |
| a (Å) | 8.3599(2) | 8.4065(3) | 7.4258(3) |
| b (Å) | 10.2236(3) | 9.3291(3) | 7.7103(3) |
| c (Å) | 15.4801(4) | 10.5635(3) | 14.1805(6) |
| α (°) | 90 | 65.794(1) | 93.610(2) |
| β (°) | 97.8030(10) | 72.794(1) | 98.635(2) |
| γ (°) | 90 | 66.953(1) | 112.966(2) |
| V (Å$^3$) | 1310.81(6) | 686.20(4) | 2406.36(9) |
| Z | 4 | 2 | 2 |
| $\rho_{calc}$, g.cm$^{-3}$ | 1.76 | 1.670 | 1.646 |
| Radiation [Å] | 1.54178 | Cu Kα 1.54178 | Cu $K_\alpha$ 1.54178 |
| $\mu$(Cu $K_\alpha$), mm$^{-1}$ | 1.053 | 4.592 | 1.450 |
| F(000) | 596 | 346 | 364 |
| temp (K) | 100(2) | 100(2) | 100(2) |
| θ range (°) | 5.199 – 72.364 | 4.650 – 72.143 | 3.181 – 70.218 |
| data collected (h,k,l) | -10:10; -12:12; -19:18 | -10:10; -11:11; -13:13 | -9:9; -9:9; -17:17 |
| no. of rflns collected | 23470 | 17177 | 15852 |
| no. of indpndt reflns | 2569 | 2639 | 2767 |
| observed reflns $F_o \geq 2.0\ \sigma(F_o)$ | 2317 | 2592 | 2520 |
| R(F) (%) | 3.53 | 3.07 | 3.28 |
| wR(F$^2$) (%) | 9.11 | 7.80 | 8.76 |
| GooF | 1.048 | 1.028 | 1.028 |
| weighting a,b | 0.0419, 0.3796 | 0.0303, 0.5904 | 0.0410, 0.2717 |
| params refined | 256 | 199 | 226 |
| min, max resid dens | -0.176, 0.209 | -0.321, 0397 | -0.274, 0.248 |

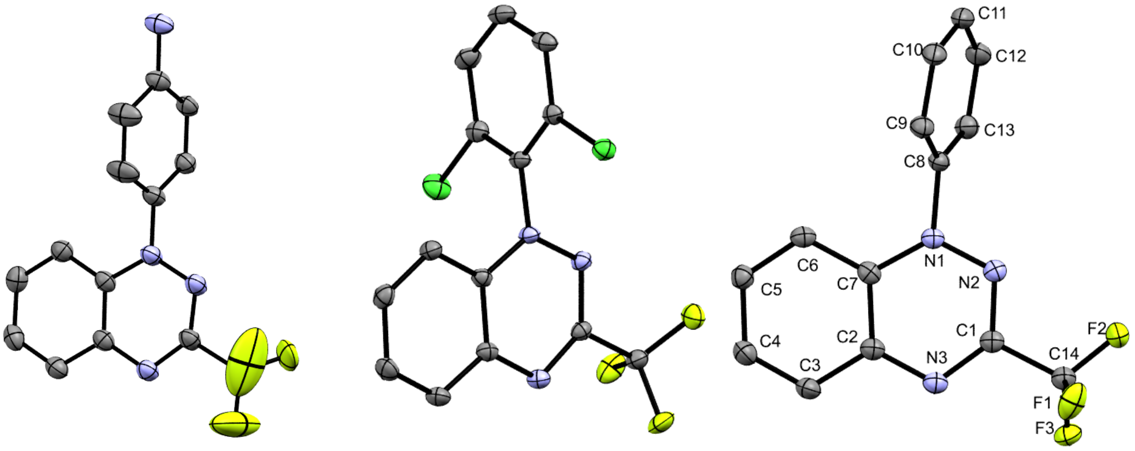


**Figure S44:** Molecular structures of compounds **3c** (left), **3h** (middle) and [**3a**$^{+}$][$BF_4$]. Showing 50% probability ellipsoids; hydrogen atoms and $BF_4^-$ anion (for [**3a**$^{+}$][$BF_4$]) are omitted for clarity.

**Table S2:** Selected bond lengths (Å) in compounds **3a**[7] (at 75 K) and [**3a**$^{+}$][$BF_4$] (at 100 K).

| Bond | 3a | [3a$^{+}$][$BF_4$] |
|---|---|---|
| **C14-F1** | 1.343(2) | 1.335(2) |
| **C14-F2** | 1.334(3) | 1.329(2) |
| **C14-F3** | 1.328(2) | 1.326(1) |
| **C14-C1** | 1.523(3) | 1.516(2) |
| **C1-N3** | 1.318(2) | 1.323(2) |
| **C1-N2** | 1.327(2) | 1.330(2) |
| **C2-N3** | 1.387(3) | 1.341(2) |
| **C2-C3** | 1.389(4) | 1.417(2) |
| **C2-C7** | 1.419(2) | 1.437(2) |
| **C3-C4** | 1.381(3) | 1.362(2) |
| **C4-C5** | 1.397(2) | 1.428(2) |
| **C5-C6** | 1.376(4) | 1.368(2) |
| **C6-C7** | 1.401(3) | 1.413(2) |
| **C7-N1** | 1.390(2) | 1.359(2) |
| **N1-N2** | 1.370(2) | 1.318(1) |
| **N1-C8** | 1.440(2) | 1.459(1) |
| **C8-C9** | 1.390(2) | 1.383(2) |
| **C8-C13** | 1.385(2) | 1.383(2) |
| **C9-C10** | 1.388(2) | 1.390(2) |
| **C10-C11** | 1.385(3) | 1.383(2) |
| **C11-C12** | 1.386(2) | 1.392(2) |
| **C12-C13** | 1.389(2) | 1.387(2) |

# Cyclic Voltammetry

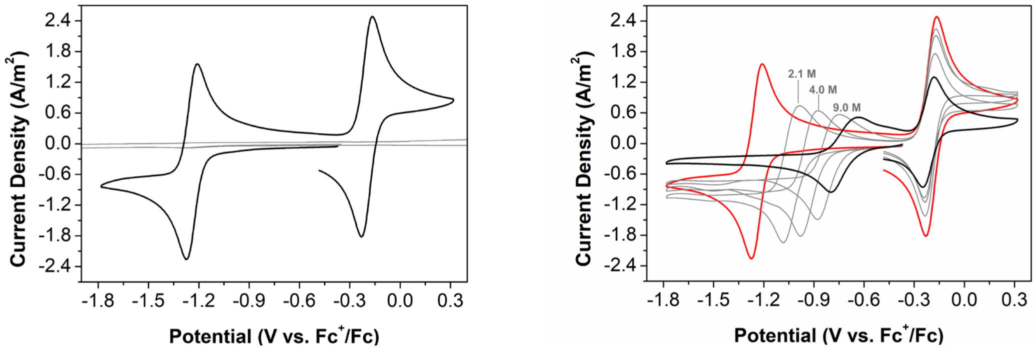


**Figure S45:** (left) Cyclic voltammogram of **1** (1 mM, $CH_3CN$, 0.1 M $[Bu_4N][PF_6]$ electrolyte solution). (right) Cyclic voltammograms of 1 mM **1** in pure acetonitrile (red trace) and with water added up to 18.5M (black trace), measured at a GC working electrode with 0.1 M $[Bu_4N][PF_6]$ as supporting electrolyte with a scan rate of 50 mV/s.

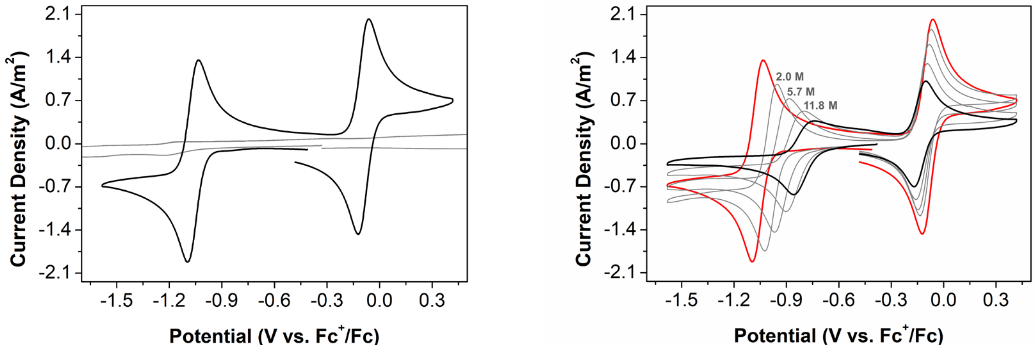


**Figure S46:** (left) Cyclic voltammogram of **2a** (1 mM, $CH_3CN$, 0.1 M $[Bu_4N][PF_6]$ electrolyte solution). (right) Cyclic voltammograms of 1 mM **2a** in pure acetonitrile (red trace) and with water added up to 18.5M (black trace), measured at a GC working electrode with 0.1 M $[Bu_4N][PF_6]$ as supporting electrolyte with a scan rate of 50 mV/s.

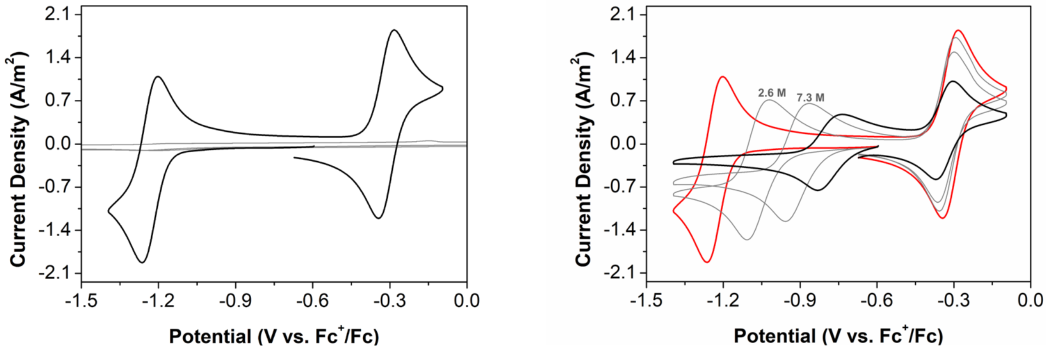


**Figure S47:** (left) Cyclic voltammogram of **2b** (1 mM, $CH_3CN$, 0.1 M $[Bu_4N][PF_6]$ electrolyte solution). (right) Cyclic voltammograms of 1 mM **2b** in pure acetonitrile (red trace) and with water added up to 18.5M (black trace), measured at a GC working electrode with 0.1 M $[Bu_4N][PF_6]$ as supporting electrolyte with a scan rate of 50 mV/s.

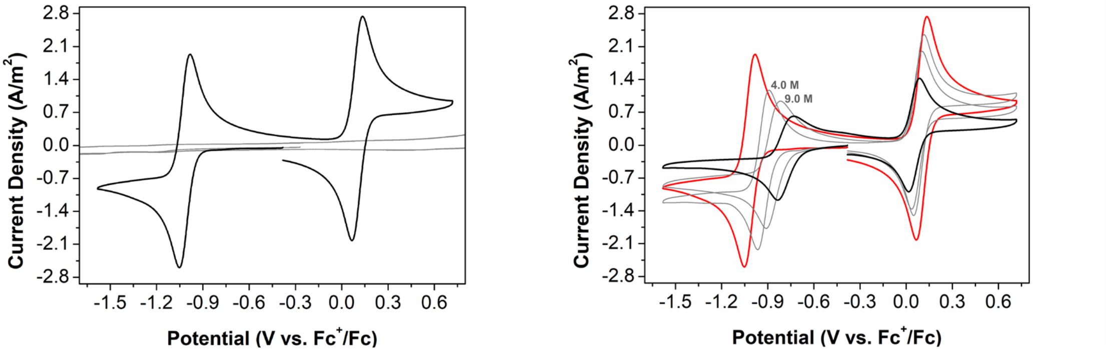


**Figure S48:** (left) Cyclic voltammogram of **3a** (1 mM, $CH_3CN$, 0.1 M $[Bu_4N][PF_6]$ electrolyte solution). (right) Cyclic voltammograms of 1 mM **3a** in pure acetonitrile (red trace) and with water added up to 18.5M (black trace), measured at a GC working electrode with 0.1 M $[Bu_4N][PF_6]$ as supporting electrolyte with a scan rate of 50 mV/s.

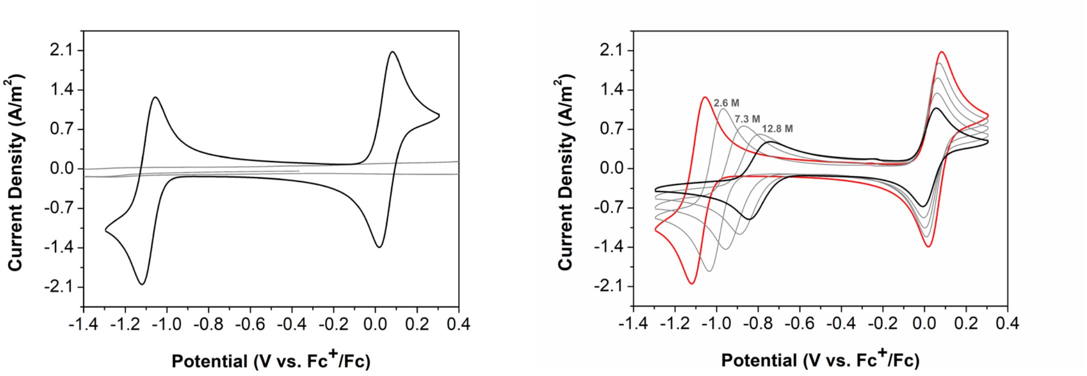


**Figure S49:** (left) Cyclic voltammogram of **3b** (1 mM, $CH_3CN$, 0.1 M $[Bu_4N][PF_6]$ electrolyte solution). (right) Cyclic voltammograms of 1 mM **3b** in pure acetonitrile (red trace) and with water added up to 18.5M (black trace), measured at a GC working electrode with 0.1 M $[Bu_4N][PF_6]$ as supporting electrolyte with a scan rate of 50 mV/s.

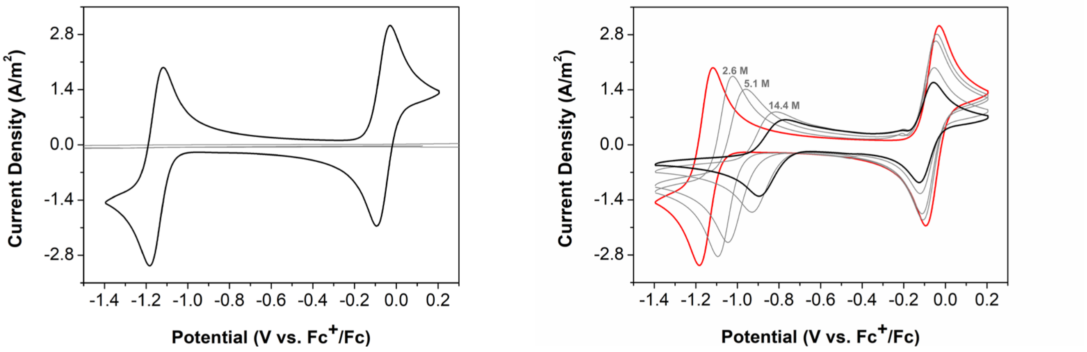


**Figure S50:** (left) Cyclic voltammogram of **3c** (1 mM, $CH_3CN$, 0.1 M $[Bu_4N][PF_6]$ electrolyte solution). (right) Cyclic voltammograms of 1 mM **3c** in pure acetonitrile (red trace) and with water added up to 18.5M (black trace), measured at a GC working electrode with 0.1 M $[Bu_4N][PF_6]$ as supporting electrolyte with a scan rate of 50 mV/s.

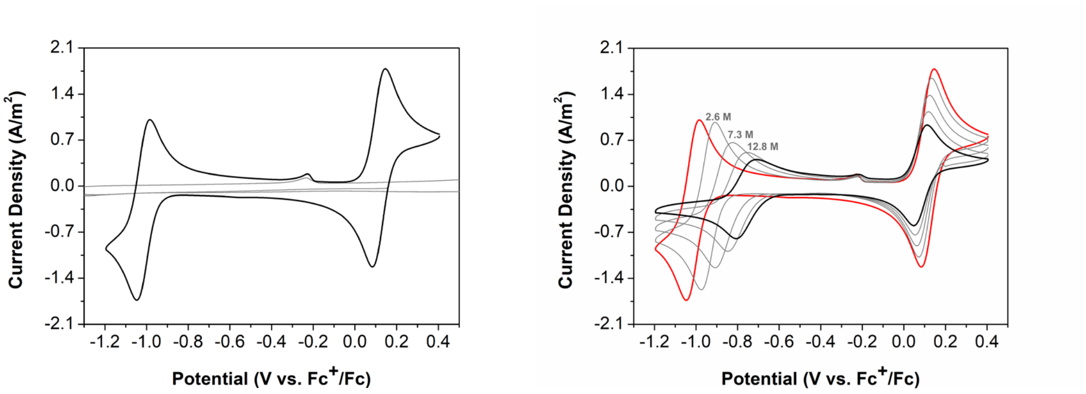


**Figure S51:** (left) Cyclic voltammogram of **3d** (1 mM, $CH_3CN$, 0.1 M $[Bu_4N][PF_6]$ electrolyte solution). (right) Cyclic voltammograms of 1 mM **3d** in pure acetonitrile (red trace) and with water added up to 18.5M (black trace), measured at a GC working electrode with 0.1 M $[Bu_4N][PF_6]$ as supporting electrolyte with a scan rate of 50 mV/s.

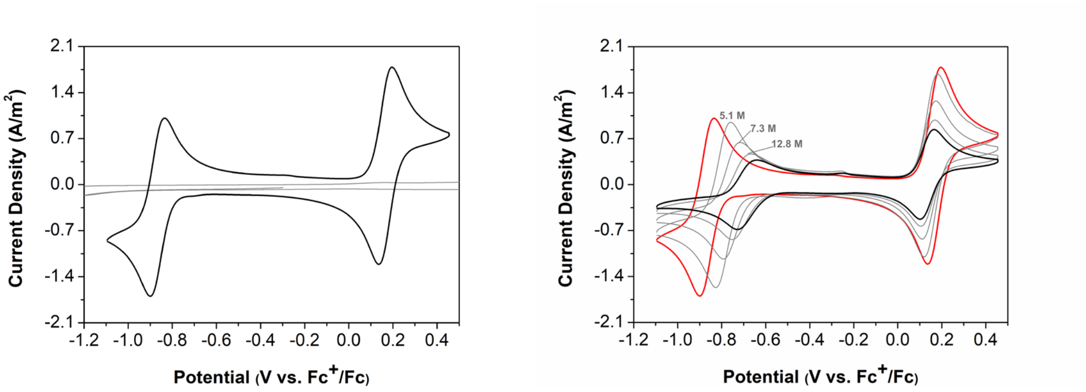


**Figure S52:** (left) Cyclic voltammogram of **3e** (1 mM, $CH_3CN$, 0.1 M $[Bu_4N][PF_6]$ electrolyte solution). (right) Cyclic voltammograms of 1 mM **3e** in pure acetonitrile (red trace) and with water added up to 18.5M (black trace), measured at a GC working electrode with 0.1 M $[Bu_4N][PF_6]$ as supporting electrolyte with a scan rate of 50 mV/s.

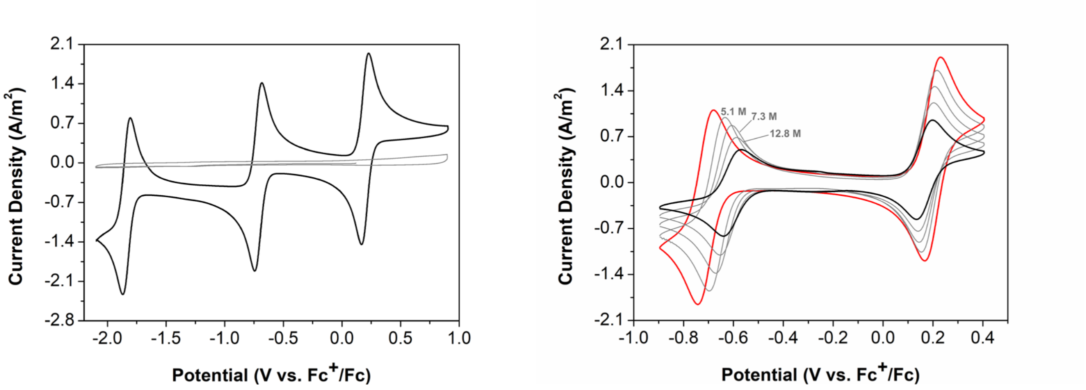


**Figure S53:** (left) Cyclic voltammogram of **3f** (1 mM, $CH_3CN$, 0.1 M $[Bu_4N][PF_6]$ electrolyte solution). (right) Cyclic voltammograms of 1 mM **3f** in pure acetonitrile (red trace) and with water added up to 18.5M (black trace), measured at a GC working electrode with 0.1 M $[Bu_4N][PF_6]$ as supporting electrolyte with a scan rate of 50 mV/s.

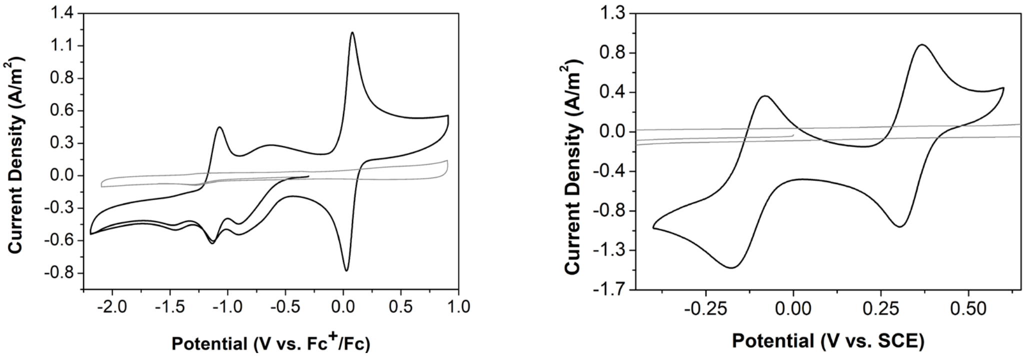


**Figure S54:** (left) Cyclic voltammogram of **3g** (1 mM, $CH_3CN$, 0.1 M $[Bu_4N][PF_6]$ electrolyte solution). (right) Cyclic voltammogram of **3g** (1 mM, $H_2O$, 0.1 M phosphate buffer at pH 7).

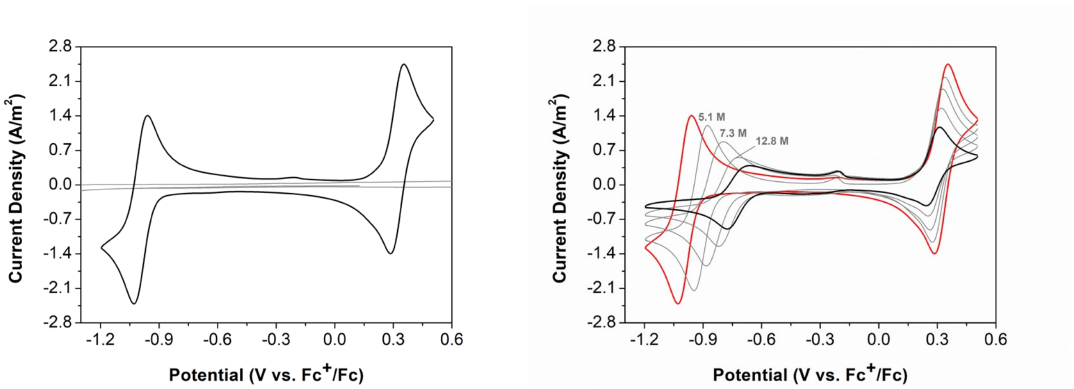


**Figure S55:** (left) Cyclic voltammogram of **3h** (1 mM, $CH_3CN$, 0.1 M $[Bu_4N][PF_6]$ electrolyte solution). (right) Cyclic voltammograms of 1 mM **3h** in pure acetonitrile (red trace) and with water added up to 18.5M (black trace), measured at a GC working electrode with 0.1 M $[Bu_4N][PF_6]$ as supporting electrolyte with a scan rate of 50 mV/s.

**Table S3. Cyclic voltammetric data for 1 mM Blatter radicals in MeCN and MeCN/$H_2O$ mixture (2:1 v/v).**

| Compound | $E_{1/2}(x^{0/-})$ (MeCN) | $E_{1/2}(x^{0/+})$ (MeCN) | *calc.* $E_{cell}$ (MeCN) | $E_{1/2}(x^{0/-})$ (MeCN/$H_2O$) | $E_{1/2}(x^{0/+})$ (MeCN/$H_2O$) | *calc.* $E_{cell}$ (MeCN/$H_2O$) |
|---|---|---|---|---|---|---|
| **1** | -1.24 | -0.20 | 1.04 | -0.71 | -0.21 | 0.50 |
| **2a** | -1.07 | -0.09 | 0.98 | -0.80 | -0.14 | 0.66 |
| **2b** | -1.24 | -0.31 | 0.93 | -0.78 | -0.34 | 0.44 |
| **3a** | -1.03 | 0.09 | 1.12 | -0.75 | 0.06 | 0.81 |
| **3b** | -1.09 | 0.05 | 1.14 | -0.79 | 0.02 | 0.82 |
| **3c** | -1.15 | -0.06 | 1.09 | -0.83 | -0.09 | 0.74 |
| **3d** | -1.02 | 0.12 | 1.13 | -0.76 | 0.08 | 0.84 |
| **3e** | -0.87 | 0.17 | 1.03 | -0.69 | 0.13 | 0.82 |
| **3f** | -0.71 | 0.20 | 0.91 | -0.60 | 0.17 | 0.77 |
| **3g** | -1.04[a] | 0.24 | - | - | - | - |
| **3h** | -0.99 | 0.32 | 1.32 | -0.72 | 0.28 | 1.0 |

[a] The reduction of **3g** is irreversible, and the peak potential is given.

## H-Cell Battery Tests

Charge-discharge tests of the Blatter radicals were performed in a custom H-cell with high surface area-to-volume ratio. The cell consisted of two electrolyte chambers separated by a glass frit (porosity 5, ~1.6 cm$^2$) to minimize crossover. Reticulated vitreous carbon (Duocel®, 45 ppi) were used as electrodes with an inter electrode distance of about 20 mm. For the battery tests, the electrolyte chambers were loaded with 5 mL active species in 0.3 M [$Bu_4N$][$PF_6$]/$CH_3CN$ and stirred continuously at 1400 rpm. The current was set such that theoretical charging and discharging times were 1 h each (1C-rate). Current densities were estimated using the membrane area size. Ohmic resistance of about 250 ohm was measured for all tests.

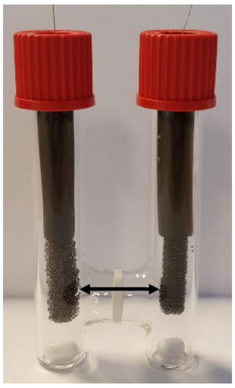

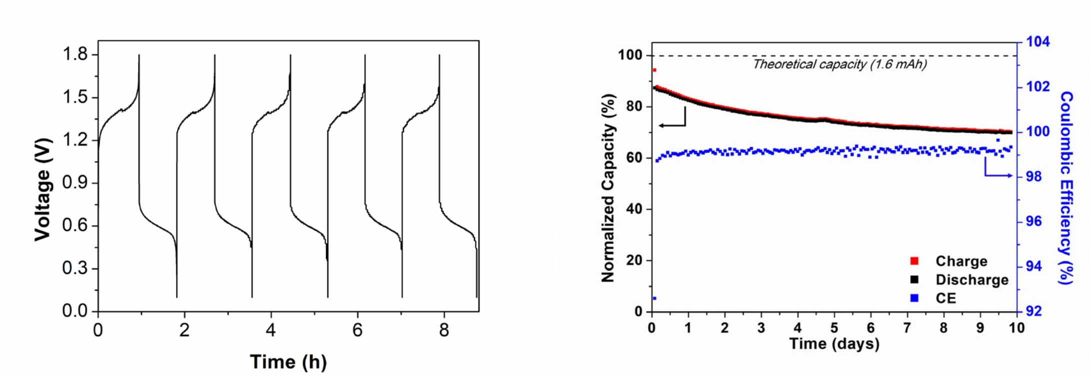


**Figure S56:** (left) Charge and discharge voltage curves for the first 5 cycles of a symmetrical non-flowing battery with 12 mM **1** in 0.3 M [$Bu_4N$][$PF_6$]/$CH_3CN$ as both negolyte and posolyte (cutoff voltages of 1.8 V and 0.1 V for charging and discharging, respectively, current ±1.6 mA, current density 1.0 mA/cm$^2$). (right) Normalized charging and discharging capacities (theoretical capacity of 1.6 mAh, 94% capacity reached in first charge) and coulombic efficiency for each cycle (157 cycles).

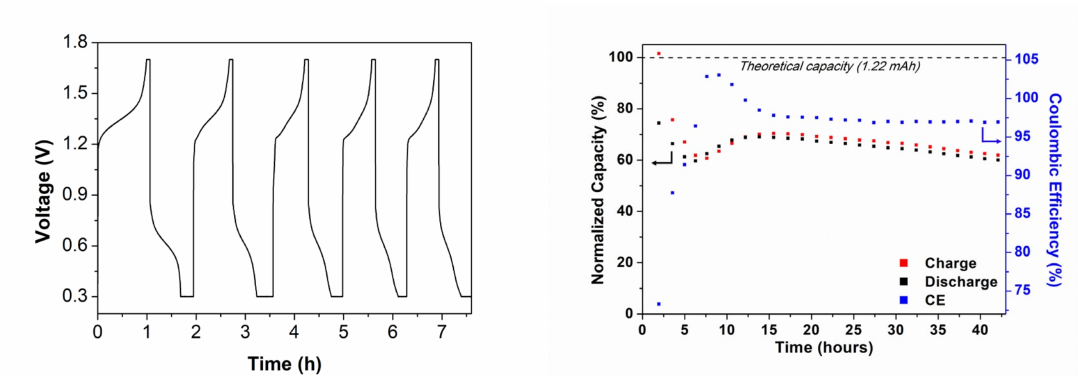


**Figure S57:** (left) Charge and discharge voltage curves for the first 5 cycles of a symmetrical non-flowing battery with 9 mM **2a** in 0.3 M [$Bu_4N$][$PF_6$]/$CH_3CN$ as both negolyte and posolyte (cutoff voltages of 1.7 V and 0.3 V for charging and discharging, respectively, current ±1.22 mA, current density 0.8 mA/cm$^2$). (right) Normalized charging and discharging capacities (theoretical capacity of 1.22 mAh, 101% capacity reached in first charge) and coulombic efficiency for each cycle (27 cycles).

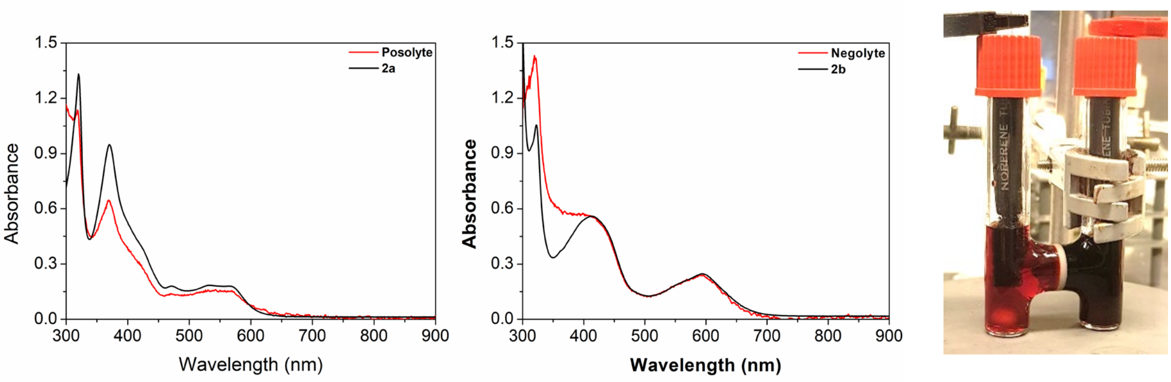


**Figure S58:** (left) Post-cycling absorption spectra of the posolyte solution after 27 cycles in $CH_3CN$ compared to the absorption spectrum of a fresh solution of **2a**. (middle) Post-cycling absorption spectra of the negolyte solution after 27 cycles in $CH_3CN$ compared to the absorption spectrum of a fresh solution of **2b**. (right) Post-cycling picture of the H-cell in discharged state.

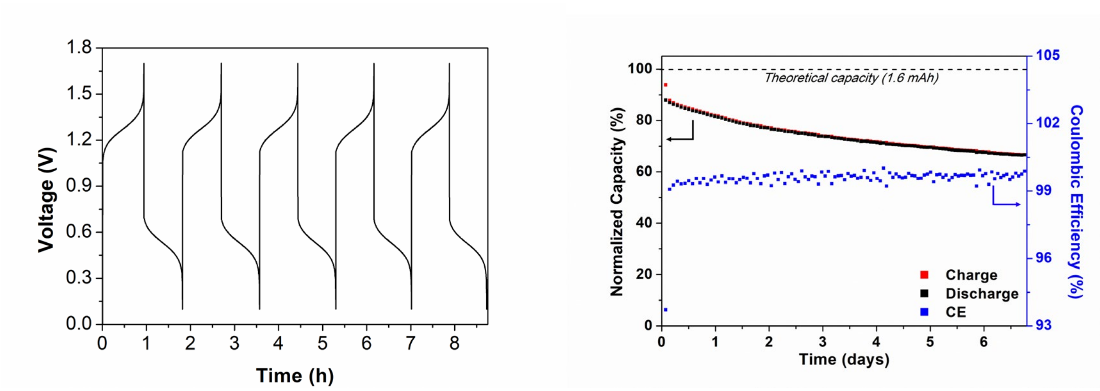


**Figure S59:** (left) Charge and discharge voltage curves for the first 5 cycles of a symmetrical non-flowing battery with 12 mM **2b** in 0.3 M $[Bu_4N][PF_6]/CH_3CN$ as both negolyte and posolyte (cutoff voltages of 1.7 V and 0.1 V for charging and discharging, respectively, current ±1.6 mA, current density 1.0 mA/cm$^2$). (right) Normalized charging and discharging capacities (theoretical capacity of 1.6 mAh, 94% capacity reached in first charge) and coulombic efficiency for each cycle (112 cycles).

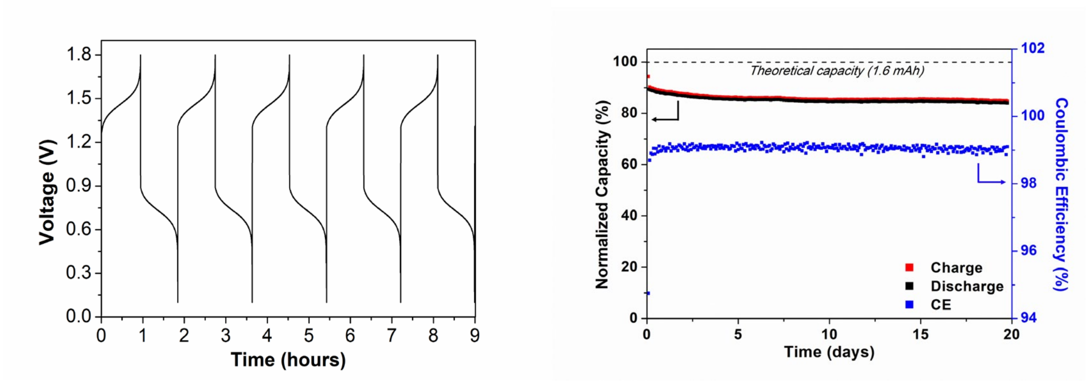


**Figure S60:** (left) Charge and discharge voltage curves for the first 5 cycles of a symmetrical non-flowing battery with 12 mM **3a** in 0.3 M $[Bu_4N][PF_6]/CH_3CN$ as both negolyte and posolyte (cutoff voltages of 1.8 V and 0.1 V for charging and discharging, respectively, current ±1.6 mA, current density 1.0 mA/cm$^2$). (right) Normalized charging and discharging capacities

(theoretical capacity of 1.6 mAh, 94% capacity reached in first charge) and coulombic efficiency for each cycle (277 cycles).

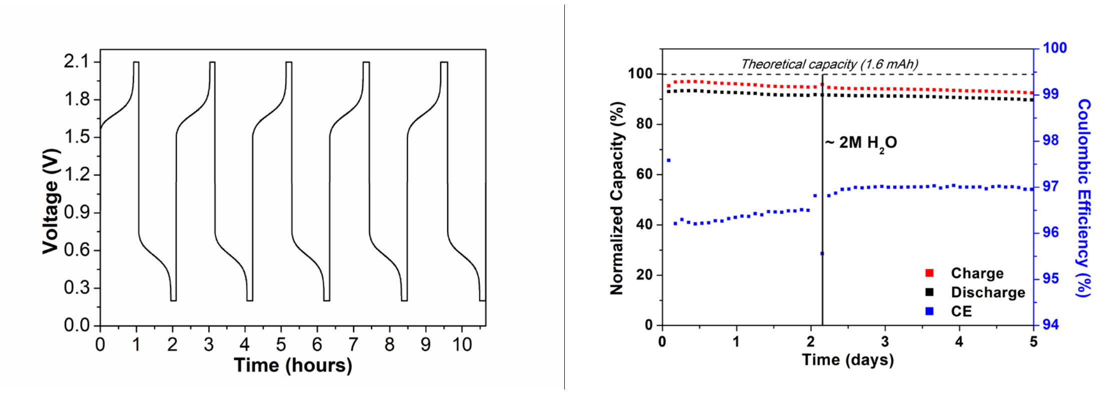


**Figure S61:** (left) Charge and discharge voltage curves for the first 5 cycles of a symmetrical non-flowing battery with 12 mM **3a** in 0.3 M $[Bu_4N][PF_6]/CH_3CN$ as both negolyte and posolyte (cutoff voltages of 2.1 V and 0.2 V for charging and discharging, respectively, current ±1.6 mA, current density 1.0 $mA/cm^2$). (right) Normalized charging and discharging capacities and coulombic efficiency for each cycle, ~2 M $H_2O$ added after 23 cycles.

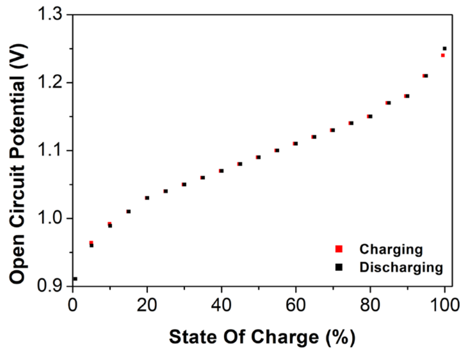


**Figure S62:** OCP-SOC plot of a symmetrical non-flowing battery with 12 mM **3a** in 0.3 M $[Bu_4N][PF_6]/CH_3CN$ as both negolyte and posolyte. The cell was charged and discharged using an intermittent constant current charging protocol during which the OCP was measured after an increase/decrease of SOC with 5%. The cell would be considered fully charged to 100% SOC upon the completion of the constant-current charging protocol, and discharged to 0% SOC upon completion of the constant-current discharging protocol. The average value of charge and discharge OCP was found to be 1.09 V (expected cell potential from CV is 1.12V).

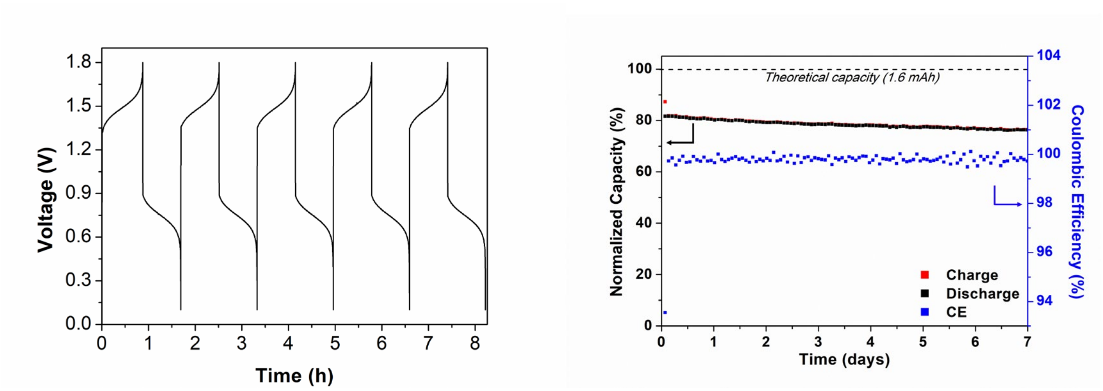


**Figure S63:** (left) Charge and discharge voltage curves for the first 5 cycles of a symmetrical non-flowing battery with 12 mM **3b** in 0.3 M $[Bu_4N][PF_6]/CH_3CN$ as both negolyte and posolyte (cutoff voltages of 1.8 V and 0.1 V for charging and discharging, respectively, current ±1.6 mA, current density 1.0 $mA/cm^2$). (right) Normalized charging and discharging capacities (theoretical capacity of 1.6 mAh, 87% capacity reached in first charge) and coulombic efficiency for each cycle (108 cycles).

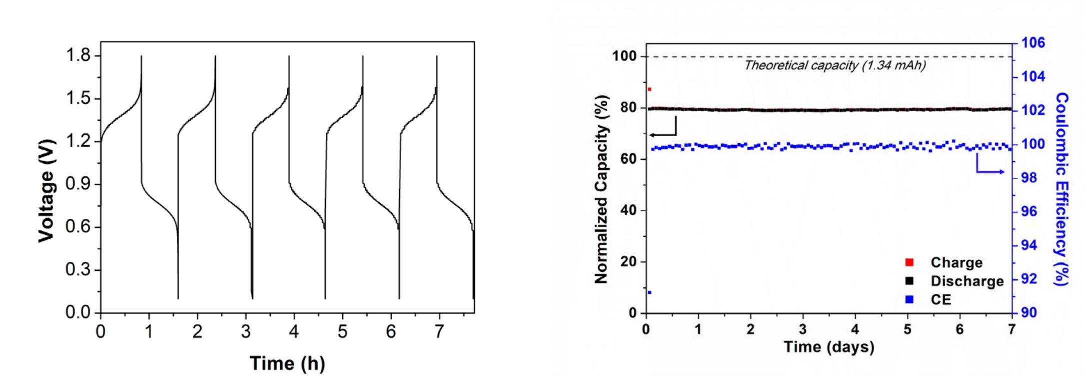


**Figure S64:** (left) Charge and discharge voltage curves for the first 5 cycles of a symmetrical non-flowing battery with 10 mM **3c** in 0.3 M $[Bu_4N][PF_6]/CH_3CN$ as both negolyte and posolyte (cutoff voltages of 1.8 V and 0.1 V for charging and discharging, respectively, current ±1.4 mA, current density 0.9 $mA/cm^2$). (right) Normalized charging and discharging capacities (theoretical capacity of 1.34 mAh, 87% capacity reached in first charge) and coulombic efficiency for each cycle (111 cycles).

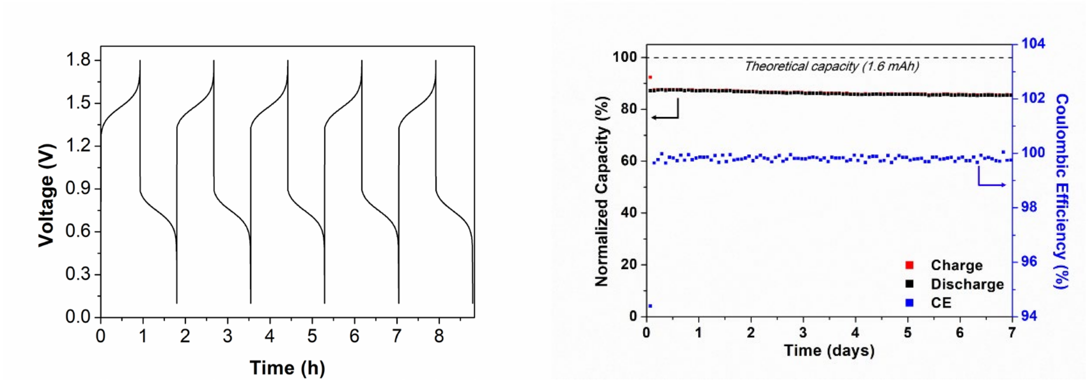


**Figure S65:** (left) Charge and discharge voltage curves for the first 5 cycles of a symmetrical non-flowing battery with 12 mM **3d** in 0.3 M $[Bu_4N][PF_6]/CH_3CN$ as both negolyte and posolyte (cutoff voltages of 1.8 V and 0.1 V for charging and discharging, respectively, current ±1.6 mA, current density 1.0 $mA/cm^2$). (right) Normalized charging and discharging capacities

(theoretical capacity of 1.6 mAh, 92% capacity reached in first charge) and coulombic efficiency for each cycle (110 cycles).

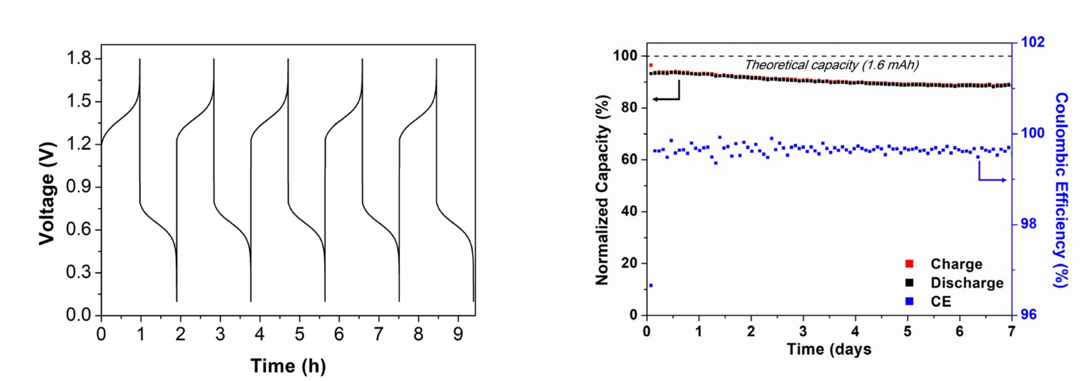


**Figure S66:** (left) Charge and discharge voltage curves for the first 5 cycles of a symmetrical non-flowing battery with 12 mM **3e** in 0.3 M $[Bu_4N][PF_6]/CH_3CN$ as both negolyte and posolyte (cutoff voltages of 1.8 V and 0.1 V for charging and discharging, respectively, current ±1.6 mA, current density 1.0 mA/cm$^2$). (right) Normalized charging and discharging capacities (theoretical capacity of 1.6 mAh, 96% capacity reached in first charge) and coulombic efficiency for each cycle (94 cycles).

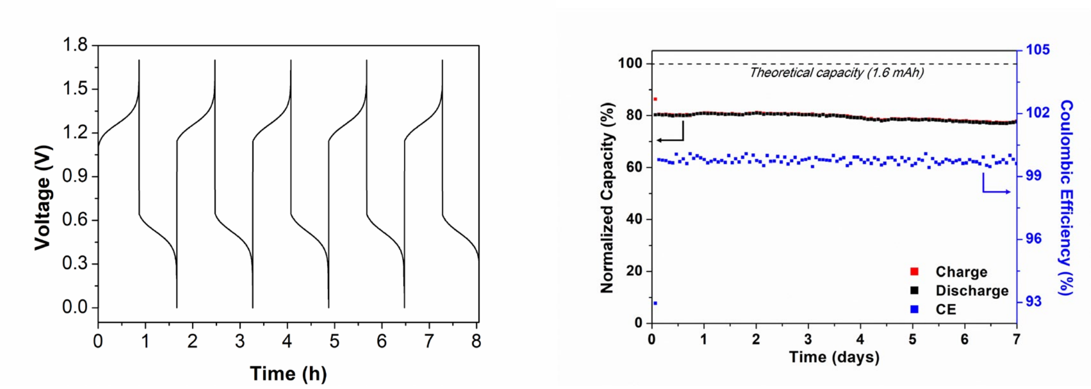


**Figure S67:** (left) Charge and discharge voltage curves for the first 5 cycles of a symmetrical non-flowing battery with 12 mM **3f** in 0.3 M $[Bu_4N][PF_6]/CH_3CN$ as both negolyte and posolyte (cutoff voltages of 1.7 V and 0 V for charging and discharging, respectively, current 1.6 mA, current density 1.0 mA/cm$^2$). (right) Normalized charging and discharging capacities (theoretical capacity of 1.6 mAh, 86% capacity reached in first charge) and coulombic efficiency for each cycle (107 cycles).

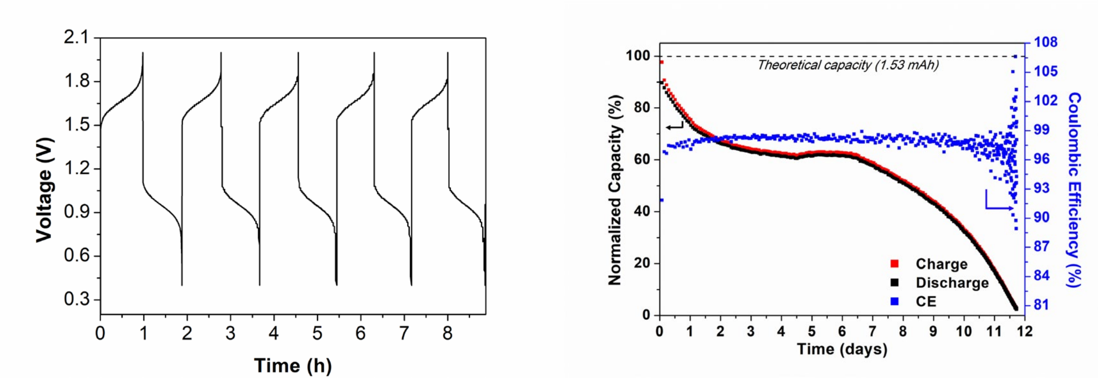


**Figure S68:** (left) Charge and discharge voltage curves for the first 5 cycles of a symmetrical non-flowing battery with 11.4 mM **3h** in 0.3 M $[Bu_4N][PF_6]/CH_3CN$ as both negolyte and posolyte (cutoff voltages of 2.0 V and 0.4 V for charging and discharging, respectively, current 1.53 mA, current density 1.0 $mA/cm^2$). (right) Normalized charging and discharging capacities (theoretical capacity of 1.53 mAh, 96% capacity reached in first charge) and coulombic efficiency for each cycle (365 cycles).

**Table S4:** Average discharge capacity fade rates for the symmetrical non-flowing batteries.

| Radical | Discharge capacity fade (% per day) | Discharge capacity fade (% per cycle) |
|---|---|---|
| **1** | 2.0 | 0.13 |
| **2b** | 3.6 | 0.22 |
| **3a** | 0.3 | 0.02 |
| **3b** | 0.9 | 0.06 |
| **3c** | -[a] | -[a] |
| **3d** | 0.3 | 0.02 |
| **3e** | 0.7 | 0.05 |
| **3f** | 0.5 | 0.03 |

[a] no decay is discernable after 111 charge/discharge cycles (7 days)

### Cycling behavior with polarity inversion

To provide evidence for the 'poleless' nature of a battery based on **3a** as the active material, a charge/discharge experiment was carried out in an H-cell as described above but with inversion of polarity after every 5 charge/discharge cycles.

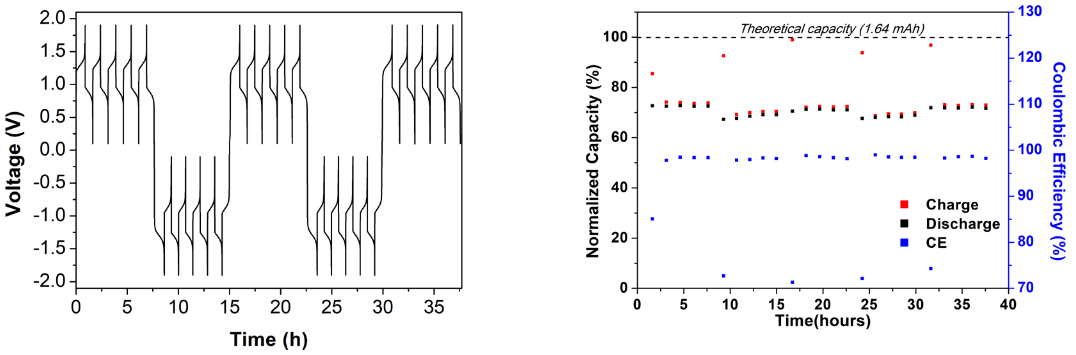


**Figure S69.** (left) Charge and discharge voltage curves for a symmetrical non-flowing battery with 6 mM **3a** in 0.3 M $[Bu_4N][PF_6]/CH_3CN$ as both negolyte and posolyte (10 ml in each compartment), with polarity inversion after every 5 cycles (cutoff voltages of ±1.9 V and ±0.1 V for charging and discharging, respectively,). (right) Normalized charging and discharging capacities (theoretical capacity of 1.64 mAh, 85% capacity reached in first charge) and coulombic efficiency for each cycle.

### Cycling behavior at higher concentration

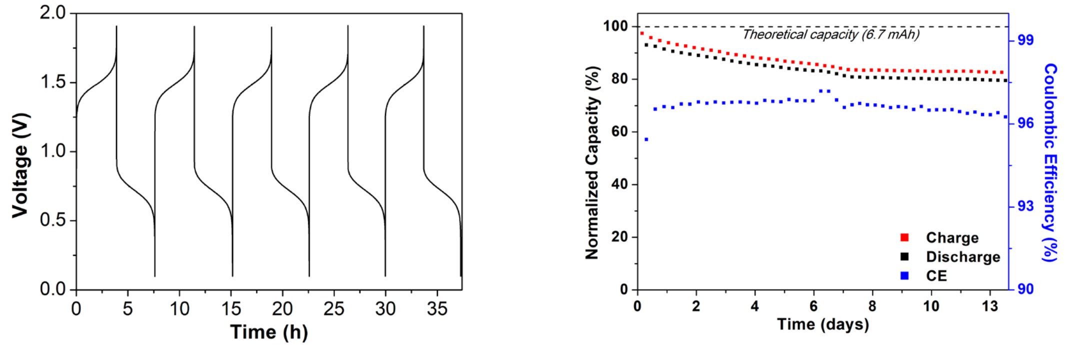


**Figure S70.** (left) Charge and discharge voltage curves for a symmetrical non1.53/1-flowing battery with 50 mM **3a** in 0.3 M $[Bu_4N][PF_6]/CH_3CN$ as both negolyte and posolyte (5 ml in each compartment, cutoff voltages of 1.9 V and 0.1 V for charging and discharging, respectively, current 1.68 mA, current density 1.0 mA/cm$^2$). (right) Normalized charging and discharging capacities (theoretical capacity of 6.7 mAh, 92% capacity utilization reached in first cycle) and coulombic efficiency for each cycle.

## Redox Flow Battery Testing

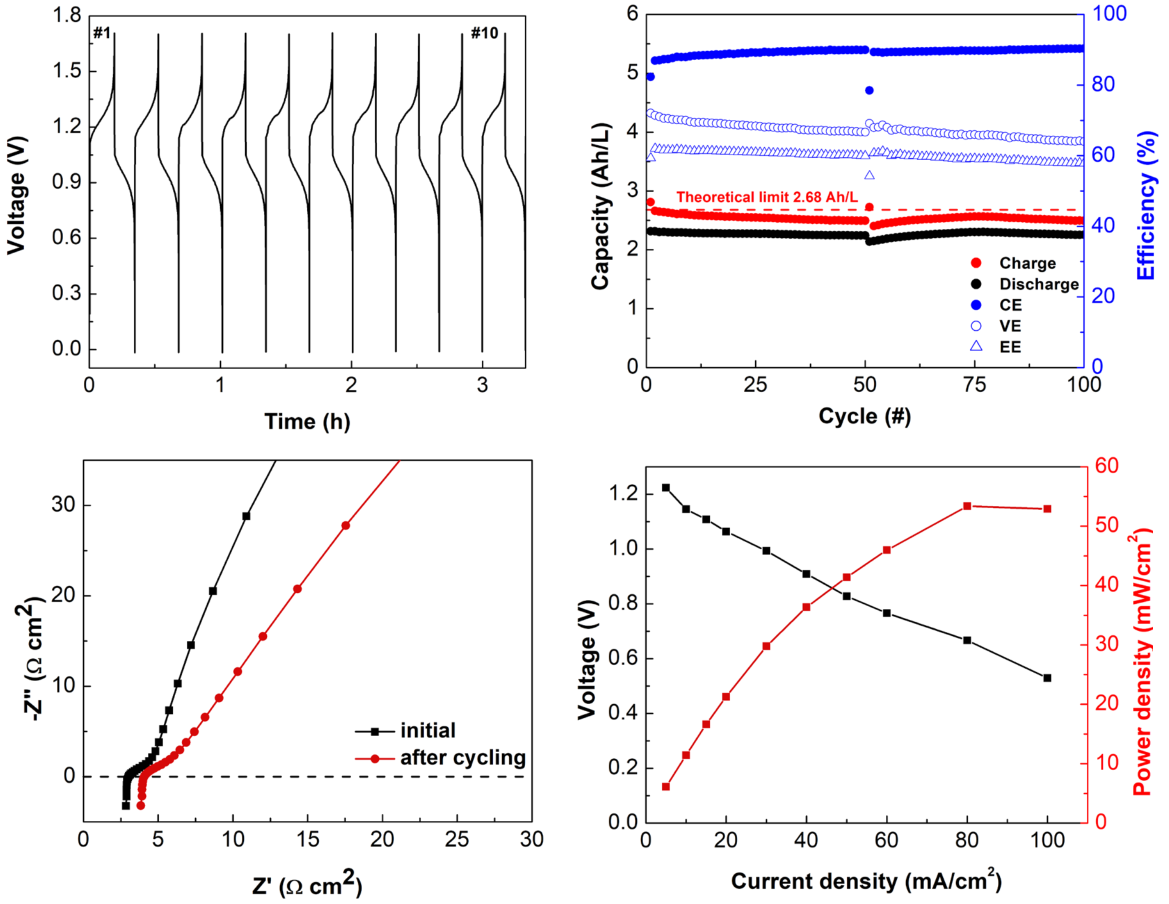


**Figure S71.** Cycling performance of a redox flow battery with 0.1 M **3a** in 0.3 M $[Bu_4N][PF_6]/CH_3CN$ as both negolyte and posolyte (6 mL in each compartment) and a Daramic® (175 µM) porous separator with polarity inversion after 50 cycles. (top left) Voltage versus time curve for the first 10 cycles. (top right) Charge and discharge capacity (86% capacity utilization reached in first cycle of the theoretical capacity of 2.68 Ah/L), and CE, VE, and EE for each cycle. (bottom left) Potential electrochemical impedance spectroscopy (PEIS) before and after cycling (at 0**%** SOC). The first intercept with the Z'-axis represents the ohmic resistance originating from cables, membrane, and solution (initial 3 Ω $cm^2$; after cycling 4.1 Ω $cm^2$). (bottom right) I-V polarization and power density at 100% SOC.

The average area-specific resistance (ASR) of the battery was estimated from the OCV (1.12 V), average cell discharge voltage, and the current density (0.035 $A/cm^2$). We found that the battery resistance only increased slightly during cycling when comparing the resistance of the first cycle (7.8 Ω $cm^2$) with that of the last cycle (9.1 Ω $cm^2$).

## Maximum Solubility Determination by UV-VIS Spectroscopy

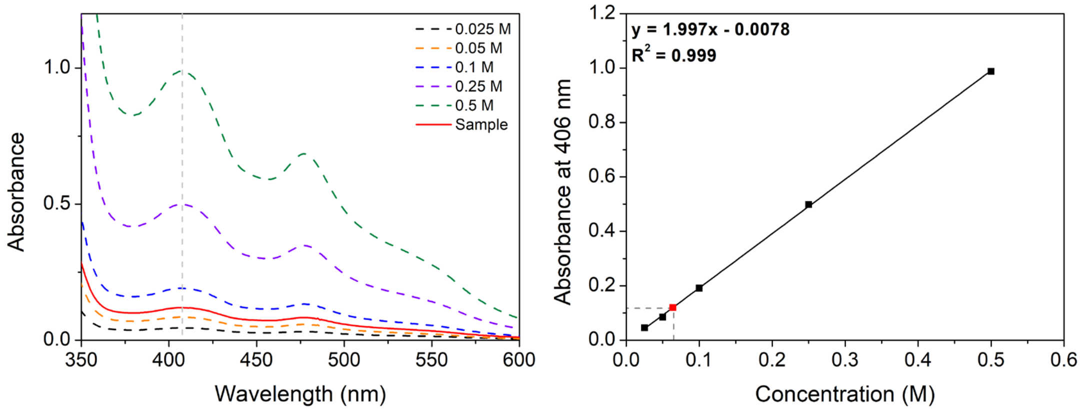


**Figure S72:** (left) Absorption spectra of **3a** in $CH_3CN$ at various concentrations. (right) Calibration curve based on the peak absorbance maximum at 406 nm; red data point indicating sample concentration.

A calibration curve was constructed by recording spectra of five $CH_3CN$ solutions containing **3a** in various known concentrations (0.5 M, 0.25 M, 0.1 M, 0.05 M and 0.025 M). The values of the peak absorbance maxima at 406 nm and corresponding values of concentrations were subjected to linear fitting procedure to yield a linear calibration equation.

A saturated solution of **3a** (20 mg) in 20 μL $CH_3CN$ was prepared. 1 μL of the saturated solution was isolated and diluted to 10 mL $CH_3CN$. A spectrum was recorded of the diluted sample solution. According to the calibration curve the maximum solubility of **3a** in $CH_3CN$ was determined to be 0.64 M.

## Determination of Diffusion Coefficients and Standard Rate Constants by CV

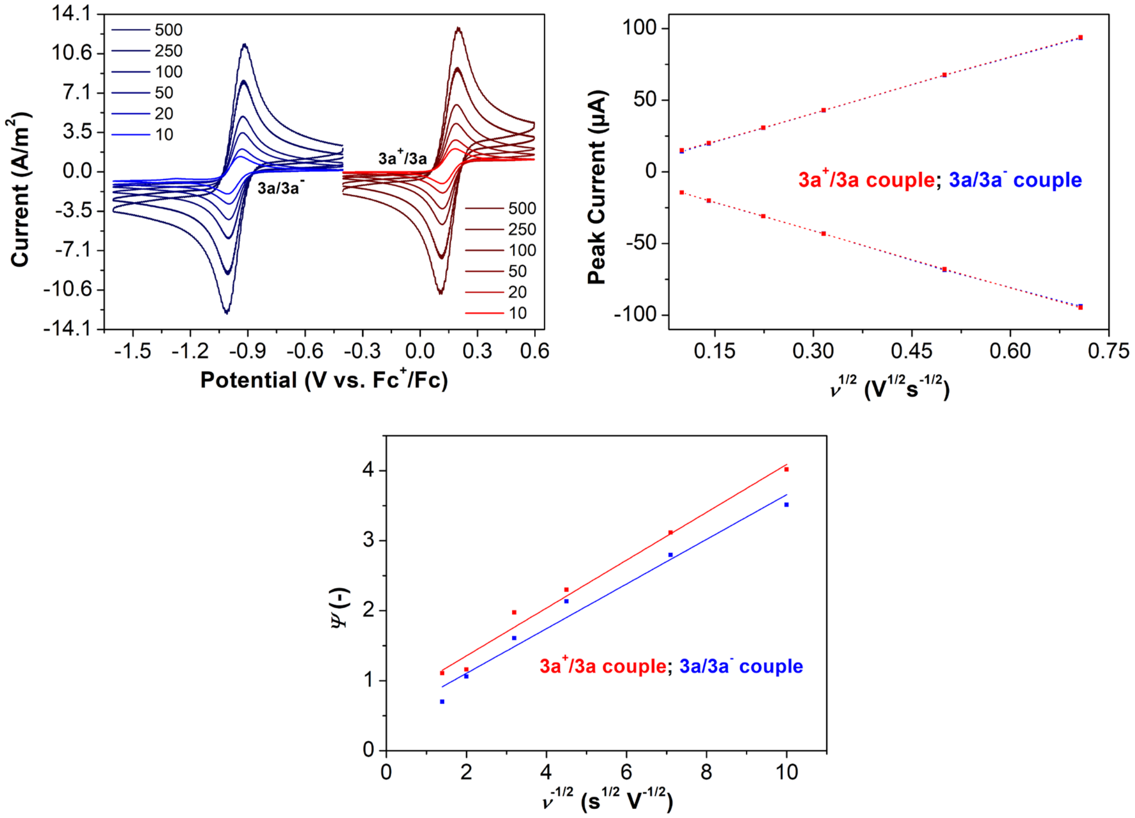


**Figures S73:** (left) Cyclic voltammograms of 2 mM solution of **3a** in 0.1 M $[NBu_4][PF_6]/CH_3CN$ at varying scan rates (from 10 to 500 mV/s). (right) Peak currents ($i_{pc}$ and $i_{pa}$) from Figure S73 (left) vs. $v^{1/2}$ used to determine the diffusion coefficients listed in Table S5. Lines are least-squares fits to the data. (bottom) $\Psi$ (see Equation (S2) in the General Considerations section) vs. $v^{-1/2}$ used to determine the electron transfer rate constants $k^0$ listed in Table S5. Lines are least-squares fits to the data.

**Table S5.** Diffusion coefficients and standard rate constants.

| Compound | D($x^{0/-}$) (× $10^{-5}$ cm²/s) | D($x^{0/+}$) (× $10^{-5}$ cm²/s) | $k^0$ ($x^{0/-}$) (× $10^{-2}$ cm/s) | $k^0$ ($x^{0/+}$) (× $10^{-2}$ cm/s) |
|---|---|---|---|---|
| **1** | 1.2 | 1.5 | 1.3 | 1.8 |
| **2a** | 0.9 | 1.0 | 0.9 | 1.5 |
| **2b** | 1.2 | 1.3 | 1.9 | 1.6 |
| **3a** | 1.2 | 1.2 | 1.2 | 1.3 |
| **3d** | 0.7 | 0.9 | 0.9 | 1.0 |
| **3e** | 0.8 | 1.0 | 0.8 | 1.7 |
| **3f** | 0.9 | 1.0 | 1.0 | 1.0 |

**Table S6. Comparison of this Work with reported symmetrical non-aqueous redox flow batteries.**

| Bipolar Electrolytes | Concentration of transferable electrons (M) | Theoretical cell voltage (V) | Realized theoretical energy density (Wh/L)[a] | Maximum theoretical energy density (Wh/L)[a][b] | Cycles | Capacity loss rate (%/cycle, %/day)[c] | CE, EE (%) |
|---|---|---|---|---|---|---|---|
| **PTIO[12]** | 0.1 | 1.73 | 2.3 | | 35 | 1.4[g], - | 96, 72 |
| | 0.5 | | 11.6 | | 15 | 4.3[g], ~400[g] | 90, 60 |
| | 2.6[d] | | | 60.3 | | | |
| **Porphyrin[13]** | 0.65[e] | 2.83 | 24.7 | | 200 | 0.02[f], 0.006[g] | 99, 41 |
| | 0.02[d] | | | 0.76 | | | |
| **Oxoverdazyl[14]** | 0.01 | 1.42 | 0.19[f] | | 150 | 0.8[g][h], 75[g][h] | 97, - |
| | 2.4[d] | | | 45.7 | | | |
| **FcMeAAQ[15]** | 0.01 | 1.42 | 0.19[f] | | 100 | 0.4[g], 24[g] | 91, 82 |
| | 0.016[d] | | | 0.30 | | | |
| **Benzoylpyridinium[16]** | 0.1 | 0.50[e] | 0.7[f] | | 150 | 0.0028[f][i], 0.19[g][i] | - |
| **VIODAMB[17]** | 0.1 | 1.58 | 2.1[f] | | 21 | 3[f], - | 90[j], 75[j] |
| | 0.66[d] | | | 14.0 | | | |
| **Nitronyl Nitroxides[18]** | 0.2 | 1.62 | 4.3[f] | | 20 | 2[f], - | - |
| | 7.6[d] | | | 165.0 | | | |
| **PDI-TEMPO[19]** | 1.0 | 1.90[e] | 25.5[f] | | 100 | 0.6[f], - | 90, - |
| | 2.6[d] | | | 66.2 | | | |
| **Croconate Violet[20]** | 0.01 | 1.82 | 0.24[f] | | 10 | 9[f], - | 72[g], - |
| | 1.0[d] | | | 24.4 | | | |
| **Fc4Ph-TFSI[21]** | 0.05 | 2.04 | 1.4[f] | | 50 | 0.2[f], 40[g] | 95, 77 |
| | 0.5 | | 14.7[f] | | 20 | 2.5[f], ~140[g] | 87, 50 |
| | 2.21[d] | | | 60.4 | | | |
| **This Work (3a)** | 0.1 | 1.12 | 1.5 | | 100 | 0.02, 1.5 | 89, 60 |
| | 0.64[k] | | | 9.6 | | | |

[a] (concentration of transferable electrons (mol/L))×(theoretical cell voltage (V))×(26.8 (Ah/mol))/(2 electrolyte volumes)) = theoretical overall energy density of the electrolyte system (Wh/L).

[b] Calculated using the maximum solubility data provided in the reference.

[c] Based on the discharge capacities.

[d] Based on the maximum solubility provided in the reference.

[e] RFB cycling performed with a porphyrin-based suspension electrolyte.

[f] Calculated using the data provided in the reference.

[g] Calculated using the data provided in the reference and additional assumptions/interpretation.

[h] Based on the first 80 of the 150 cycles of the RFB cycling test.

[i] Based on the last 100 of the 150 cycles of the RFB cycling test.

[j] Based on the first 10 of the 20 cycles of the RFB cycling test.

[k] Calculated using the maximum solubility of **3a**.

## References


1. Koutentis, P.; Lo Re, D., Catalytic Oxidation of *N*-Phenylamidrazones to 1,3-Diphenyl-1,4-dihydro-1,2,4-benzotriazin-4-yls: An Improved Synthesis of Blatter's Radical. *Synthesis* **2010,** *2010* (12), 2075-2079.
2. Grant, J. A.; Lu, Z.; Tucker, D. E.; Hockin, B. M.; Yufit, D. S.; Fox, M. A.; Kataky, R.; Chechik, V.; O'Donoghue, A. C., New Blatter-type radicals from a bench-stable carbene. *Nat Commun* **2017,** *8*, 15088.
3. Nicholson, R. S., Theory and Application of Cyclic Voltammetry for Measurement of Electrode Reaction Kinetics. *Analytical Chemistry* **1965,** *37* (11), 1351-1355.
4. Stoll, S.; Schweiger, A., EasySpin, a comprehensive software package for spectral simulation and analysis in EPR. *J Magn Reson* **2006,** *178*, 42-55.
5. Milshtein, J. D.; Kaur, A. P.; Casselman, M. D.; Kowalski, J. A.; Modekrutti, S.; Zhang, P. L.; Harsha Attanayake, N.; Elliott, C. F.; Parkin, S. R.; Risko, C.; Brushett, F. R.; Odom, S. A., High current density, long duration cycling of soluble organic active species for non-aqueous redox flow batteries. *Energy Environ. Sci.* **2016,** *9* (11), 3531-3543.
6. Kalikhman, I. D.; Medvedeva, E. N.; Kushnarev, D. F. Y., T. I.; Lopyrev, V. A., 19F NMR study of fluorinated mono- and diacylhydrazines. *Russ Chem Bull* **1980,** *29*, 1911-1914.
7. Constantinides, C. P.; Berezin, A. A.; Zissimou, G. A.; Manoli, M.; Leitus, G. M.; Bendikov, M.; Probert, M. R.; Rawson, J. M.; Koutentis, P. A., A magnetostructural investigation of an abrupt spin transition for 1-phenyl-3-trifluoromethyl-1,4-dihydrobenzo[e][1,2,4]triazin-4-yl. *J Am Chem Soc* **2014,** *136* (34), 11906-11909.
8. Tanaka, K.; Igarashi, T.; Mitsuhashi, K., SYNTHESIS OF 2,2,2-TRISUBSTITUTED 5-TRIFLUOROMETHYL-Δ4-1,3,4,2-OXADIAZAPHOSPHOLINES AND THEIR POTENTIALITY AS PRECURSORS OF TRIFLUOROACETONITRILE IMINES. *Chemistry Letters* **1983,** *12* (4), 507-510.
9. Bruker., *APEX3, SAINT and SADABS. Bruker AXS Inc., Madison, Wisconsin, USA.* **2016**.
10. Sheldrick, G. M., A short history of SHELX. *Acta Cryst A* **2008,** *64* (Pt 1), 112-22.
11. Sheldrick, G. M., Crystal structure refinement with SHELXL. *Acta Cryst C* **2015,** *71* (Pt 1), 3-8.
12. Duan, W.; Vemuri, R. S.; Milshtein, J. D.; Laramie, S.; Dmello, R. D.; Huang, J.; Zhang, L.; Hu, D.; Vijayakumar, M.; Wang, W.; Liu, J.; Darling, R. M.; Thompson, L.; Smith, K.; Moore, J. S.; Brushett, F. R.; Wei, X., A symmetric organic-based nonaqueous redox flow battery and its state of charge diagnostics by FTIR. *Journal of Materials Chemistry A* **2016,** *4* (15), 5448-5456.
13. Ma, T.; Pan, Z.; Miao, L.; Chen, C.; Han, M.; Shang, Z.; Chen, J., Porphyrin-Based Symmetric Redox-Flow Batteries towards Cold-Climate Energy Storage. *Angewandte Chemie International Edition* **2018,** *57* (12), 3158-3162.

14. Korshunov, A.; Milner, M. J.; Grünebaum, M.; Studer, A.; Winter, M.; Cekic-Laskovic, I., An oxo-verdazyl radical for a symmetrical non-aqueous redox flow battery. *Journal of Materials Chemistry A* **2020,** *8* (42), 22280-22291.
15. Zhen, Y.; Zhang, C.; Yuan, J.; Zhao, Y.; Li, Y., Ferrocene/anthraquinone based bi-redox molecule for symmetric nonaqueous redox flow battery. *Journal of Power Sources* **2020,** *480*, 229132.
16. Hendriks, K. H.; Sevov, C. S.; Cook, M. E.; Sanford, M. S., Multielectron Cycling of a Low-Potential Anolyte in Alkali Metal Electrolytes for Nonaqueous Redox Flow Batteries. *ACS Energy Letters* **2017,** *2* (10), 2430-2435.
17. Liu, B.; Tang, C. W.; Sheong, F. K.; Jia, G.; Zhao, T., Artificial Bipolar Redox-Active Molecule for Symmetric Nonaqueous Redox Flow Batteries. *ACS Sustainable Chemistry & Engineering* **2022,** *10* (1), 613-621.
18. Hagemann, T.; Winsberg, J.; Häupler, B.; Janoschka, T.; Gruber, J. J.; Wild, A.; Schubert, U. S., A bipolar nitronyl nitroxide small molecule for an all-organic symmetric redox-flow battery. *NPG Asia Materials* **2017,** *9* (1), e340-e340.
19. Nambafu, G. S.; Delmo, E. P.; Bin Shahid, U.; Zhang, C.; Chen, Q.; Zhao, T.; Gao, P.; Amine, K.; Shao, M., Pyromellitic diimide based bipolar molecule for total organic symmetric redox flow battery. *Nano Energy* **2022,** *94*, 106963.
20. Armstrong, C. G.; Hogue, R. W.; Toghill, K. E., Application of the dianion croconate violet for symmetric organic non-aqueous redox flow battery electrolytes. *Journal of Power Sources* **2019,** *440*, 227037.
21. Xu, D.; Zhang, C.; Zhen, Y.; Li, Y., Ferrocene/Phthalimide Ionic Bipolar Redox-Active Molecule for Symmetric Nonaqueous Redox Flow Batteries. *ACS Applied Energy Materials* **2021,** *4* (8), 8045-8051.
